# Heterogeneity of Myc expression in breast cancer exposes pharmacological vulnerabilities revealed through executable mechanistic modeling

**DOI:** 10.1073/pnas.1903485116

**Published:** 2019-10-14

**Authors:** Peter Kreuzaler, Matthew A. Clarke, Elizabeth J. Brown, Catherine H. Wilson, Roderik M. Kortlever, Nir Piterman, Trevor Littlewood, Gerard I. Evan, Jasmin Fisher

**Affiliations:** ^a^Department of Biochemistry, University of Cambridge, Cambridge CB2 1GA, United Kingdom;; ^b^Oncogenes and Tumour Metabolism Lab, The Francis Crick Institute, London NW1 1AT, United Kingdom;; ^c^Department of Computer Science and Engineering, University of Gothenburg, SE-41296 Gothenburg, Sweden;; ^d^UCL Cancer Institute, University College London, London WC1E 6DD, United Kingdom

**Keywords:** oncogenic signaling, Myc, cancer heterogeneity, computational modeling, breast cancer

## Abstract

Cells with higher levels of Myc proliferate more rapidly and supercompetitively eliminate neighboring cells. Nonetheless, tumor cells in aggressive breast cancers typically exhibit significant and stable heterogeneity in their Myc levels, which correlates with refractoriness to therapy and poor prognosis. This suggests that Myc heterogeneity confers some selective advantage on breast tumor growth and progression. To investigate this, we created a traceable *MMTV-Wnt1*–driven in vivo chimeric mammary tumor model comprising an admixture of low-Myc– and reversibly switchable high-Myc–expressing clones. We show that such tumors exhibit interclonal mutualism wherein cells with high-Myc expression facilitate tumor growth by promoting protumorigenic stroma yet concomitantly suppress Wnt expression, which renders them dependent for survival on paracrine Wnt provided by low-Myc–expressing clones. To identify any therapeutic vulnerabilities arising from such interdependency, we modeled Myc/Ras/p53/Wnt signaling cross talk as an executable network for low-Myc, for high-Myc clones, and for the 2 together. This executable mechanistic model replicated the observed interdependence of high-Myc and low-Myc clones and predicted a pharmacological vulnerability to coinhibition of COX2 and MEK. This was confirmed experimentally. Our study illustrates the power of executable models in elucidating mechanisms driving tumor heterogeneity and offers an innovative strategy for identifying combination therapies tailored to the oligoclonal landscape of heterogenous tumors.

## Significance

Breast cancer remains a leading cause for cancer-related mortality worldwide. All breast cancers, including the more difficult-to-treat, higher-grade, and triple-negative subtypes of breast cancer, exhibit strong genetic heterogeneity, which hampers treatment and fuels relapse. Our study advances the development of successful treatment approaches by unravelling the mechanistic basis of one form of heterogeneity arising from mutualism between high- and low-Myc–expressing clones in breast cancer. We use this mechanistic understanding to build an executable *in silico* model of oncogenic Myc/Ras/p53/Wnt signal cross talk for each Myc-expressing clone, separately and together, and then use this model to identify potential therapeutic vulnerabilities, which we then verify experimentally.

Most solid tumors exhibit extensive intratumoral genetic heterogeneity (1–3) and comprise multiple clones whose identities and prominence shift between primary tumors, metastatic colonies, and relapse after therapy. Such heterogeneity fuels tumor evolution and contributes to the failure of durable therapeutic responses and to subsequent relapse (4, 5). In breast cancers, distant breast cancer metastases often comprise multiple clones from the primary tumor (6), suggesting that certain polyclonal ensembles may be advantageous, and perhaps necessary, for metastatic dissemination, persistence, and outgrowth (7). In line with these observations, murine models of breast cancer have been reported to show mutualism between genetically distinct clones that enhances tumor growth in a concerted fashion (8, 9). Hence, while tumor heterogeneity often confounds successful therapy, interclonal dependencies might yet exist that create novel therapeutic vulnerabilities (9).

The Myc transcription factor is a key coordinator of somatic cellular proliferation and regeneration. In normal somatic cells, Myc activity is tightly controlled and dependent upon mitogenic signals, whereupon it drives cells into proliferation along with metabolic transition to biosynthesis, varying degrees of dedifferentiation, and co-option through signals of stromal, inflammatory, and immune compartments (10). Oncogenic deregulation of Myc, which hijacks this regenerative program, is evident in most, perhaps all, cancers. In breast cancers, Myc is one of the most frequently overexpressed genes (11), especially in higher-grade regions of such tumors. However, high-Myc–expressing tumor cells do not typically dominate the growing tumor mass in breast cancer but are instead interspersed among tumor cells expressing lower levels of Myc (12–16). Such stable and persistent Myc heterogeneity is surprising since Myc is one of several genes reported to elicit supercompetitive behavior. When precociously activated, such supercompetitive genes not only drive cells to outproliferate their neighbors but also to actively induce their neighbors’ demise through, as yet, poorly understood mechanisms that appear to require direct cell contact (17). However, evidence for Myc-driven supercompetition in mammalian cancers remains sparse and so far has only been observed at the boundaries of neoplastic lesions where tumor cells may be killing adjacent healthy tissues (18). The net outcome of high-Myc expression is further complicated by the fact that cells expressing elevated levels of Myc are greatly predisposed to apoptosis, which self-limits their expansion. These 2 antagonistic properties of elevated Myc expression—supercompetition vs. apoptosis—make it difficult to predict the fates of high- vs. low-Myc–expressing cancer cells during tumor evolution in vivo.

The heterogeneity of Myc expression observed in breast cancers may indicate some novel mechanism acts to maintain stable clonal variation in Myc levels within the tumor cell population. On the other hand, it could also be a snapshot illusion arising from fluctuating Myc levels in individual cells over time. To explore these possibilities, we constructed a unique estrogen-negative mammary carcinoma mouse model in which tumor cell clones expressing high vs. low preset levels of Myc are tested for tumorigenic efficacy, separately and together, using a combination of experiment and *in silico* executable modeling of the intracellular oncogenic signaling network.

## Results

### Generation of Genetically Engineered Mice Allowing for Both Switchable and Heterogeneous Myc Expression in Wnt-Driven Mammary Cancer.

To determine the impacts of different levels of Myc expression on mammary tumors, we used the well-characterized *MMTV-Wnt1*-driven [*B6SJL-Tg*(*Wnt1*)*1Hev/J*] (19) mouse model of mammary carcinoma. This was crossed into the *R26*^*CAG-LSL-MycERT2*^ (*R26C*^*LSL-MER*^) and *R26*^*mTmG*^ [*Gt*(*ROSA*)*26Sor*^*tm4(ACTB-tdTomato,-EGFP)Luo*^] reporter backgrounds (20). After Cre-mediated excision of the *LSL* transcriptional STOP element, *R26C*^*MER*^ allele constitutively expresses the 4-hydroxytamoxifen (4OHT)-dependent allele of Myc, MycER^T2^, at supraphysiological (∼6 to 10× physiological) levels. In addition, Cre recombination toggles the constitutive *R26*^*mTmG*^ allele from red (Tomato) to green (EGFP) (*SI Appendix*, Fig. S1*A*). The genotype of the resultant *MMTV-Wnt1; R26C*^*LSL-****M****ER*^*; R26*^*m****T****mG*^ mice was designated *WMT*.

*MMTV-Wnt1* tumors occasionally develop estrogen receptor (ER)-positive tumors. However, these rapidly switch to an ER-negative phenotype in response to sustained tamoxifen treatment (21). Therefore, to obviate any complexities arising from direct action of tamoxifen (used to trigger MycER^T2^ activation) on *WMT* mammary via endogenous estrogen receptors, we first converted all *MMTV-Wnt–*induced tumors to ER-negative status by pretreating tumor-bearing *MMTV-Wnt1; R26C*^*LSL-MER*^*; R26*^*mTmG*^ mice with tamoxifen prior to their deployment in serial transplantation studies. ER negativity of treated mammary tumors was confirmed by immunohistochemistry (IHC) (*SI Appendix*, Fig. S1*B*). Furthermore, transplanted tumors exhibited no discernible changes in tumor cellularity, necrosis, proliferation, and incidence of cell death following tamoxifen treatment (*SI Appendix*, Fig. S1*C*). ER-negative *WMT* tumor cells were then infected ex vivo with adenovirus-CRE, which triggered efficient recombination and activation of both *R26C*^*LSL-MER*^ and *R26*^*mTmG*^ alleles (*SI Appendix*, Fig. S1*D*). These recombined tumor cells were then flow-sorted into green MycER^T2^-positive (*WM*^*+*^*T*) and red MycER^T2^-negative (*WM*^*−*^*T*) populations and injected, either separately or mixed together into the fat pads of recipient SCID mice. Tumors were then allowed to grow to around 1 cm^3^ before treating mice with tamoxifen to activate MycER^T2^.

Our tumor model depends on 2 concurrent Cre-mediated recombinations in each targeted cell—one to activate MycER^T2^ and the other to induce the switch from (red) Tomato to (green) EGFP. Analysis via flow cytometry of outgrown tumors revealed a small population of GFP/TdTomato double-positive cells in *WM*^*+*^*T* tumors, which are the likely result of a monoallelic recombination on the *R26C*^*LSL-MER*^ only (*SI Appendix*, Fig. S1*E*). Conversely, genomic analysis on the respective tumors showed that *WM*^*+*^*T* tumors had very small amounts of unrecombined *R26C*^*LSL-MER*^ (*SI Appendix*, Fig. S1*F*). As expected, *WM*^*−*^*T* tumors did not show recombination at any allele of either of the 2 transgenic loci (*SI Appendix*, Fig. S1 *E* and *F*). We also compared the expression levels of MycER^T2^ driven by the recombinant *R26C*^*LSL-MER*^ allele to that of endogenous Myc. Only around a quarter of cells in *WM*^*−*^*T* tumors had detectable levels of Myc. Despite a stark reduction in the levels of endogenous Myc upon MycER^T2^ activation in *WM*^*+*^*T* tumors, almost every cell in these tumors retained overall Myc levels that are higher than those seen in *WM*^*−*^*T* tumor cells (*SI Appendix*, Fig. S1 *G*–*I*).

### Low vs. High Levels of Myc in MMTV-Wnt–Driven Mammary Tumors Exhibit Distinct Behaviors and Dynamics.

**Fig. 1.**
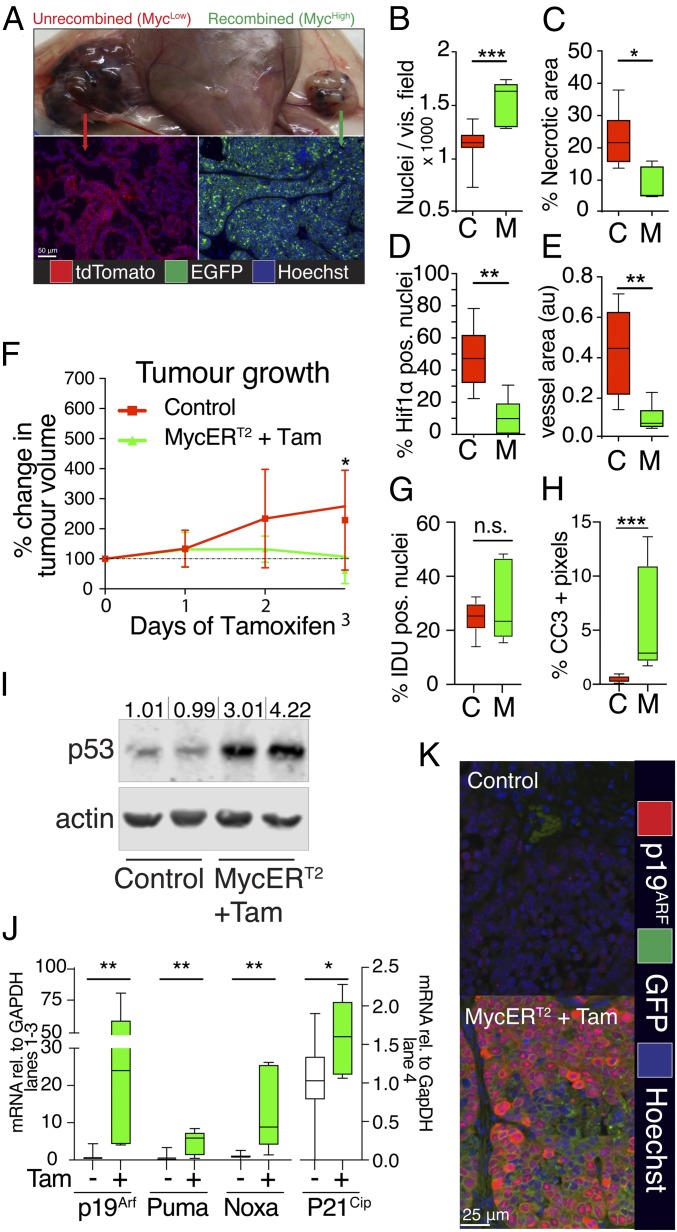
Myc activation leads to rapid reorganization of the tumor and its stroma, while limiting growth through tumor-suppressive pathways. All tumors were analyzed after 3 d of sustained treatment with tamoxifen (100 μg/mouse, twice daily) or oil (vehicle) for the respective controls. Abbreviations in graphs: C, controls; M, Mycer^T2^ plus tamoxifen. Error bars represent SD. (*A*) Gross morphological features and histological appearance of *WM*^*+*^*T* tumors compared to controls (controls in all following experiments are as follows: *WM*^*−*^*T* plus tamoxifen and plus vehicle; *WM*^*+*^*T* plus vehicle). (*B*) Cellularity of *WM*^*+*^*T* tumors and controls (*n* = 6/9, respectively). (*C*) Quantification of necrosis of *WM*^*+*^*T* tumors and controls (*n* = 6/9, respectively). (*D*) Quantification of hypoxia in *WM*^*+*^*T* tumors and controls (*n* = 6/9, respectively). (*E*) Average area of blood vessel, in *WM*^*+*^*T* tumors and controls (*n* = 6/9, respectively). (*F*) Caliperimetric measurement of tumor growth of *WM*^*+*^*T* tumors and controls, as percentage change from start of treatment (*n* = 6 *WM*^*+*^*T* tumors and 11 controls). (*G*) Quantification of proliferation, by IDU incorporation, in *WM*^*+*^*T* tumors and controls (*n* = 7/14 respectively). (*H*) Quantification of cell death, by presence of cleaved caspase 3, in *WM*^*+*^*T* tumors and controls (*n* = 6/9, respectively). (*I*) WB analysis of p53 and actin as a loading control in whole-tissue lysates of 2 representative *WM*^*+*^*T* tumors and controls. Bands were quantified by comparison with the loading control and are represented as fold change relative to average of the control tumors. (*J*) Quantitative real-time PCR of whole-tissue mRNA extracts from *WM*^*+*^*T* tumors and controls for indicated genes and GAPDH as a housekeeping gene (*n* = 6/11, respectively). (*K*) Representative immunofluorescent staining for p19^*ARF*^ (red), EGFP (green), and DNA (Hoechst; blue), of *WM*^*+*^*T* tumors and controls (*n* = 6/9, respectively). For box-and-whisker plots, the error bars represent min to max values, the box represents the interquartile range, and the horizontal line represents the median. *P* values are based on Student’s *t* test: n.s., not significant; *P < 0.05, **P < 0.01, and ***P < 0.001.

To determine the impact of low vs. high Myc expression on mammary tumor dynamics, we first compared the phenotypes of *WM*^*−*^*T* (Myc^low^) and *WM*^*+*^*T* (Myc^low^ without tamoxifen, Myc^high^ with tamoxifen) tumors. Histologically, Myc^low^ (*WM*^*−*^*T*) tumors exhibited a “loose” structure, characterized by low cellularity, and signs of differentiation such as the retention of a recognizable epithelial organization with large luminal spaces separated by sheets of tumor cells (Fig. 1 *A* and *B* and *SI Appendix*, Fig. S1*J*). Generally, they appear to lack the ability to instruct sufficient supportive stroma for their growth, resulting in large areas of necrosis and hemorrhagic cysts surrounded by hypoxic regions as evidenced by the presence of nuclear HIF1α (Fig. 1 *C* and *D* and *SI Appendix*, Fig. S1 *J* and *K*). However, administration of tamoxifen to activate high levels of Myc in transplanted *WM*^*+*^*T* tumors induced profound histological changes. Within 3 d, luminal spaces were completely lost and replaced by tightly packed nests of highly invasive tumor cells (Fig. 1 *A* and *B* and *SI Appendix*, Fig. S1*J*). Myc activation also rapidly induced a profound switch to angiogenesis, marked by extensive vascular remodeling and highlighted by a smaller average vessel size, which correlated temporally with a fall in active nuclear HIF1α and a profound decrease in hemorrhage and necrosis (Fig. 1 *C*–*E* and *SI Appendix*, Fig. S1*K*). Nonetheless, despite these ostensibly-protumorigenic stromal changes, persistent elevation of Myc activity actually retarded, and occasionally reversed, net tumor growth (Fig. 1*F*). Such reduced growth was not associated with any measurable decrease in tumor cell proliferation, whose already high baseline rate was unaffected by MycER^T2^ activation (Fig. 1*G* and *SI Appendix*, Fig. S1*L*). Rather, Myc overexpression dramatically increased the incidence of tumor cell apoptosis, as indicated by the presence of cleaved caspase 3 (CC3) (Fig. 1*H* and *SI Appendix*, Fig. *M* and *N*). Elevated Myc has well-described–proapoptotic activity (22–24) that is, in many instances, facilitated via activation of the p53 tumor suppressor. Both IHC and Western blot (WB) analysis confirmed marked accumulation of p53 in *WM*^*+*^*T* tumor cells, clearly evident by 3 d post-MycER^T2^ activation (Fig. 1*I* and *SI Appendix*, Fig. S1*O*) and accompanied by robust induction of the p53 target genes *Puma*, *Noxa*, and C*dkn1a* (Fig. 1*J*). In mice, Myc-dependent activation of p53 is mediated principally through induction of the p19^*ARF*^ protein, encoded by an alternate *CDKN2A* gene ORF, which acts to inhibit the Mdm2 p53 E3 ubiquitin ligase (25). MycER^T2^ activation induced rapid accumulation of p19^*ARF*^ (Fig. 1 *J* and *K*). Of note, expression of the BH3-encoding gene *BIM*, reported elsewhere to be a direct, p53-independent, downstream BH3 apoptotic effector of Myc, was unaffected by MycER^T2^ activation (*SI Appendix*, Fig. S1 *P* and *Q*) (26). Taken together, these results implicate engagement of a p19^*ARF*^→p53→PUMA/NOXA pathway as the likely apoptotic effector mechanism activated by elevated Myc in Wnt-driven mammary tumors.

### High- and Low-Myc–Expressing Mammary Tumor Cells Exhibit Mutual Interdependence.

**Fig. 2.**
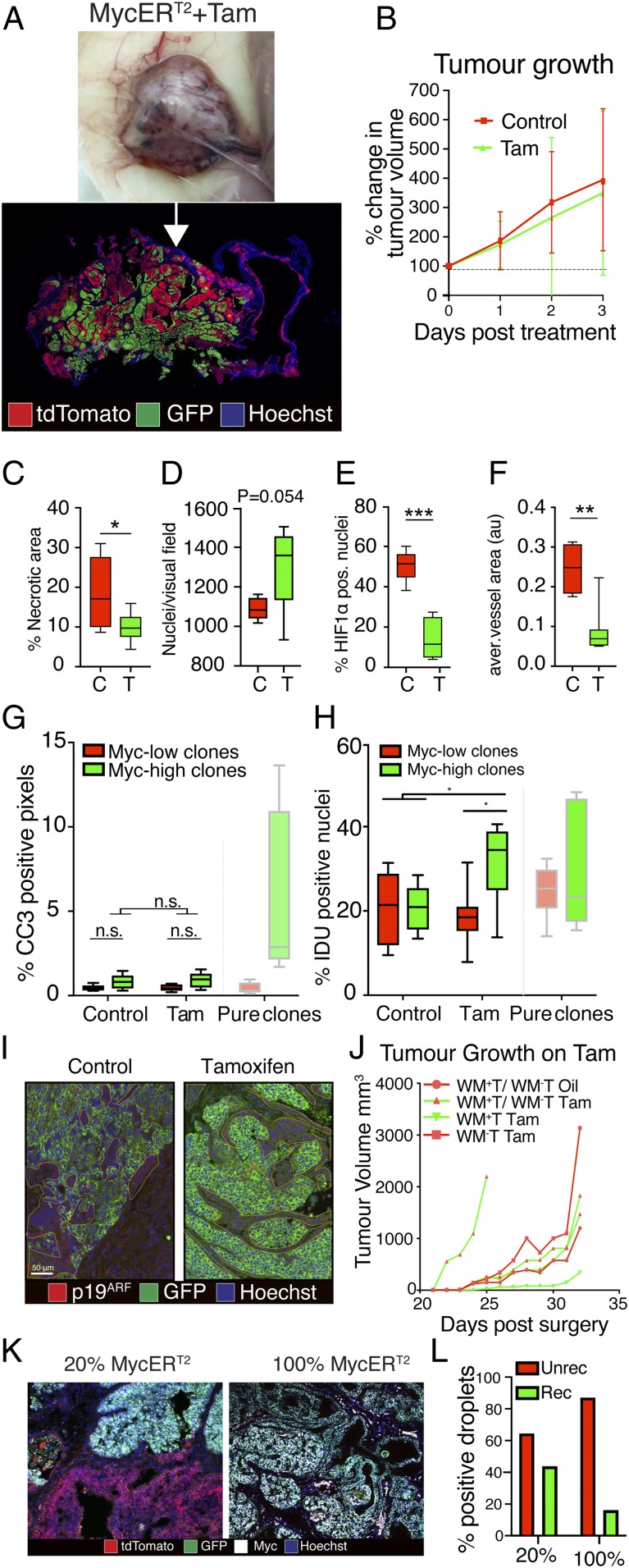
Myc^high^ and Myc^low^ cells exhibit clonal mutual interdependence. Unless otherwise indicated, analysis of mixed *WM*^*+*^*T/WM*^*−*^*T* tumors was carried out after 3 d of treatment with tamoxifen (100 μg/mouse, twice daily) or oil (vehicle) for the respective controls. Abbreviations in graphs: C, mixed tumors plus vehicle; T, mixed tumors plus tamoxifen. Error bars represent SD. (*A*) Representative picture of the gross morphology of mixed (in this case, 30%/70%) *WM*^*+*^*T/WM*^*−*^*T* tumors and representative fluorescent image of mixed tumor tissue. (*B*) Caliperimetric measurement of tumor growth of mixed *WM*^*+*^*T/WM*^*−*^*T* tumors and oil controls. (*C*) Necrotic burden of mixed *WM*^*+*^*T/WM*^*−*^*T* tumors. (*D*) Quantification of the cellularity of mixed *WM*^*+*^*T/WM*^*−*^*T* tumors. (*E*) Quantification of hypoxia in mixed *WM*^*+*^*T/WM*^*−*^*T* tumors. (*F*) Average area of blood vessel in mixed *WM*^*+*^*T/WM*^*−*^*T* tumors. (*G*) Quantification of cell death, by presence of cleaved caspase 3, in mixed *WM*^*+*^*T/WM*^*−*^*T* tumors. Clones were distinguished via GFP staining. (*H*) Quantification of proliferation, by IDU incorporation, in mixed *WM*^*+*^*T/WM*^*−*^*T* tumors. Clones were distinguished via GFP staining. (*I*) Immunofluorescent staining for GFP (*WM*^*+*^*T*) and p19^*ARF*^ of intermingled areas in mixed *WM*^*+*^*T/WM*^*−*^*T* tumors and controls (in *D*–*I*: *n* = 8 mixed *WM*^*+*^*T/WM*^*−*^*T* tumors treated with tamoxifen and 5 vehicle controls). (*J*) Caliperimetric measurement of tumor growth of individual *WM*^*+*^*T, WM*^*−*^*T*, mixed *WM*^*+*^*T/WM*^*−*^*T* mixed tumors, and controls on long-term tamoxifen treatment. (*K*) Representative images of immunofluorescence staining for Myc, dtTomato, and GFP on frozen tissue sections from long-term–treated *WM*^*+*^*T* and mixed *WM*^*+*^*T/WM*^*−*^*T* tumors showing loss of Myc in the *WM*^*+*^*T* tumors, but no such phenomenon in the mixed *WM*^*+*^*T/WM*^*−*^*T* tumors. (*L*) Digital droplet PCR on genomic DNA comparing the recombination status of the *R26C*^*MER*^ allele in 20% *WM*^*+*^*T* and 100% *WM*^*+*^*T* tumor after long-term tamoxifen treatment (*n* = 1 20% *WM*^*+*^*T* and 1 100% *WM*^*+*^*T* tumor). For box-and-whisker plots, the error bars represent min to max values, the box represents the interquartile range, and the horizontal line represents the median. *P* values are based on Student’s *t* test: *P < 0.05, **P < 0.01, and ***P < 0.001. In *I*, the Myc^low^ clone is outlined by a dotted line. Areas that are neither GFP positive nor marked up by dotted lines are nontumor tissues such as stroma and necrotic areas.

Our data from *WM*^*+*^*T* tumors are consistent with previous studies indicating that apoptotic signaling by Myc at high levels self-limits its overall capacity to drive oncogenesis despite its potent proproliferative effects (27, 28). However, this seems at odds with diverse observations that increased Myc gene expression and/or copy number is associated with later-stage, more aggressive breast cancers. It is therefore noteworthy that Myc overexpression or amplification in breast cancers is usually observed in only a subpopulation of cancer cells within individual tumors and that such chimerism in Myc expression level persists through tumor evolution (12, 13, 16). We therefore hypothesized that some selective advantage or mutualism exists to maintain coexistence of Myc^high^ with Myc^low^ tumor cells. To investigate this idea, we generated bespoke biclonal Wnt-driven mammary tumors comprising both Myc^high^ and Myc^low^ tumor cells, by coinjecting a mixture of floxed *WM*^*+*^*T* (20 to 30%) and unfloxed *WM*^*−*^*T* (80 to 70%) clones into the same fat pad. Tumors were then allowed to develop and MycER^T2^ in the *WM*^*+*^*T* cells then activated acutely by tamoxifen administration to generate Myc^high^ tumor cells. After 3 d of tamoxifen or vehicle treatment, mixed tumors showed a wide range of variation in the ratios of the 2 clones, and exhibited a spectrum of chimerism—in some regions one or the other clone predominated, while elsewhere we saw convoluted interfaces between the Myc^high^ and Myc^low^ populations as well as mixing of the Myc^high^ and Myc^low^ clones (Fig. 2*A* and *SI Appendix*, Fig. S2 *A*–*D* and *F*). Importantly, none of these heterogeneous Myc^high^/Myc^low^ tamoxifen-treated tumors showed the self-limitation of growth characteristic of Myc^high^-only tumors (Fig. 2*B*). However, like Myc^high^-only tumors, and quite unlike Myc^low^-only tumors, Myc^high^/Myc^low^ chimeric tumors were invasive, angiogenic, and predominantly normoxic, exhibiting little necrosis and displaying a strong trend toward increased cellularity (*P* = 0.054) (Fig. 2 *C*–*F* and *SI Appendix*, Fig. S2 *B* and *E*). Focusing on areas of Myc^high^/Myc^low^ clonal intermingling where any interclonal cooperation is likely to be most relevant, both tumor necrosis (Fig. 2*C* and *SI Appendix*, Fig. S2*B*) and tumor cell apoptosis (Fig. 2*G* and *SI Appendix*, Figs. S1*N* and S2 *E*, *Bottom*) appeared profoundly suppressed to the low background levels characteristic of Myc^low^-only lesions. Such suppression of necrosis was evident only in tumors of mice in which MycER^T2^ had been activated with tamoxifen (*SI Appendix*, Fig. S2*B*). Moreover, suppression of apoptosis in areas of Myc^high^/Myc^low^ intermingling coincided spatially with suppressed expression of p19^*ARF*^ (Fig. 2*I*) as well as increased proliferation of the Myc^high^ cells over Myc^low^ ones, evident from increased numbers of IDU-positive Myc^high^ nuclei (Fig. 2*H* and *SI Appendix*, Fig. S2*G*). Hence, chimeric regions of Myc^high^/Myc^low^ mammary tumors selectively exhibit the combined protumorigenic attributes of each clone—the aggressive tumor stromal features, angiogenesis, low necrosis, and high cellularity associated with Myc^high^ cells, and the greatly reduced apoptosis exhibited by Myc^low^ tumor cells.

To investigate whether selection over the long term can drive evasion of apoptosis in Myc^high^ tumors, we implanted tumor cells into recipient fat pads. Ten days later, at which time tumors are not yet palpable, we activated MycER^T2^ continuously for over 30 d, by which stage Myc^low^ and Myc^low^/Myc^high^ tumors had grown to a size greater than 1.5 cm^3^. From the outset, Myc^low^ and Myc^low^/Myc^high^ biclonal tumors grew progressively, with kinetics similar to vehicle-treated tumors. Moreover, the mixed clonal tumors retained polyclonality throughout, highlighting the fact that even over a long period of expansion neither Myc^low^ nor Myc^high^ clones outcompeted the other (Fig. 2 *J* and *K*, *Left* and *SI Appendix*, Fig. S2*H*). By contrast, Myc^high^-alone tumors either failed to grow at all or showed a marked delay in growth (Fig. 2*J* and *SI Appendix*, Fig. S2*F*). All those Myc^high^ tumors that did eventually grow out after prolonged Myc activation presented as mixtures of cells with and without MycER^T2^ expression (Fig. 2*K*, *Right*, and *SI Appendix*, Fig. S2*H*). Notably, all of the MycER^T2^-negative cells in these escaping tumors expressed the *Rosa26*^*mTmG*^ encoded membrane-targeted GFP marker, indicating that Cre recombination had occurred in those cells: hence, absence of MycER^T2^ expression is likely to be due to a failure to recombine at both the *MycER*^*T2*^ and *GFP* allele. To test this hypothesis, we performed genomic DNA analysis on one of the 2 outgrowing Myc^high^ tumors and found that it showed a marked presence of unrecombined *R26C*^*LSL-MER*^ allele (Fig. 2*L*). As these cells usually represent a very minor clone of *WM*^*+*^*T* tumors (*SI Appendix*, Fig. S1*F*), we conclude that extended selection of Myc^high^-alone mammary tumors spontaneously regenerates the Myc^low^/Myc^high^ biclonal phenotype, as the growth of Myc^high^ cells will be hampered until the small Myc^low^ population has sufficiently expanded (Fig. 2*K*, *Right*, and *SI Appendix*, Fig. S2*H*). The failure of 2 out of 4 Myc^high^ tumors to grow attests to the necessity of Myc^low^/Myc^high^ biclonality for tumor growth. No such selection for an unrecombined *R26C*^*LSL-MER*^ allele was observed in biclonal tumors and even after long-term treatment genomic DNA analysis revealed a similar proportion of both alleles in the tumor we analyzed. (Fig. 2 *K*, *Left*, and *L*). This, paired with the observation that Myc^high^ cells have a proliferative advantage in mixed clonal tumors (Fig. 2*H*), implies that admixtures of Myc^low^/Myc^high^ clones converge toward an interdependent equilibrium.

**Fig. 3.**
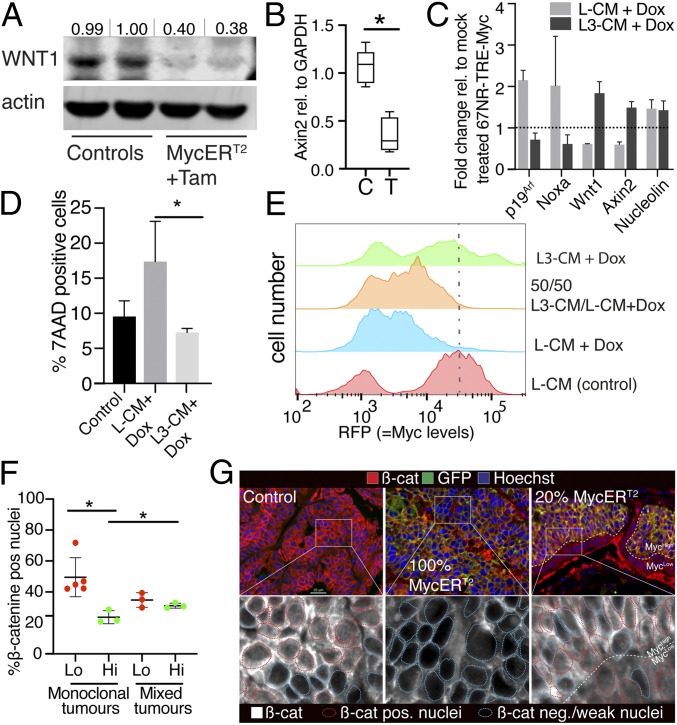
Myc overexpression reduces Wnt expression creating paracrine dependency. (*A*) WB analysis for Wnt1 and actin protein levels in 2 representative *WM*^*+*^*T* and *WMT* control tumors. Bands were quantified by comparison with the loading control and are represented as fold change relative to average of the control tumors. (*B*) qRT-PCR for *Axin 2* in normal mammary glands of *ZP3-Cre;RCAG-MycER*^*T2*^ mice or *ZP3-Cre* control mice (*n* = 5/5). (*C*) Quantitative real-time PCR for indicated targets in cellular extracts from 67NR-Myc-RFP cell lines treated for 24 h with doxycycline and either L-CM or L3-CM, both normalized to respective untreated controls with L-CM alone (*n* = 3). (*D*) Flow-cytometric analysis of cell death in 67NR-Myc-RFP cells after a 24-h treatment with doxycycline and either L-CM or L3-CM, both normalized to respective untreated controls with L-CM alone (*n* = 3). (*E*) Representative flow-cytometric analysis showing a dose-dependent retention of high-RFP/high-Myc–expressing 67NR-Myc-RFP clones during a 72-h treatment of doxycycline and mixtures of L-CM and L3-CM as indicated in the figure. (*F*) Quantification of β-catenin–positive nuclei on *WM*^*+*^*T*, *WM*^*−*^*T* control tumors, and mixed *WM*^*+*^*T/WM*^*−*^*T* tumors stained for β-catenin (*n*: Myc^low^ = 4; Myc^high^ and mixed = 3). (*G*) Immunofluorescence for nuclear β-catenin in *WM*^*+*^*T*, *WM*^*−*^*T* control tumors and mixed *WM*^*+*^*T/WM*^*−*^*T* tumors. For box-and-whisker plots, the error bars represent min to max values, the box represents the interquartile range, and the horizontal line represents the median. *P* values are based on Student’s *t* test: *P < 0.05. For bar charts, error bars represent SD.

Myc-induced apoptosis is mitigated by paracrine survival factors (29). Since suppression of apoptosis in Myc^high^ mammary tumor cells was most evident in areas of interface between Myc^high^ and Myc^low^ cells, we hypothesized that Myc^low^ clones secrete a paracrine survival signal that suppresses Myc^high^ cell apoptosis. One prominent candidate is Wnt itself, which is a potent survival factor in many developing tissues (30) and has been directly shown to block Myc-induced apoptosis (31). Such a prosurvival role for Wnt is especially germane since, in a separate study, we had noted that activation of high levels of Myc antagonizes Wnt signaling. We thus tested whether Wnt and Wnt-signaling was suppressed by Myc in our model system, which was confirmed by the loss of Wnt and Axin2 expression (Fig. 3*A* and *SI Appendix*, Fig. S3 *A* and *B*). *Wnt1* expression in these cells is promiscuously driven from the heterologous *MMTV* promoter; however, the transgene retains almost all of the proximal *Wnt1* promoter and therefore retains many of the original sites for transcriptional activator and inhibitors. To make sure that the effects on Wnt1 were not due to its expression by the MMTV-promoter, we analyzed the expression of endogenous Wnt1 following MycER^T2^ activation in otherwise normal mammary glands. *Wnt1* expression was potently inhibited after only 4 h of tamoxifen administration showing a general negative feedback of Myc signaling on Wnt1 expression (Fig. 3*B*). To test whether these observations were also true in an unrelated breast cancer cell line, we generated 67NR cells that expressed a doxycycline-inducible *TRE-Myc* construct (67NR-Myc-RFP) (*SI Appendix*, Fig. S3*C*). Again, induction of Myc led to an immediate down-regulation of both Wnt1 and Axin2 (Fig. 3*C*, light gray bars). We thus decided to use this cell line to test the hypothesis that Wnt can counteract Myc-mediated apoptosis. We compared the behavior of 67NR-Myc-RFP cells on media conditioned by L-cells expressing recombinant Wnt3a (L3-CM) vs. media conditioned by control L-cells (L-CM). Upon induction of Myc on control media (L-CM), we saw, in addition to the loss of Wnt signaling, robust induction of the p53 stabilizing protein p19^ARF^ and the p53 target gene *Noxa* as well as widespread apoptosis, recapitulating our observations of Myc activation in *WM*^*+*^*T* MycER^T2^ tumors (Fig. 3 *C* and *D*). Conversely, Myc-induced apoptosis of 67NR-Myc-RFP cells was rescued by addition of media conditioned by L-cells expressing recombinant Wnt3a (L3-CM) (Fig. 3*D*). Furthermore, Wnt-mediated rescue of 67NR-Myc-RFP cells on L3-CM correlated with markedly reduced expression of p19^A*RF*^ and Noxa, consistent with general inhibition of p53-dependent Myc induced apoptosis by Wnt signaling (Fig. 3*C*). Hence, cell-free recombinantly expressed Wnt effectively suppresses programmed cell death in mammary tumor cells expressing high levels of Myc.

The maximum level of Myc a cell can tolerate without apoptosis is not an absolute value but highly dependent on cellular context, integrating intracellular stresses with extracellular survival signals. To gain better insight into the levels of Myc tolerated by breast cancer cells over the long term with and without the addition of an exogenous survival signal, we took advantage of the fact that our 67NR-Myc-RFP cell population exhibits a broad range of Myc expression levels because the conditional Myc transgene (*SI Appendix*, Fig. S3*C*) inserts in varied locations and copy numbers in the genomes of the recipient population of 67NR cells. Moreover, the level of Myc in each transfected 67NR-Myc-RFP cell will correlate with that cell’s coinduced RFP fluorescence intensity. Doxycycline-mediated induction of Myc caused quantitative loss of the high-RFP/high-Myc–expressing cells, consistent with the apoptosis that high-Myc expression elicits, while those with lower Myc expression survived and propagated, as evident from the lower overall fluorescence of the outgrowing population (Fig. 3*E*, L-CM [control] vs. L-CM plus Dox). L3-CM Wnt3a-conditioned media mitigated selection against the high-Myc/high-RFP–expressing cells in a dose-dependent manner (50/50 mix of LC-CM/L3-CM vs. L3-CM alone), while L-CM did not (Fig. 3*E*). This is consistent with the notion that Wnt signaling protects breast cancer cells from the apoptotic impact of chronic high Myc activity.

Collectively, these data show that Myc has a negative feedback on Wnt and that, in turn, Wnt signaling can rescue cells from Myc-mediated apoptosis. Having shown a lack of Wnt signaling in *WM*^*+*^*T* tumors (Fig. 3*A* and *SI Appendix*, Fig. S3 *A* and *B*), we set out to analyze the extent of Wnt signaling in the mixed clonal tumors as well. To do so, we used immunofluorescent staining for nuclear β-catenin as a readout of active canonical Wnt signaling. As expected, control (*WM*^*−*^*T* with or without tamoxifen, *WM*^*+*^*T* without tamoxifen) tumors exhibited abundant nuclear β-catenin in most cells, while *WM*^*+*^*T* tumors exposed to tamoxifen did not (Fig. 3 *F* and *G*), presumably due to Wnt1 down-regulation (Fig. 3*A* and *SI Appendix*, Fig. S3*A*). Since *MMTV-Wnt1*–driven tumors are dependent upon Wnt signaling for their maintenance (8), Myc-induced down-regulation of Wnt1 effectively deprives the tumor cells of their own survival signal. By contrast, mixed *WM*^*+*^*T/WM*^*−*^*T* tumors exhibited strong nuclear β-catenin in both Myc^low^ clones and in a high proportion of the Myc^high^ cells lying at the interface of the 2 clones (Fig. 3 *F* and *G*), although this decreased with distance from the boundary with Myc^low^ cells (*SI Appendix*, Fig. S3*E*). Taken together, these results confirm the notion that high levels of Myc deprive tumors of obligate Wnt survival signaling but that this can then be restored by juxtaposition with Myc^low^ cells, so providing mechanistic explanation for the stable mutualism between Myc^high^ and Myc^low^ tumor cells in mammary cancers.

### Executable Modeling Identifies Pharmacological Vulnerabilities in Heterogenous Myc Mammary Tumors.

**Fig. 4.**
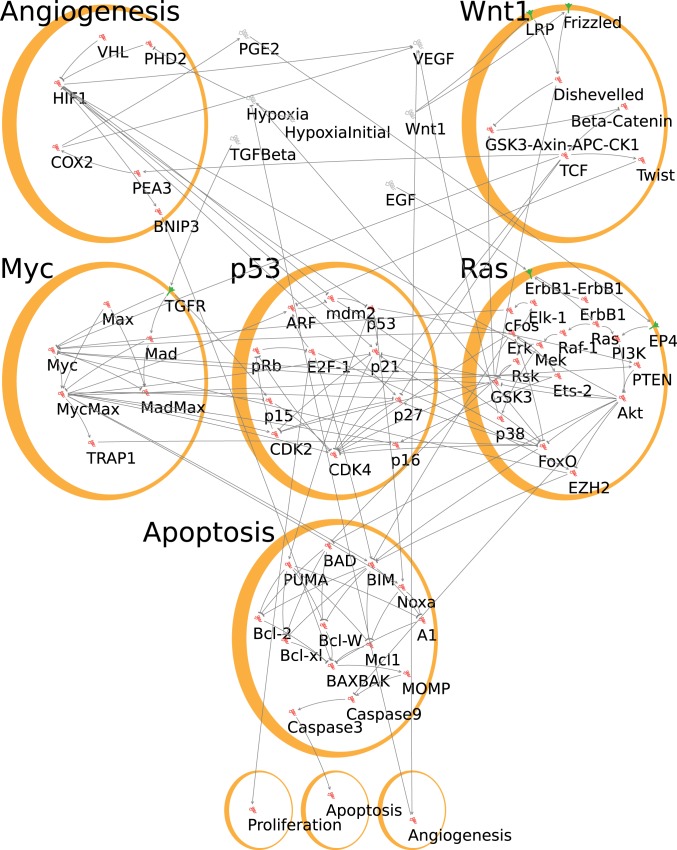
The executable network model as seen in the BMA tool. The nodes representing genes and proteins are grouped by pathway for ease of interpreting the diagram, while each phenotype is singled out in its own module. Nodes outside any module represent external factors produced by or influencing cell behavior. The arrows represent activating interactions, while the bars represent inhibition.

While it is possible that the obligate mutualism between Myc^low^ and Myc^high^ mammary tumor cells create novel vulnerabilities for therapeutic targeting, the complexity, redundancy, and feedback in biological networks make the search for such contextual vulnerabilities both difficult and arduously empirical. We therefore turned to executable mechanistic *in silico* models, which allow for rapid, systematic simulation and testing of large numbers of signaling network perturbations. We constructed an initial executable model of breast cancer using publicly available data drawn from the literature (Datasets S1 and S2). The network is modeled as a qualitative network (32), which is then simulated and analyzed with the BioModelAnalyzer (BMA) tool (33) (http://biomodelanalyzer.org/). The process of building and testing this network model is illustrated in *SI Appendix*, Fig. S4, and the result is an executable network encompassing proteins and transcription factors that contribute to the overall tumor cell phenotype (Fig. 4). Although not an exhaustive map of all interactions within a cell, the model nonetheless models the key pathways in our system and the fidelity of its iterations may then be rapidly evaluated by in vitro and in vivo experiment. To address Wnt-driven triple-negative breast cancer specifically, we focused on the Wnt1 and EGF receptor pathways, since these are 2 predominant drivers of oncogenic signaling in ER- and HER2-negative breast cancers that converge downstream on Myc and Ras effector pathways. In addition, since aberrant cross talk and excessive signaling flux across Myc and Ras pathways trigger tumor suppression, we included the p53 signaling pathway in our executable model. Finally, to encompass critical aspects of the interaction between tumor clones and their microenvironment, we simulated responses to hypoxia via the HIF1α pathway and consequent release of signaling molecules such as VEGF. The overall output of the model governs the net balance between cell proliferation and apoptosis.

The genes, proteins, and environmental conditions of a cell in the tumor are each represented by nodes in the executable network model (Fig. 4). Their behaviors are defined by target functions attached to each node. These target functions are mathematical formulae that define how a protein responds to changes in the other proteins with which it interacts. Target functions can therefore model, for example, a series of proteins activated in a signaling cascade, or their change in expression in response to a transcription factor. Mutations, drug treatments, and environmental conditions can be represented in the network model by changing the target functions of nodes. This allows the network model to reproduce the different cells in our mouse model: For example, Myc^high^ conditions were reproduced by fixing the activity of Myc to be a constant value at an arbitrarily maximum level (Dataset S5).

We first verified the behavior of the executable model against published data derived from experiments on breast cell lines with known oncogenic mutations. These were represented in the model by changes to the relevant target functions of the nodes representing the affected genes and proteins, as described above. We then adjusted the levels of various nodes to represent experimental perturbations: For example, fixing a node at zero represents pharmacological inhibition. The resulting behavior of the model is then compared with that observed by experiment (Dataset S3). In this way, we can test whether the model’s behavior is correct under a wide array of perturbations. We further tested the model against each of the monoclonal *MMTV-Wnt1* Myc^low^ and Myc^high^ tumors by comparing the predicted activity of nodes in the model with the activity observed experimentally (Dataset S4). We iteratively alternated between testing and refining the model until all of the simulation results reproduced the experimental observations (*SI Appendix*, Fig. S4). Comparisons were made predominantly against in vitro published experiments, so angiogenesis was not simulated. However, when modeling the in vivo tumors, we introduced an angiogenic node. Since the in vivo tumors were not HER2 driven, these nodes were removed.

We next simulated the effect of treatment on the mixed Myc^high^ and Myc^low^ tumors, including the predicted cross talk between the clones, to generate cell fate predictions and to find the most effective targeted therapies for each clone. As the cooperation of clones was mediated by changes in the microenvironment, we were able to simulate each clone in the mixed tumor separately by modifications to the relevant node level to represent these changes; for example, increasing the activity of the Wnt1 node to represent that there is a source of paracrine Wnt1 for the Myc^high^ cells from the Myc^low^ cells in the mixed tumors. This was in addition to the changes representing the mutations in each subclone. These node level changes are depicted in Dataset S5. We modeled the effect of targeted therapies by setting the activity of a node to zero, representing inhibition by a drug, and repeated this for all major nodes. This allowed us to model the therapeutic outcome (net proliferation or net apoptosis) of modalities that target one clone or the other.

**Fig. 5.**
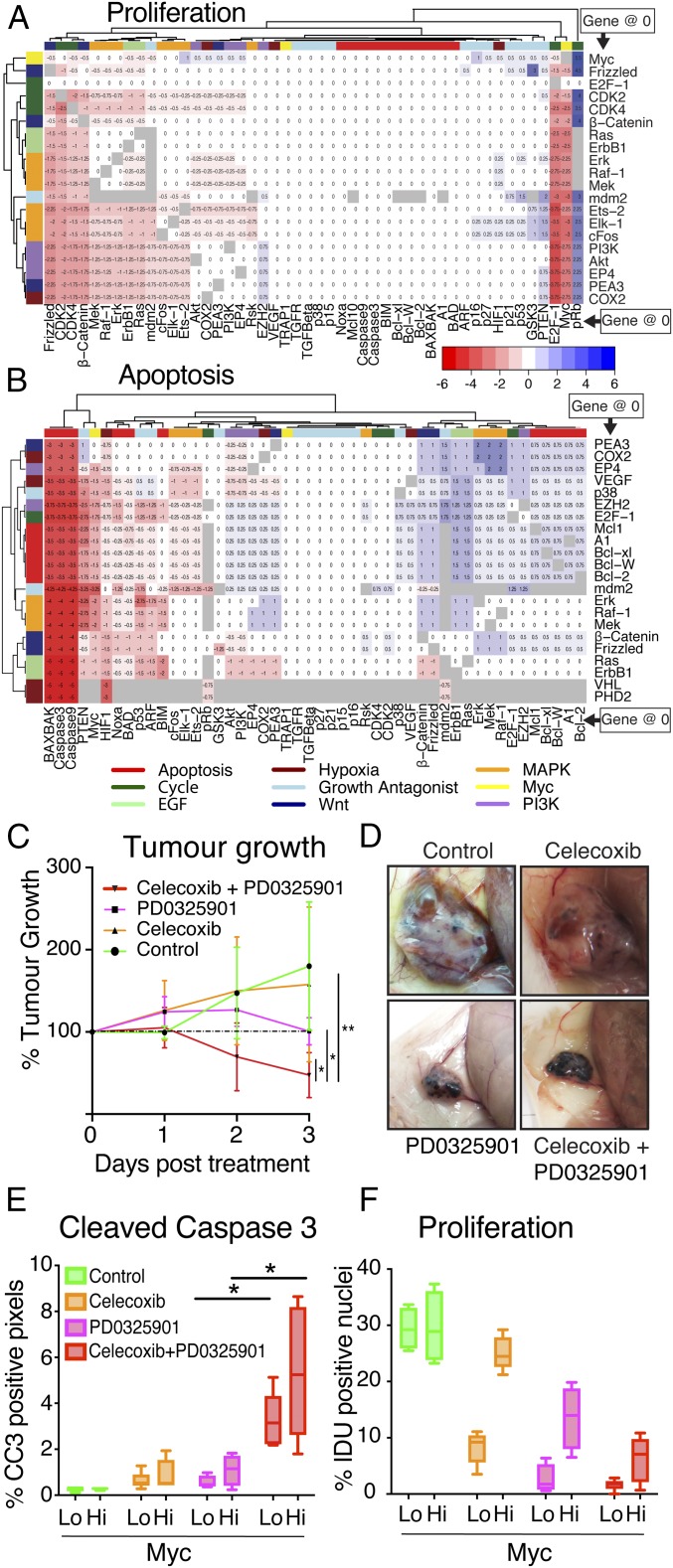
Change in effect of therapy when adding a second inhibitor predicted by the computational model, and successful MEK and COX2 inhibition combination therapy in vivo. (*A* and *B*) Rows and columns of heatmaps are colored by pathway to which nodes which are treated belong; the pathway categories are laid out in Dataset S7. Mean change in proliferation (*A*) or apoptosis (*B*) across both clones when adding a second inhibitor to an inhibitor already shown to be effective in monotherapy. In the heatmap, the *y* axis shows first inhibitor; *x* axis, the inhibitor that is added in combination. The gray boxes are combinations that are nonsensical (2 different inhibitions of the same node) or cause apoptosis above 3 in the healthy cells. Inhibition of PHD2 or VHL cause high apoptosis with every partner other than each other and are removed as this otherwise prevents clustering of the heatmaps. (*C*) Caliperimetric measurement of tumor growth of mixed *WM*^*+*^*T/WM*^*−*^*T* tumors during 3 d of treatment with tamoxifen (100 μg/mouse, twice daily) followed by 4-d treatment with tamoxifen and drug combinations (celecoxib, 20 mg/kg/d; PD0325901, 10 mg/kg/d). Measurements are normalized to the beginning of the drug treatment course as tumors at this stage were at different sizes (*n* = 4 to 5; error bars represent SD). (*D*) Representative picture of the gross morphology of mixed *WM*^*+*^*T/WM*^*−*^*T* tumors after the treatment described above. (*E*) Quantification of cell death in mixed *WM*^*+*^*T/WM*^*−*^*T* tumors as percentage of CC3-positive pixels in the individual clones (*n*: control, PD0325901 = 4; celecoxib, celecoxib plus PD0325901 = 5). (*F*) Quantification of proliferation in mixed *WM*^*+*^*T/WM*^*−*^*T* tumors by automated counting of IDU-positive nuclei over total nuclei in the individual clones. (*n*: control, PD0325901 = 4; celecoxib, celecoxib plus PD0325901 = 5). For box-and-whisker plots, the error bars represent min to max values, the box represents the interquartile range, and the horizontal line represents the median. *P* values are based on Student’s *t* test: *P < 0.05.

The model predicted that heterogenous tumors would be more resilient to therapy, with higher proliferation and lower apoptosis than pure Myc^high^ or Myc^low^ clones for the same inhibiting drug (*SI Appendix*, Fig. S5 *A* and *B*). This is consistent with our experimental evidence of mutual benefit for each clone from one another. The model also predicted that most inhibitors would be more effective against one clone than another, with Myc^high^ cells being resistant to cell cycle arrest but more vulnerable to apoptosis (*SI Appendix*, Fig. S5 *A* and *B*), which meant that targets in some pathways were predicted to be effective for one clone but not the other. Because of these differences in the effectiveness of a single inhibitor in treating either of the 2 different clones, as well as the proclivity of neoplastic systems to acquire compensatory or evolutionary resistance to monotherapies, we hypothesized that simultaneous application of 2 inhibitors would be therapeutically more effective. Accordingly, we simulated pairwise combinations of inhibitors across all major nodes (*SI Appendix*, Figs. S6 *A*–*E* and S7 *A*–*E*). This generated a striking increase in the proportion of modeled therapies predicted to be successful, many eliciting marked impacts on both Myc^high^ plus Myc^low^ tumor cell populations (*SI Appendix*, Fig. S8 *A* and *B*). We then further filtered our search on the basis of target druggability and searched for combinatorial synergy by assessing whether the efficacy of one inhibitor was enhanced by addition of a second inhibitor (Fig. 5 *A* and *B* and *SI Appendix*, Fig. S9).

From this analysis, the combination of COX2 and MEK inhibition appeared to be the most effective combination for increasing apoptosis (Fig. 5*B* and *SI Appendix*, Fig. S9*B*). The model predicted that inhibition of MEK alone would induce more apoptosis in the Myc^high^ than the Myc^low^ cells, and that in the Myc^low^ cells, inhibition of MEK and COX2 together would improve the cytotoxic effect over either inhibition of MEK or COX2 applied separately, while still remaining effective against Myc^high^, and so effectively treat both populations of cells.

To test the predicted therapeutic efficacy of this combination in vivo, we again used our biclonal Myc^high^/Myc^low^ tumor model: High MycER^T2^ was activated in the Myc^high^ subpopulation, and 48 h later, mice were treated with either the COX2 inhibitor celecoxib or the MEK inhibitor PD0325901 alone, or with the 2 inhibitors combined. The tumors were then observed over a further 3 d. Each inhibitor alone offered some therapeutic benefit: COX2 inhibition slowed down overall tumor growth, while MEK inhibition stalled net tumor expansion (Fig. 5*C*). However, celecoxib and PD0325901 in combination induced rapid tumor regression (Fig. 5*C*), resulting in residual masses almost devoid of tumor cells and comprising mainly hemorrhagic cysts (Fig. 5*D* and *SI Appendix*, Fig. S10 *A* and *B*). Detailed histological analysis of the few remaining regions harboring residual tumor cells showed a significant increase in apoptosis, most evident in the Myc^high^ cells, together with a decrease in proliferation, most notably in the Myc^low^ clones (Fig. 5*F* and *SI Appendix*, Fig. S10 *C*–*F*). These single and combined responses were consistent with our executable model’s predictions (Fig. 5 *E* and *F*). Last, we decided to test whether combining the more recently developed Cox/Lox inhibitor licofelone with PD0325901 gave any advantage over the single target drug celecoxib. This was not the case, as the response of the tumor to the triple inhibition was indistinguishable to the double inhibition (*SI Appendix*, Fig. S10 *A*–*F*). This result is consistent with a further test of the specific Lox inhibitor zileuton, which did not significantly synergize with MEK inhibition (*SI Appendix*, Fig. S10 *A*–*F*). Due to the relatively high doses of licofelone used (100 mg/kg/d) to observe a combined effect with MEK inhibition compared to those used for celecoxib (20 mg/kg/d) and given that celecoxib has fewer known side effects in humans, the dual-inhibition with PD0325901 and celecoxib seems preferable over licofelone.

Taken together, these results show how computational modeling of solid biclonal tumors allowed us to devise a very potent therapeutic strategy.

## Discussion

The component tumor cells of many human breast cancers exhibit persistent heterogeneity in Myc expression (12, 13, 16). Individually, ubiquitously Myc^high^ or Myc^low^ mammary tumor cells exhibit markedly different features, each of which significantly restrains tumorigenic potential. Mammary tumor cells with high levels of Myc enjoy significant potential growth advantages by virtue of their enhanced proliferative rates, invasiveness, and their capacity to instruct an angiogenic microenvironment conducive to tumor spread. However, elevated Myc predisposes Myc^high^ cells to apoptosis. Consequently, Myc^high^ cells are handicapped by a greatly increased reliance on continuous survival signals (29). Since elevated Myc also concomitantly suppresses expression of Wnt1, a key autocrine survival factor for mammary epithelial cells, tumors comprising only Myc^high^ tumor cells effectively starve themselves of autocrine survival signals. Conversely, tumor monocultures of Myc^low^ cells, while enjoying an intrinsically much lower predisposition to apoptosis, are constrained by their inability to instruct significant stromal angiogenic changes, limiting them to indolent, hypovascular, hypoxic, and necrotic lesions. The stability of Myc^high^/Myc^low^ mixtures of tumor cells therefore appears to derive from the obligate complementarity of their 2, individually self-limiting, biologies. While proximity of invasive and angiogenic Myc^high^ cells facilitates both growth and spread of Myc^low^ cells, reciprocal proximity of Myc^low^ cells provides sufficient Wnt1 to keep their more aggressive siblings alive. This relationship becomes particularly clear when trying to grow tumors solely comprising Myc^high^ cells. The observed escapee tumors convert to a heterogeneous phenotype through outgrowth of a minor MycER^T2^-negative clone (Fig. 2 *J* and *K*). The observation that more than one-half of the cells comprising the escapee tumor we analyzed genomically did not express MycER^T2^ (Fig. 2*L*) suggests the need for a significant amount of Myc^low^ cells to support Myc^high^ cells, and is consistent with the idea that Wnt, a heavily palmitoylated and glycosylated ligand, acts at relatively short range (34, 35). Therefore, sufficient Wnt is a prerequisite for any secondary consequences of polyclonality, such as the development of tumor supportive stroma by Myc^high^ clones (Figs. 1 *D* and *E* and 2 *E* and *F* and *SI Appendix*, Figs. S1*K* and S2*E*). Such mutualism explains why, in human Myc^high^/Myc^low^ mixed mammary tumors, Myc^high^ clones typically do not rapidly overgrow Myc^low^ clones (13). A similar role for Wnt-secreting supportive niches in tumor evolution and maintenance has recently been identified in lung adenocarcinomas (7), indicating that such mutualism may be a common feature of tumor cells expressing high levels of Myc. Furthermore, when we switched on MycER^T2^ in an untransformed mammary gland, we equally observed rapid loss of Wnt1 and inhibition of Wnt signaling (Fig. 3*B*). This indicated that the mutual exclusivity between expression of high levels of Myc and Wnt ligands is not idiosyncratic for tumors, but rather a general phenomenon. This is most likely part of an inherent tissue organization, where proliferative niches are organized in proliferative (Myc^high^/Wnt^low^) cells and supportive (Myc^low^/Wnt^high^) cells. Myc-heterogenous mammary tumors appear to retain this reliance on supportive niches and evolve accordingly.

There is clearly a complex interplay between the key growth and survival factors, Myc and Wnt, that is highly contextual. For example, Myc is reported to down-regulate the secreted Wnt inhibitors DKK1 and SFRP1 (36), implying that Myc acts to sensitize cells to Wnt signaling. Myc^high^ cells are thus dependent on Wnt signaling but create a more permissive environment for this very signaling to occur. We noted a similar complexity in the interplay between Myc and Wnt on the level of the apoptotic machinery. We observed a clear quenching of the p19^Arf^→p53 axis in Myc-expressing cells when treated with Wnt3a conditioned media (L3-CM) but recorded an unexpected up-regulation of PUMA. However, this was inconsequential in terms of inducing apoptosis (Fig. 3*D* and *SI Appendix*, Fig. S3*D*). This shows that the downstream signaling of large signaling hubs such as Wnt and Myc is highly contextual, as is the resulting phenotypic outcome, such as cell death or proliferation.

In both *Drosophila* and mouse development, cells expressing high levels of Myc are reported to exhibit supercompetition—they not only outgrow their Myc^low^ neighbors but also actively eliminate them. In the case of mixed Myc^high^/Myc^low^ mammary tumors, such aggressive supercompetition would, of course, be expected to expeditiously eradicate the Wnt1-producing Myc^low^ cells that are the principal source of Wnt1 survival signals keeping the Myc^high^ cells alive. However, we see no evidence of such supercompetition in our mixed Myc^high^/Myc^low^ mammary tumors: Rather, the principal determinant of Myc^high^ cell fate appears not to be their innate competitiveness but their increased dependency on Myc^low^-generated survival signals. It may be that reported instances of supercompetition in mice and *Drosophila* development arise in situations where the proclivity of Myc^high^ cells to undergo apoptosis is abrogated, for example by abundant survival factors. Likewise, it is possible that Myc-driven supercompetition is a significant factor in evolution of tumors in which Myc-induced apoptosis is circumvented by secondary, antiapoptotic mutation.

The notion that certain aspects of the oncogenic process might expose novel vulnerabilities in tumor cells underpins the rationale for selective cancer therapies, and the obligate mutualism we observe between Myc^high^ and Myc^low^ mammary cancer cells is one such example. To explore this case, we generated a computational model of the Myc/Ras/p53 signaling network in breast cancer cells. Starting from a general executable model of breast cancer, we added Wnt1 as a constant node and overlaid high Myc activity. Our initial simulation predicted levels of proliferation higher than those seen in Myc high tumors in vivo, suggesting that some level of interruption in Wnt signaling was at play in the tumors. This was experimentally confirmed and shown to be due to Myc-dependent suppression of Wnt expression. This was then factored back in to the computational model of both Myc^high^-only tumors and Myc^high^/Myc^low^ heterogenous tumors, to accurately predict the underlying set of mechanistic rules that indicated biclonal mutualism of the Myc^high^/Myc^low^ mixed tumors.

A key dividend of such executable models is their ability to screen vast numbers of therapeutic combinations virtually and identify combinatorial regimens that specifically target the obligate biclonality of the tumors. Thus, the model predicted that coinhibition of MEK and COX2 would exert a more potent therapeutic impact on both clonatypes than their individual inhibition would on either individual clonatype. The model correctly predicted the augmented response of the individual clones to various inhibitor combinations, including the sharp drop in proliferation of Myc^low^ cells when exposed either to MEK inhibition alone or to coinhibition of MEK and COX2 together, and the strong apoptotic response of Myc^high^ clones exposed to the combination therapy. Intriguingly, our model consistently underestimated the efficacy of the inhibitors, particularly with respect to their impact on the Myc^high^ tumor cell population. However, in its current form, the model considers only initial clonal distributions and does not accommodate clonal dynamics known to occur during the course of treatment. This is a drawback, since we predict that the expected loss of Myc^low^ cells during treatment will progressively curtail the survival of Myc^high^ clones due to loss of Wnt1 signaling. Future development of the model could be extended to accommodate the shifting interactions that follow from changes in clonal composition of the tumor during treatment. A further benefit of our executable modeling approach is that it suggests potential mechanisms by which the combination treatment works. Specifically, it suggests that therapeutic efficacy relies on disrupting the balance between proapoptotic and antiapoptotic signals: MEK inhibition blocks antiapoptotic signaling, and so predominantly affects the Myc^high^ clone, whereas COX2 inhibition increases proapoptotic signaling, thereby reinforcing the impact of MEK inhibition on the Myc^low^ cells.

As our understanding of normal tissue organization and its pathogenic equivalent in tumors deepens, we propose that qualitative computational models such as the one we have used in this study will be needed to grasp the totality of iterative and dynamic tumor cell signaling—both in its initial state and as it morphs and adapts to perturbations induced by treatments and spontaneous changes in the genome and epigenome. Only in this way can we stay ahead of drug resistance and disease relapse.

## Materials and Methods

### Mice and In Vivo Procedures.

All treatments and procedures of mice were conducted in accordance with protocols approved by the by Home Office UK guidelines under project licenses to G.I.E. (70/7586, 80/2396) at the University of Cambridge. The following mouse strains were used: *Rosa26-CAG-lox-STOP-lox-MycER*^*T2*^/*Rosa26-mTmG/MMTV-Wnt1* and *Rosa26-CAG-lox-STOP-lox-MycER*^*T2*^. MycER^T2^ was activated by administration of tamoxifen at 1 mg/20 g, i.p., twice daily, id administration period <12 h. IDU was administered at 1 mg/20 g. More details are in *SI Appendix*.

### IHC and Immunofluorescence.

Standard protocols were followed for IHC and immunofluorescence. For details, see *SI Appendix*. The following primary antibodies were used: HIF1α (sc-10790; 1:50); CD31 (ab28364; 1:100); cleaved caspase 3 (cs9664; 1:1,000); estrogen receptor α (sc-542; 1:50); IDU (BD347580; 1:100); p19^*ARF*^ (sc-32748; 1:100); β-catenin (BD610153; 1:250); Myc (ab32072; 1:1,000); GFP (ab6556; 1:500); and p53 (Leica CM5p; 1:500). Unless otherwise stated, quantifications were carried out for at least 3 visual field on at least 3 independent biological replicates.

### Quantitative Real-Time PCR.

SBYR Green Master Mix (Thermo Fisher Scientific)-based qRT-PCR was performed after RNA extraction and complementary DNA production following standard protocols. For primer, see *SI Appendix*.

### Genomic DNA Analysis.

Genomic DNA was extracted using The PureLink Genomic DNA Mini Kit (Invitrogen), following the manufacturer’s instructions. Genomic DNA from samples on long-term tamoxifen studies were extracted by the same method from shavings of formalin-fixed freeze-preserved tissues. The presence of cre recombination at the R26C^MER^ allele was tested via quantitative digital droplet PCR on the QX 200 droplet reader (Bio-Rad), following the manufacturer’s standard protocol. For primers and probes, see *SI Appendix*.

### Western Blotting.

Samples were prepared using standard protocols. Proteins were labeled following the manufacturer’s protocol for the Li-Cor Near-Infrared (NIR) Western Blot Detection system, or the Amersham 600 Imager. The following primary antibodies were used: p53 (Leica; NCL-L-p53-CM5p; 1:2,000); actin (Santa Cruz; sc-69879; 1:5,000); Wnt1 (Abcam; ab15251; 1:1,000); CC3 (Cell Signaling Technologies; 1:1,000); Myc (ab32072; 1:1,000); secondary antibody: goat anti-rabbit (sc-2301; 1:7,500).

### Therapeutic Studies.

Tumors were generated as described above at ratios of Myc^high^/Myc^low^ clones of 30%:70%. Tamoxifen treatment started at a size of ∼0.5 cm^3^, and from day 3 drugs were administered via oral gavages. Tumors were measured daily, and IDU was injected 2 h prior to culling. For details on drug administration, see *SI Appendix*.

### Using Qualitative Networks to Model Genetic and Molecular Networks.

We model the system as a qualitative network (32), an extension of the Boolean network formalism, using the BMA tool (*SI Appendix*, *Methods*) (33) (http://biomodelanalyzer.org/).

The network (Fig. 4) consists of nodes representing the decision-making machinery of the cell; the genes, proteins, and transcription factors, e.g., Myc, and those representing the overall cell phenotype; proliferation and apoptosis. Corresponding genes for each node are given in Dataset S6. The interactions between these components are represented by edges, in the network model.

Each node has an associated target function, which determines the value it takes, based on the values of nodes that connect to it (Dataset S2). These connections, and the target functions that describe their interactions, are drawn from the literature (Dataset S1) of experiments on breast cancer cell lines.

Most nodes use the default BMA target function, avg(pos)−avg(neg), which compares the average state of all of the nodes that connect to the current node via an activating, i.e., positive edge, to the average state of all of the nodes that connect via an inhibitory, i.e., negative edge. In cases where there were no positive inputs, we assumed some constitutive activity of the node by setting the target value to be some constant value minus the average negative inputs. More complex target functions were used when the literature evidence around the interaction demanded it (Dataset S2).

The model was built and tested using literature data, and literature supporting the network is collated in Dataset S1, target functions in Dataset S2, and experiments used to test the model in Datasets S3 and S4, with more details about this process in *SI Appendix*, *Methods*. The process of building and testing the network model is illustrated in *SI Appendix*, Fig. S4. We use BMA to predict cell behavior by searching for stable states, or attractors. These capabilities can be accessed through the BMA GUI at http://biomodelanalyzer.org or through the command line with a local instance https://github.com/Microsoft/BioModelAnalyzer.

### Exhaustive Computational Search for Effective Drug Inhibitor Combinations.

To assess efficacious combinations of targeted therapies, we performed single and pairwise inhibition (set target function to zero) of every single node or pair of nodes in the network to represent the effects of drug inhibition, in addition to the background of existing mutations shown in Dataset S5. These perturbations are shown in the axes of the heatmaps in *SI Appendix*, Figs. S5 *A* and *B*, S6, and S7. Ranking our various potential targets for perturbation by the number of available inhibitors according to the Drug–Gene Interaction Database (37) accessed using the package rDGIdb (38) (*SI Appendix*, Fig. S9*C*), we considered only those nodes for which there was at least one drug known to interact with it. For each perturbation, we find the stable state of the network, or the smallest range to which we can constrain each node. In order to compare different tumor clones, we also added a background set of mutations that are unaffected by the above mutations. If BMA could not restrict the value of the node to a single value, for example if there is an oscillation between 2 levels of activity, we used the mean of the minimum and maximum value. Visualization for Fig. 5 *A* and *B* and *SI Appendix*, Figs. S5–S9, was performed using R (39) and ggplot2 (40). The BMA network model (JSON file) can be found at http://www3.bioc.cam.ac.uk/fisher/.

## Supplementary Material

Supplementary File

Supplementary File

Supplementary File

Supplementary File

Supplementary File

Supplementary File

Supplementary File

Supplementary File
