# Supplementary material for: Heterogeneity of Myc expression in breast cancer exposes pharmacological vulnerabilities revealed through executable mechanistic modeling
Source: Proc Natl Acad Sci U S A. 2019 Oct 14;116(44):22399–408. doi: 10.1073/pnas.1903485116 (PMC6825310; doi:10.1073/pnas.1903485116)
Supplement: Supplementary File [file pnas.1903485116.sapp.pdf]

Supplementary Information for

Heterogeneity of Myc Expression in Breast Cancer Exposes Pharmacological Vulnerabilities Revealed through Executable Mechanistic Modeling

Peter Kreuzaler[a,b,1], Matthew A. Clarke[a,1], Elizabeth J. Brown[a], Catherine H. Wilson[a], Roderik M. Kortlever[a], Nir Piterman[c], Trevor Littlewood[a], Gerard I. Evan[a,2] and Jasmin Fisher[a,d,2]
[a]Department of Biochemistry, University of Cambridge, Cambridge CB2 1GA, United Kingdom; [b]Oncogenes and Tumour Metabolism Lab, The Francis Crick Institute, London NW1 1AT, United Kingdom; [c]Department of Computer Science and Engineering, University of Gothenburg, SE-41296, Sweden; [d] UCL Cancer Institute, University College London, WC1E 6DD, United Kingdom

[1] These authors contributed equally to this work
[2] Correspondence should be addressed to Jasmin Fisher jf416@cam.ac.uk or Gerard Evan gie20@cam.ac.uk

This PDF file includes:

Figs. S1 to S10
Captions for datasets S1 to S10
Supplementary Methods
References for SI reference citations

Other supplementary materials for this manuscript include the following:

Datasets (excel files) S1 to S7

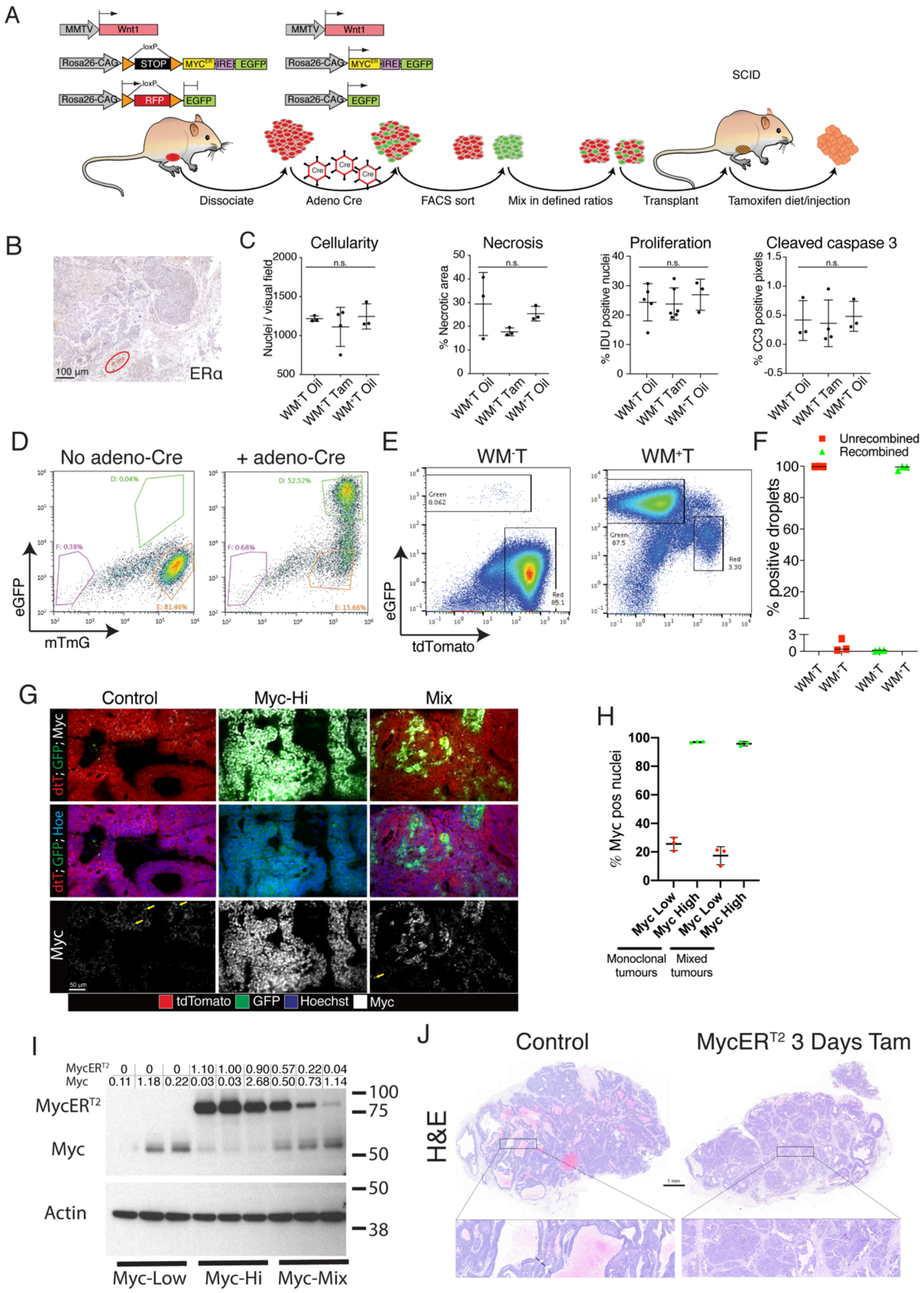
A
MMTV
Wnt1
Rosa26-CAG
loxP
STOP
IRES
EGFP
RFP
SCID
Cre
Dissociate
Adeno Cre
FACS sort
Mix in defined ratios
Transplant
Tamoxifen diet/injection
B
100 µm
ERα
C
Cellularity
Necrosis
Proliferation
Cleaved caspase 3
Nuclei / visual field
% Necrotic area
% IDU positive nuclei
% CC3 positive pixels
n.s.
D
No adeno-Cre
+ adeno-Cre
eGFP
mTmG
E
tdTomato
F
Unrecombined
Recombined
% positive droplets
G
Control
Myc-Hi
Mix
dtT; GFP; Myc
dtT; GFP; Hoe
Myc
tdTomato
GFP
Hoechst
Myc
H
% Myc pos nuclei
Myc Low
Myc High
Monoclonal tumours
Mixed tumours
I
MycER
Myc
Actin
Myc-Low
Myc-Hi
Myc-Mix
J
Control
MycER 3 Days Tam
H&E

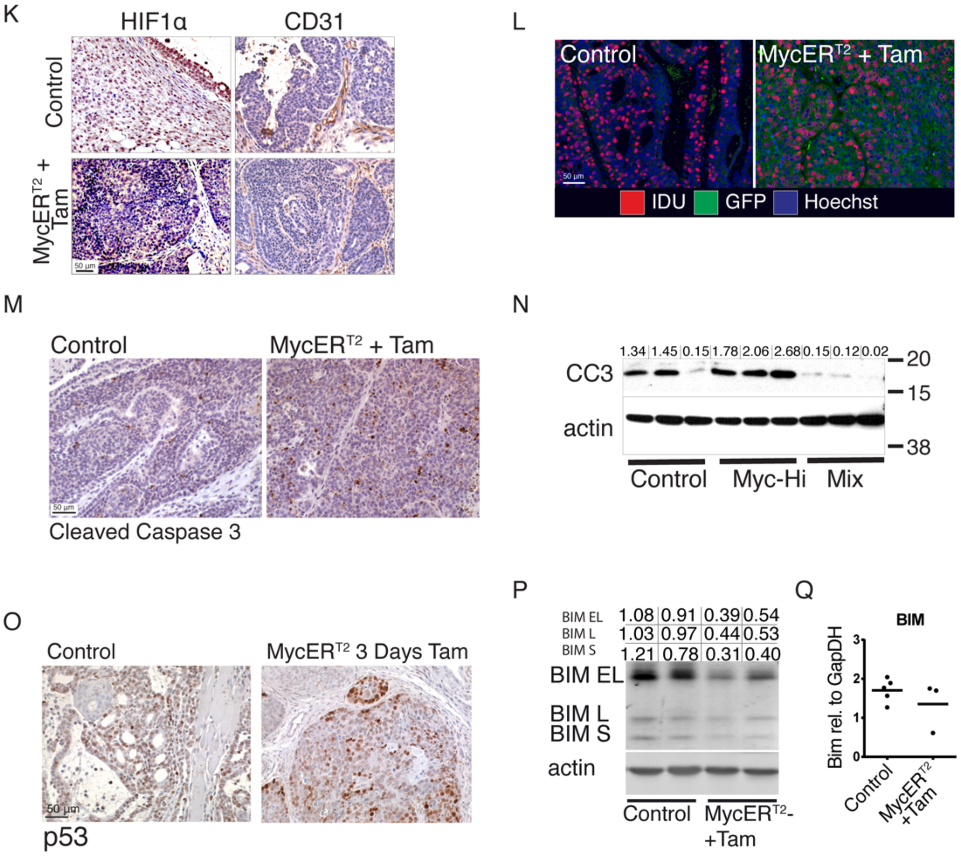
K
HIF1α
CD31
Control
MycER T2 + Tam
50 µm
L
Control
MycER T2 + Tam
50 µm
IDU
GFP
Hoechst
M
Control
MycER T2 + Tam
50 µm
Cleaved Caspase 3
N
1.34 1.45 0.15 1.78 2.06 2.68 0.15 0.12 0.02
CC3
actin
20
15
38
Control
Myc-Hi
Mix
O
Control
MycER T2 3 Days Tam
50 µm
p53
P
BIM EL 1.08 0.91 0.39 0.54
BIM L 1.03 0.97 0.44 0.53
BIM S 1.21 0.78 0.31 0.40
BIM EL
BIM L
BIM S
actin
Control
MycER T2- +Tam
Q
BIM
Bim rel. to GapDH
0
1
2
3
Control
MycER T2 +Tam

Figure S1. Creation and validation of traceable *in vivo* model of *MMTV-Wnt1*-driven chimeric mammary tumour comprising an admixture of low Myc and reversibly switchable high Myc-expressing clones. Related to Figure 1.

a) Graphical representation of the genetic background of the mice carrying the *MMTV-Wnt* allele*,* the *Rosa26-CAG-lox-stop-lox-Myc*^{ER}*-IRES-EGFP* allele and the *Rosa26-mTmG* allele pre and post Adeno-Cre mediated recombination, as well as the workflow to generate mono- clonal and mixed *WM*$^{+}$*T/ WM*$^{-}$*T* tumours.
b) Representative image showing the overall ER-negativity in post Tamoxifen pre-treatment. The small ER-positive patch in the bottom left (circle) is a rare occurrence of ER positive cells stemming from host-derived inclusions and was included to proof functionality of the antibody.
c) Controls derived from Fig. 1 c, d and f, split by respective control treatments show no overall trend with and without Tamoxifen proving Tamoxifen irresponsiveness, in line with the ER- negativity (error bars represent standard deviation, horizontal line the mean, dots represent individual mice).
d) Representative FACS plots showing cells with and without recombination and the respective gating. Note that EGFP positive cells retain dtTomato expression at this stage, due to the long half-life of the protein.
e) Representative flow cytometric analysis of *WM*$^{-}$*T* and *WM*$^{+}$*T* tumours after three days Tamoxifen treatment (n=3)
f) Digital Droplet PCR testing for R26C^{MER} on genomic DNA of *WM*$^{-}$*T* and *WM*$^{+}$*T* tumours after three days of Tamoxifen treatment (n=3).
g) Immunofluorescent for Myc expression on frozen tissue sections of *WM*$^{+}$*T*, mixed *WM*$^{+}$*T/ WM*$^{-}$*T* and *WM*$^{-}$*T* control tumours (n=3).
h) Quantification of g)
i) Western blot analysis of Myc in *WM*$^{+}$*T,* mixed *WM*$^{+}$*T/ WM*$^{-}$*T* and *WM*$^{-}$*T* control tumours. Bands were quantified with respect to the loading control and represent a fold change compared to average of the control tumours.
j) Haematoxylin & eosin staining of representative sections of *WM*$^{+}$*T* tumours and controls at low and high (inset) magnification.
k) Representative images of immunohistochemical DAB staining of *WM*$^{+}$*T* tumours and controls for CD31 and Hif1α.
l) Representative images of immunofluorescent staining for 5-Iodo-2′-deoxyuridine (IDU, red), EGFP (green) and DNA (Hoechst - blue), of WM+T tumours and controls. IDU incorporation indicates cells in S-phase.
m) Representative images of immunohistochemical DAB staining of WM^{+}T tumours and controls for cleaved Caspase 3.
n) Western Blot analysis of *WM*$^{+}$*T,* mixed *WM*$^{+}$*T/ WM*$^{-}$*T* and *WM*$^{-}$*T* control tumours for cleaved caspase 3 (CC3) as a readout for the occurrence of cell death. Note that both necrotic as well as apoptotic tissues lead to accumulation of CC3. Bands were quantified with respect to the loading control and represent a fold change compared to average of the control tumours.
o) Representative images of immunohistochemical DAB staining of *WM*$^{+}$*T* tumours and *WM*$^{-}$*T* controls for p53.
p) Western Blot analysis of a representative duplicate of *WM*$^{+}$*T,* and control tumours for the BH3 only protein Bim, showing lack of Bim induction by Myc. Bands were quantified with respect to the loading control and represent a fold change compared to average of the control tumours.
q) RT-PCR of *WM*$^{+}$*T,* and control tumours for *Bim*, showing a lack of transcriptional engagement by Myc (horizontal line represents the mean, n=3).

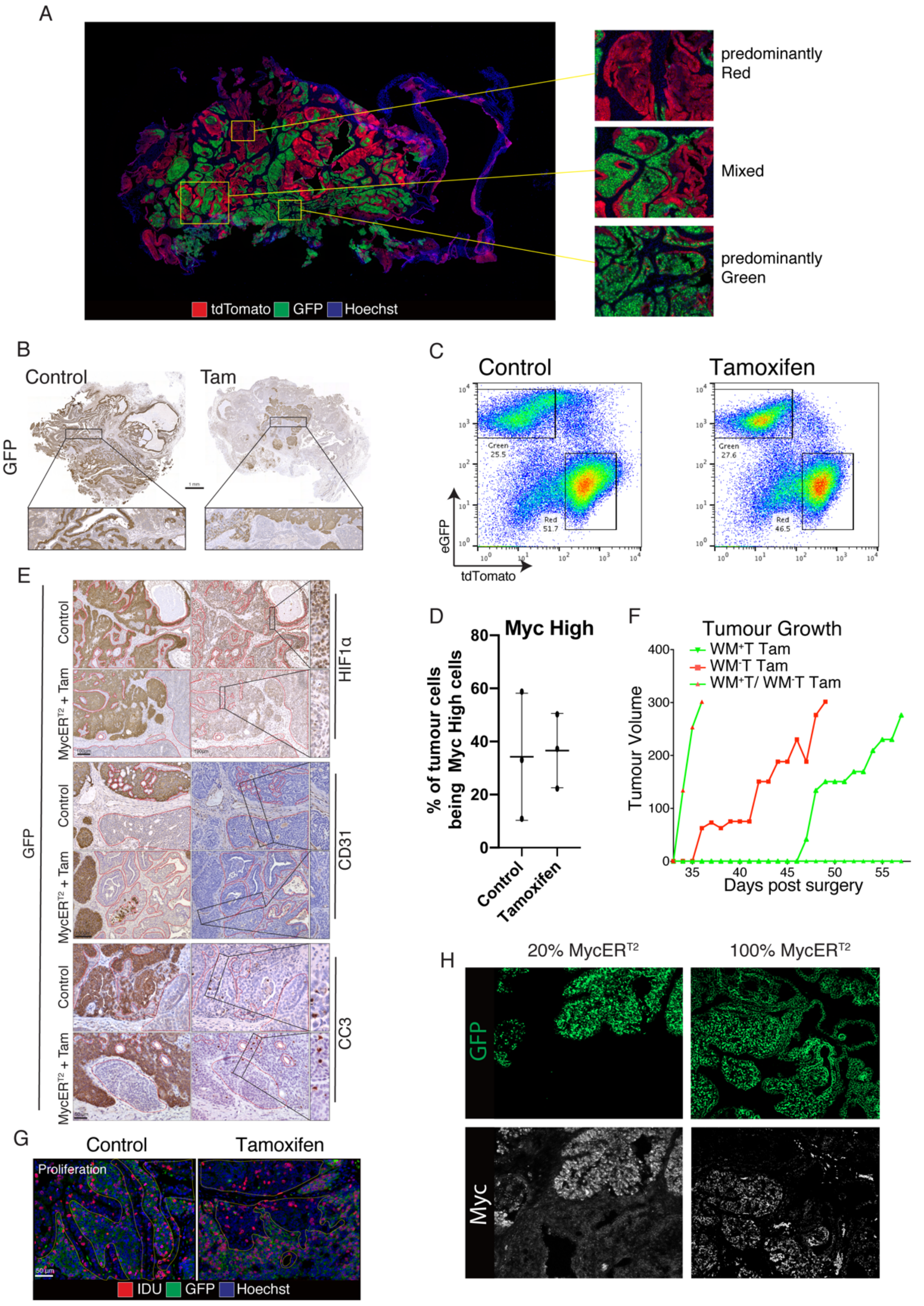
A
predominantly Red
Mixed
predominantly Green
tdTomato GFP Hoechst
B
Control
Tam
GFP
C
Control
Tamoxifen
Green 25.5
Red 51.7
Green 27.6
Red 46.5
eGFP
tdTomato
D
Myc High
% of tumour cells being Myc High cells
Control
Tamoxifen
E
GFP
Control
MycER^{T2} + Tam
HIF1α
CD31
CC3
F
Tumour Growth
WM^{+}T Tam
WM^{-}T Tam
WM^{+}T/ WM^{-}T Tam
Tumour Volume
Days post surgery
G
Control
Tamoxifen
Proliferation
IDU GFP Hoechst
H
20% MycER^{T2}
100% MycER^{T2}
GFP
Myc

Figure S2. Histology and clonal dynamic of mixed clonal tumours. Related to Figure 2.

a) Representative picture of the gross morphology of mixed (this case 30%/70%) *WM^+T/ WM^-T* tumours and representative fluorescent image of mixed tumour tissue harbouring Myc^{High} (green) and Myc^{Low} (red) clones, surrounded by host derived stromal cells (no colour). Nuclei are stained with Hoechst (blue). Insets show examples of predominatly red areas, predominantly green areas and areas of clonal mixing.
b) Representative whole slide scans reveal morphological differences induced by Tamoxifen treatment. Mixed *WM^+T/ WM^-T* tumours were stained for GFP to reveal *WM^+T* clones.
c) Flow cytometric analysis of mixed clonal tumours (30% *WM^+T*/70%*WM^-T*) treated with vehicle or Tamoxifen for three days (n=3).
d) Quantification of clonal distribution of mixed clonal tumours (30% *WM^+T*/70%*WM^-T*) treated with vehicle or Tamoxifen for three days (n=3).
e) Representative image of consecutive sections of mixed *WM^+T/ WM^-T* tumours and vehicle controls stained for GFP (*WM^+T*) and HIF1α as a measure of Hypoxia when nuclear, CD31 to identify vasculature and CC3 as a measure of cell death.
f) Caliperimetic measurement of tumour growth of individual *WM^+T, WM^-T*, mixed *WM^+T/ WM^-T* tumours and controls. Mice were put on Tamoxifen containing diet or control diet ten days post- surgery (long term treatment), and tumours measured daily.
g) Representative images of immunofluorescent staining for 5-Iodo-2′-deoxyuridine (IDU, red) injected two hours prior to sacrifice, EGFP as a marker for Cre-recombination (green) and DNA (Hoechst - blue), of mixed *WM^+T/ WM^-T* tumours and controls. IDU incorporation indicates cells in S-phase.
h) Representative images of immunofluorescence stainings for Myc and GFP of frozen tissue sections from long term treated *WM^+T* and mixed *WM^+T/ WM^-T* tumours showing loss of Myc in the WM^+T tumours, but no such phenomenon in the mixed *WM^+T/ WM^-T* tumours.

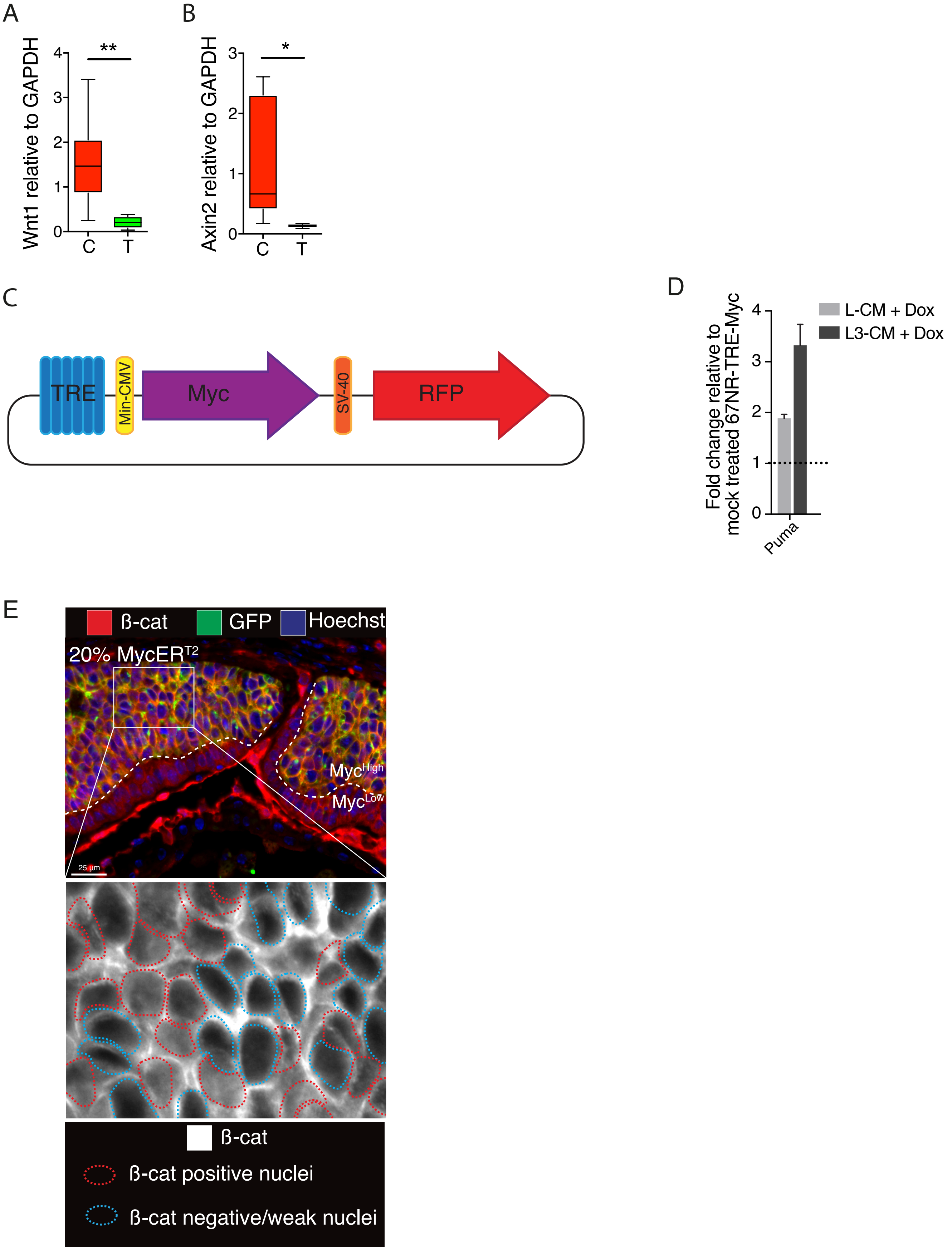
A
Wnt1 relative to GAPDH
**
0
1
2
3
4
C
T
B
Axin2 relative to GAPDH
*
0
1
2
3
C
T
C
TRE
Min-CMV
Myc
SV-40
RFP
D
Fold change relative to mock treated 67NR-TRE-Myc
0
1
2
3
4
L-CM + Dox
L3-CM + Dox
Puma
E
ß-cat
GFP
Hoechst
20% MycER^{T2}
Myc^{High}
Myc^{Low}
25 µm
ß-cat
ß-cat positive nuclei
ß-cat negative/weak nuclei

Figure S3. qRT-PCRs, vector map of Doxycycline inducible construct introduced into 67NR cell lines and Immunofluorescence for nuclear ß-catenin in in mixed $WM^{+}T$/ $WM^{-}T$ tumours in area removed from clonal interface. Related to Figure 3.

a) Quantitative real time PCR of whole tissue mRNA extracts from $WM^{+}T$ and $WM^{-}T$ control tumours for *Wnt1* relative to the expression of *Gapdh* as a housekeeping gene (n = 5 $WM^{+}T$ tumours and 11 controls, controls are: $WM^{-}T$ + Tamoxifen and + vehicle, $WM^{+}T$ plus vehicle).
b) Quantitative real time PCR of whole tissue mRNA extracts from $WM^{+}T$ and $WM^{-}T$ control tumours for *Axin2* relative to the expression of *Gapdh* as a housekeeping gene (n = 5 $WM^{+}T$ tumours and 11 controls, controls are: $WM^{-}T$ + Tamoxifen and + vehicle, $WM^{+}T$ plus vehicle).
c) Vector map of Doxycycline inducible construct introduced into 67NR cell lines. Note that Myc ad RFP are in tandem, each driven by their own promoter.
d) Quantitative realtime PCR for Puma in cellular extracts from 67NR-Myc-RFP cell lines treated for 24h with Doxycycline and media conditioned by control L-cells (L-CM) or Doxycycline and media conditioned by L-cells expressing recombinant Wnt3a (L3-CM), both normalised to respective untreated controls of the same cells treated with L-CM alone (n = 3, error bars represent standard deviation).
e) Immunofluorescence for nuclear ß-catenin in in mixed $WM^{+}T$/ $WM^{-}T$ tumours in area removed from clonal interface. Insets show grayscale picture of the ß-catenin staining alone. Nuclei were traced and represented as outlines. The colour designates, whether a nucleus was scored as positive or negative for nuclear ß-catenin.

A

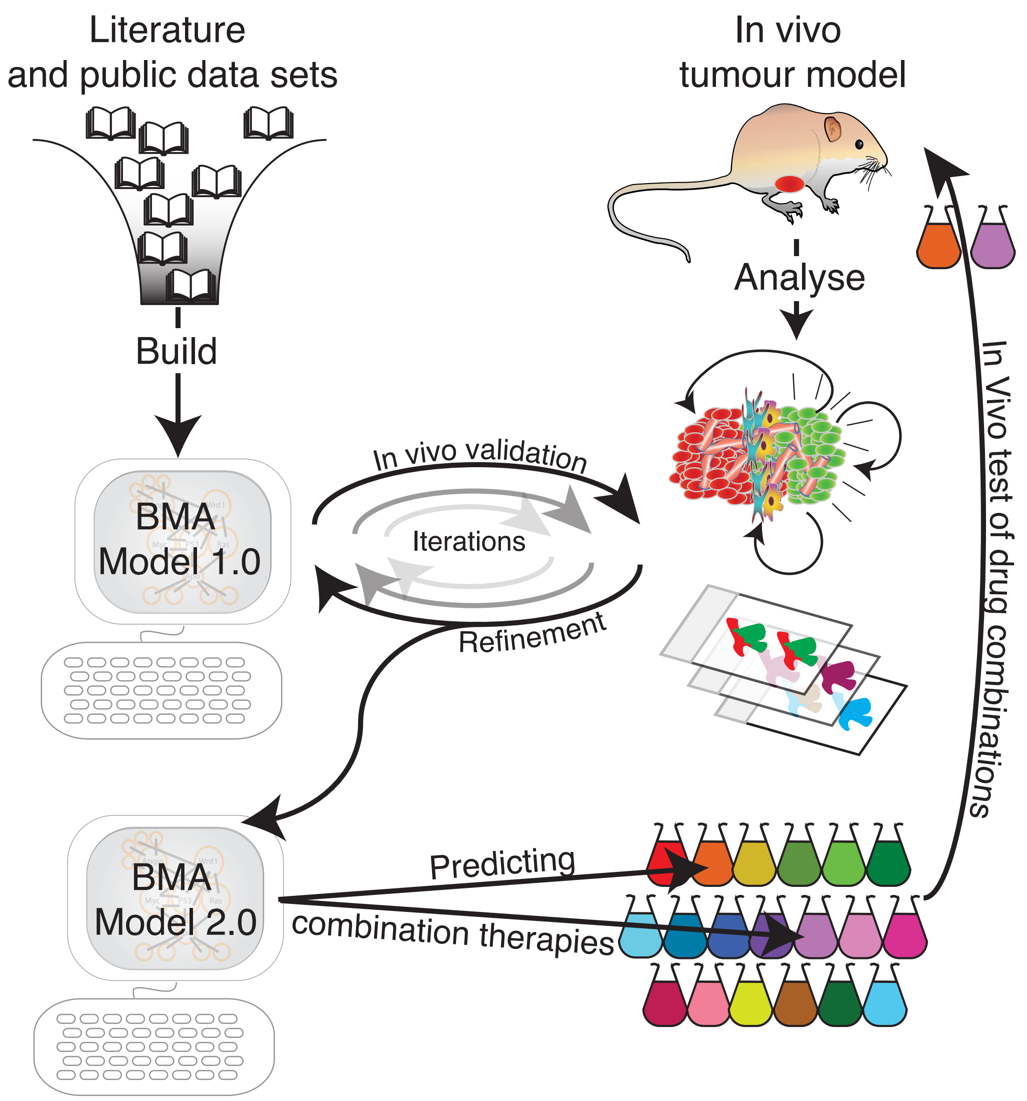
Literature
and public data sets
Build
BMA
Model 1.0
In vivo validation
Iterations
Refinement
In vivo
tumour model
Analyse
In Vivo test of drug combinations
BMA
Model 2.0
Predicting
combination therapies

B

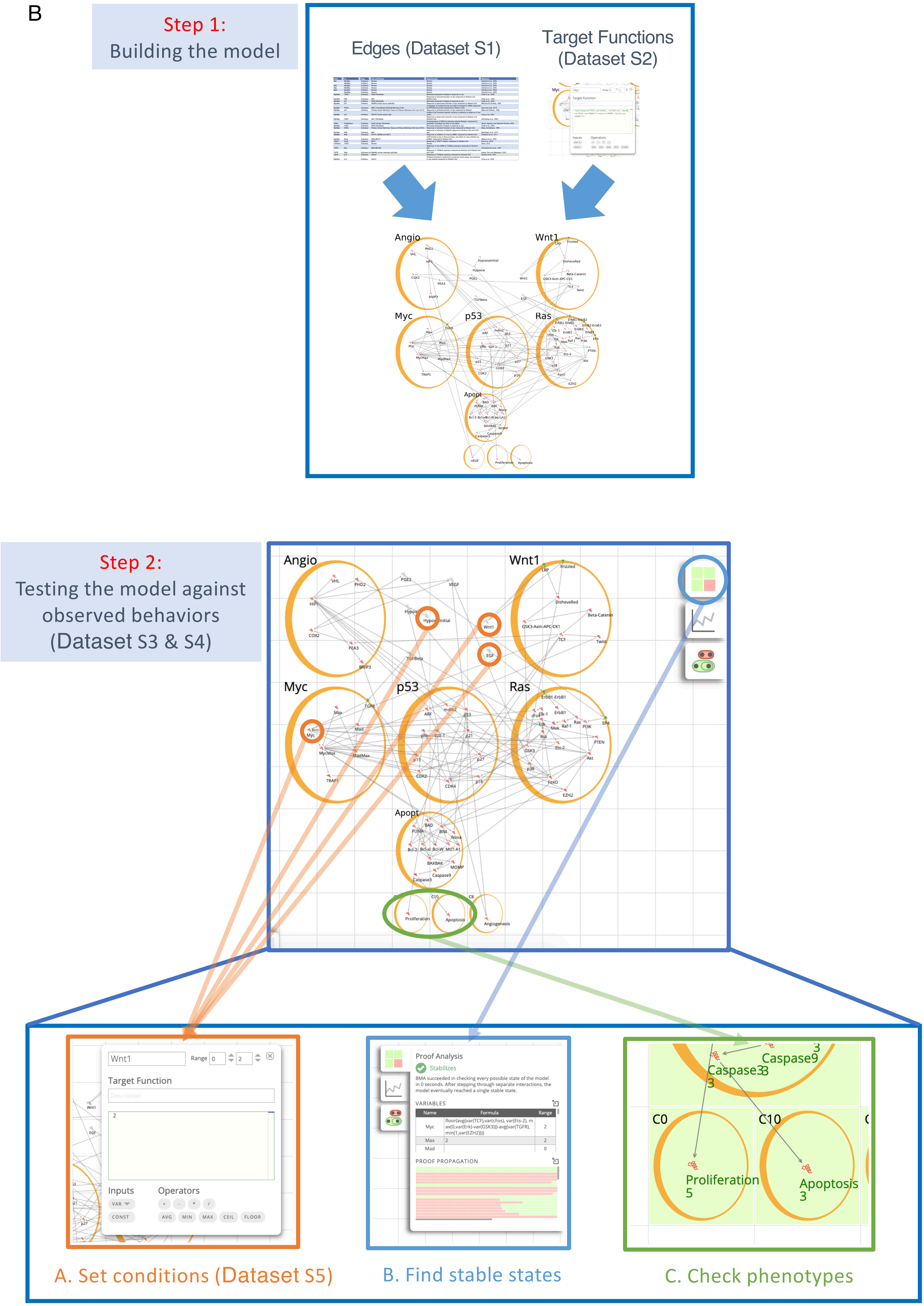
Step 1:
Building the model
Edges (Dataset S1)
Target Functions (Dataset S2)
Angio
Wnt1
Myc
p53
Ras
Apopt
Step 2:
Testing the model against observed behaviors (Dataset S3 & S4)
Angio
Wnt1
Myc
p53
Ras
Apopt
Wnt1
Range 0 2
Target Function
2
Inputs
Operators
VAR
CONST
AVG MIN MAX CEIL FLOOR
Proof Analysis
Stabilizes
BMA succeeded in checking every possible state of the model in 0 seconds. After stepping through several interactions, the model eventually reached a single stable state.
VARIABLES
Name Formula Range
Myc floor(avg(var(TCF),var(cFos), var(Ets-2), max(0,var(Erk)-var(GSK3)))-avg(var(TGFR), min(1,var(EZH2)))) 2
Max 2 2
Mad 0
PROOF PROPAGATION
Caspase9
3
Caspase3
3
C0
C10
Proliferation
5
Apoptosis
3
A. Set conditions (Dataset S5)
B. Find stable states
C. Check phenotypes

Step 3:
Model refinement leading to new predictions

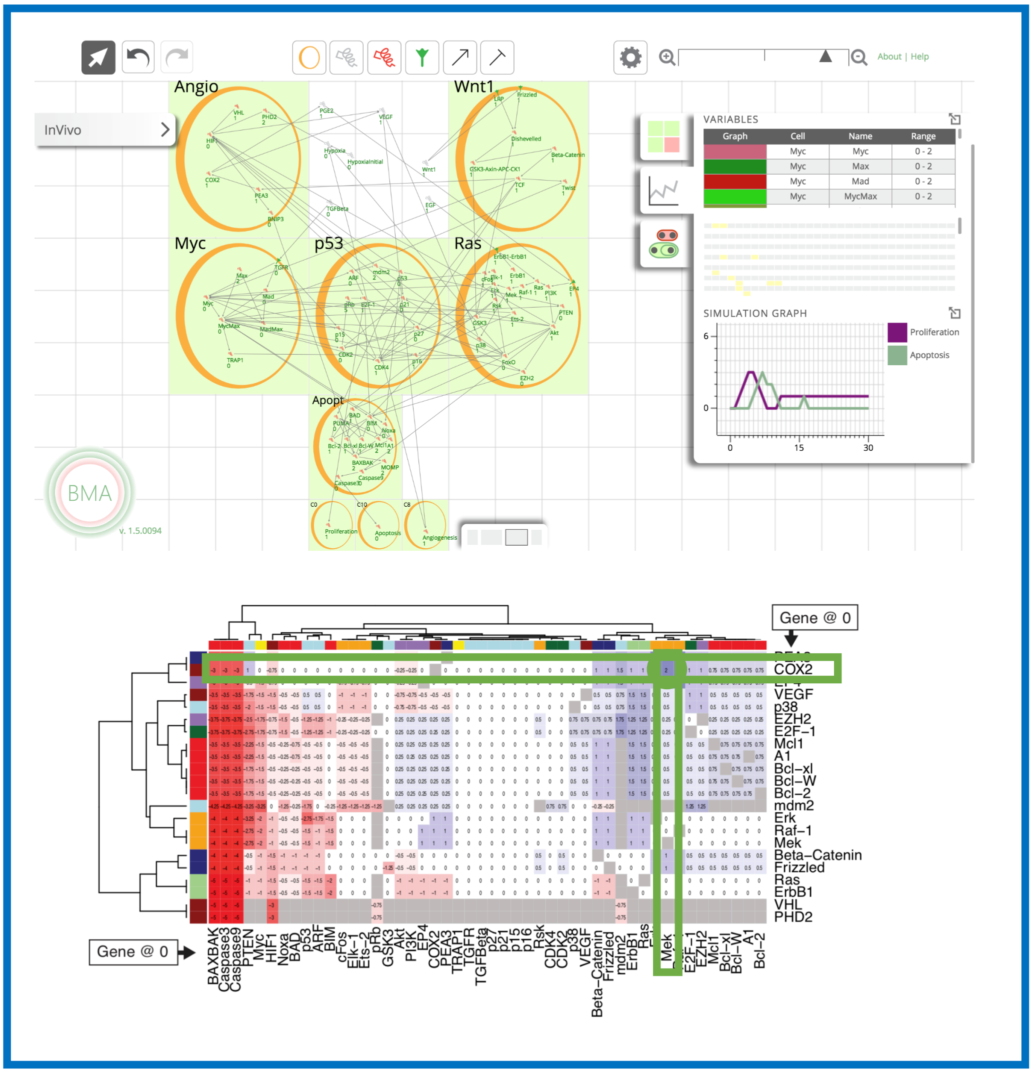

Step 4:
Experimental validation of predictions

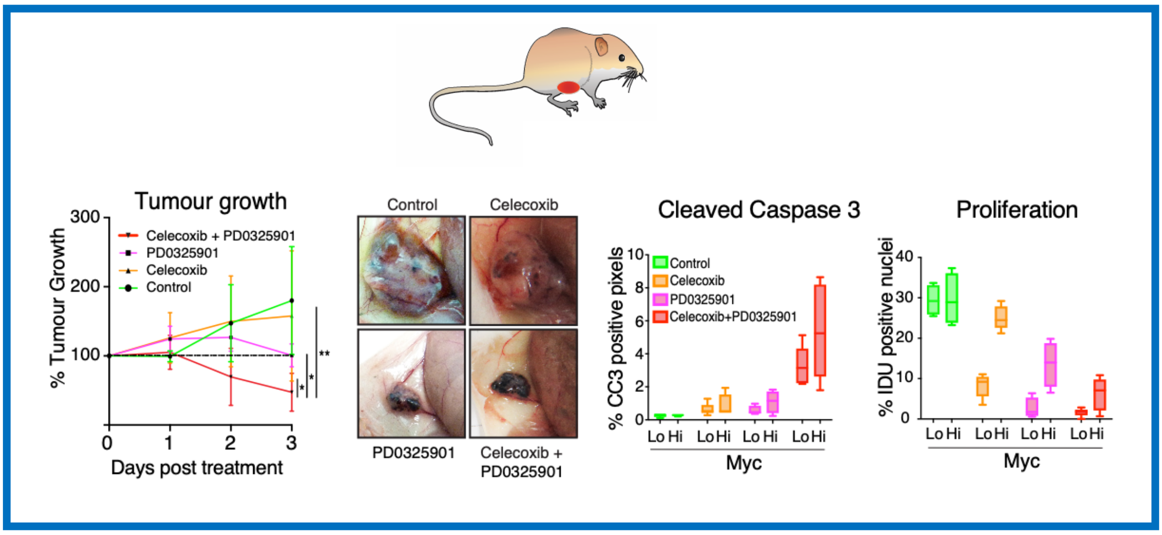

Figure S4.

a) Overview of the workflow used to iteratively build and validate the model against experiments in the literature and against our mouse model; generate predictions about the best mono- and combination therapies, and test them *in vivo*.
b) How the network model is established, tested and used to make predictions in more detail.

Step 1: We build the model from existing experimental evidence available in the literature. The evidence to support modelled interactions between nodes, referred to as edges, is detailed in Dataset S1. These interactions are characterised by the Target Functions for each node, which are detailed in Dataset S2. These are combined in a model built in the BioModelAnalyzer tool.

Step 2: We tested the model against known behaviours for breast cancer cells. These were either experiments in the literature performed on cell lines (Dataset S3), or new experiments on our mouse model (Dataset S4). To model these we set the target functions of nodes representing mutated or drugged genes, or external factors, to a constant value representing the perturbation (orange circles). We next tested to find the stable state of the network in the BMA GUI (blue circle, B) and also with the command-line tool. We compared the stable values of the nodes to those observed in experiment (green circle, C).

Step 3: We refined the model until it agreed with observations. We then set the network to mimic the clones within the mouse model tumours (Dataset S5). We added perturbations to the network representing inhibition of a node or a pair of nodes, to find those which would be the most effective drug targets to lower proliferation and increase apoptosis.

Step 4: We then chose the most effective combination to be tested in the mouse model.

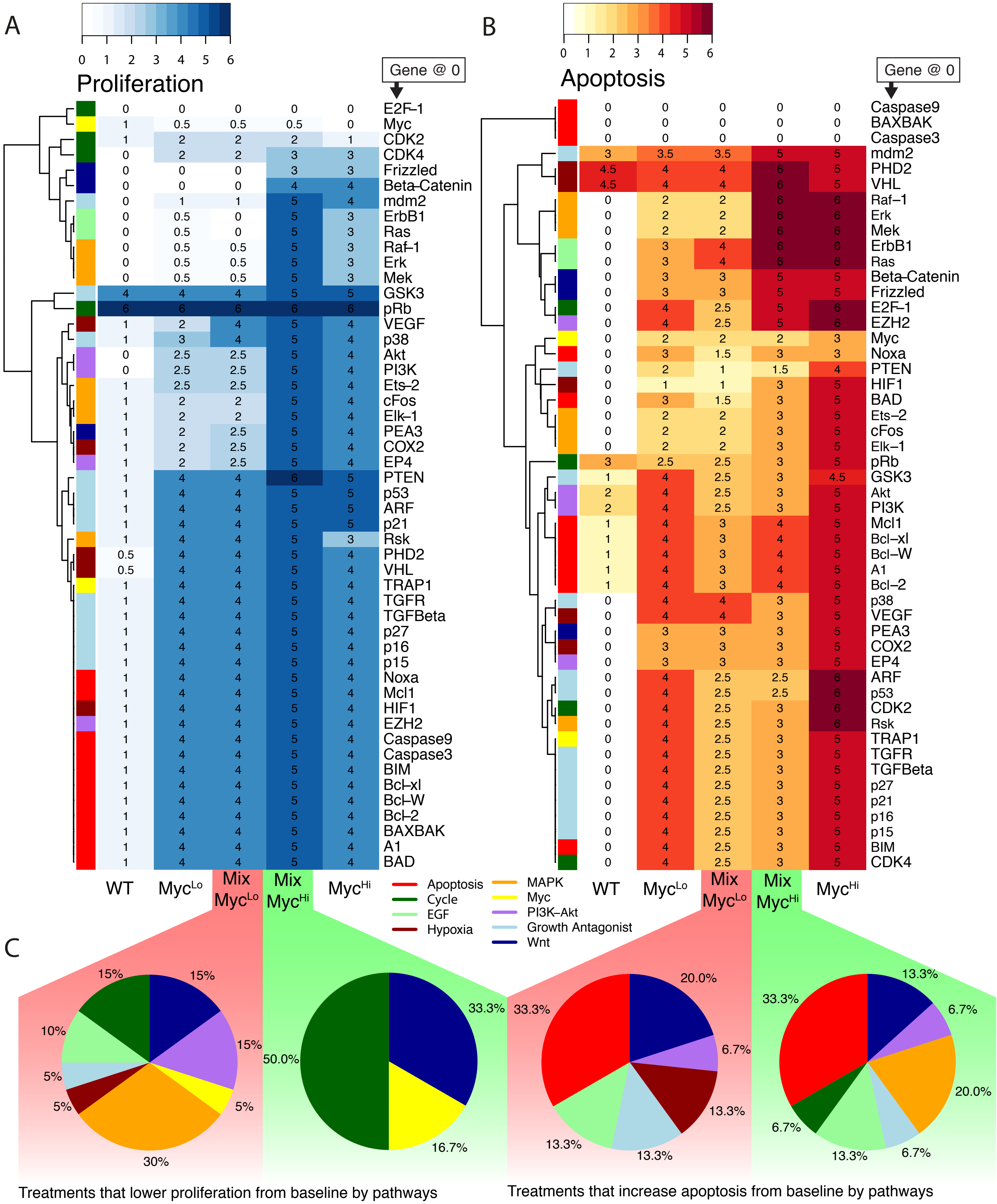
A
0 1 2 3 4 5 6
Proliferation
Gene @ 0
E2F–1
Myc
CDK2
CDK4
Frizzled
Beta-Catenin
mdm2
ErbB1
Ras
Raf–1
Erk
Mek
GSK3
pRb
VEGF
p38
Akt
PI3K
Ets–2
cFos
Elk–1
PEA3
COX2
EP4
PTEN
p53
ARF
p21
Rsk
PHD2
VHL
TRAP1
TGFR
TGFBeta
p27
p16
p15
Noxa
Mcl1
HIF1
EZH2
Caspase9
Caspase3
BIM
Bcl-xl
Bcl-W
Bcl-2
BAXBAK
A1
BAD
WT
Myc^Lo
Mix Myc^Lo
Mix Myc^Hi
Myc^Hi
B
0 1 2 3 4 5 6
Apoptosis
Gene @ 0
Caspase9
BAXBAK
Caspase3
mdm2
PHD2
VHL
Raf–1
Erk
Mek
ErbB1
Ras
Beta-Catenin
Frizzled
E2F–1
EZH2
Myc
Noxa
PTEN
HIF1
BAD
Ets–2
cFos
Elk–1
pRb
GSK3
Akt
PI3K
Mcl1
Bcl–xl
Bcl–W
A1
Bcl–2
p38
VEGF
PEA3
COX2
EP4
ARF
p53
CDK2
Rsk
TRAP1
TGFR
TGFBeta
p27
p21
p16
p15
BIM
CDK4
WT
Myc^Lo
Mix Myc^Lo
Mix Myc^Hi
Myc^Hi
Apoptosis
Cycle
EGF
Hypoxia
MAPK
Myc
PI3K–Akt
Growth Antagonist
Wnt
C
15%
15%
10%
15%
5%
5%
5%
30%
33.3%
50.0%
16.7%
Treatments that lower proliferation from baseline by pathways
33.3%
20.0%
6.7%
13.3%
13.3%
13.3%
13.3%
33.3%
6.7%
20.0%
6.7%
13.3%
6.7%
Treatments that increase apoptosis from baseline by pathways

Figure S5. Predicted effects of monotherapy on different clones and healthy cells and breakdown of which pathways are affected by successful treatments.
Changes to the signalling network to mimic different clones and healthy cells are shown in Dataset S5. Note that the cells of the heatmaps are coloured based on predicted activity, while rows are also coloured on the left based on pathways to which treated nodes belong. The pathway categories are laid out in Dataset S7.

a-b) Proliferation (a) or apoptosis (b) for all clones under a mutation of a single node. Rows are hierarchically clustered by similarity between effects.

c) (Left) Proportion of effective (proliferation lower than without treatment for that clone) single drug inhibitions in different pathways in the Myc^{low} and Myc^{high} clones in mixed tumours. The vulnerability of the Myc^{low} clone compared to the Myc^{high} clone is apparent with many more pathways leading to lower proliferation in the Myc^{low} case. The only way to affect proliferation in the Myc^{high} clone is via perturbation of the driving pathways, Myc and Wnt1, or via direct interference with the cell cycle. The Myc^{low} clone is more vulnerable to MAPK and PI3K-Akt perturbations, as Myc is downstream of these and these tumours must rely on activating the endogenous Myc to sustain proliferation. Cells in the Myc^{low} clone must also inhibit growth antagonists such as $p21^{cip1}$ and p27 through the PI3K-Akt pathway in order to benefit from the Wnt1 pathway, whereas Myc suppresses these in the Myc^{high} clone. (Right) Proportion of effective (apoptosis higher than without treatment in that clone) single inhibitions in different pathways in the Myc^{low} and the Myc^{high} clones in mixed tumours. The effective treatments for each clone are more similar when considering apoptosis. However, the best pathways for specific treatments are also clearly apparent, with MAPK only being present in the Myc^{high} case, or the hypoxia/angiogenesis response pathway in the Myc^{low} case.

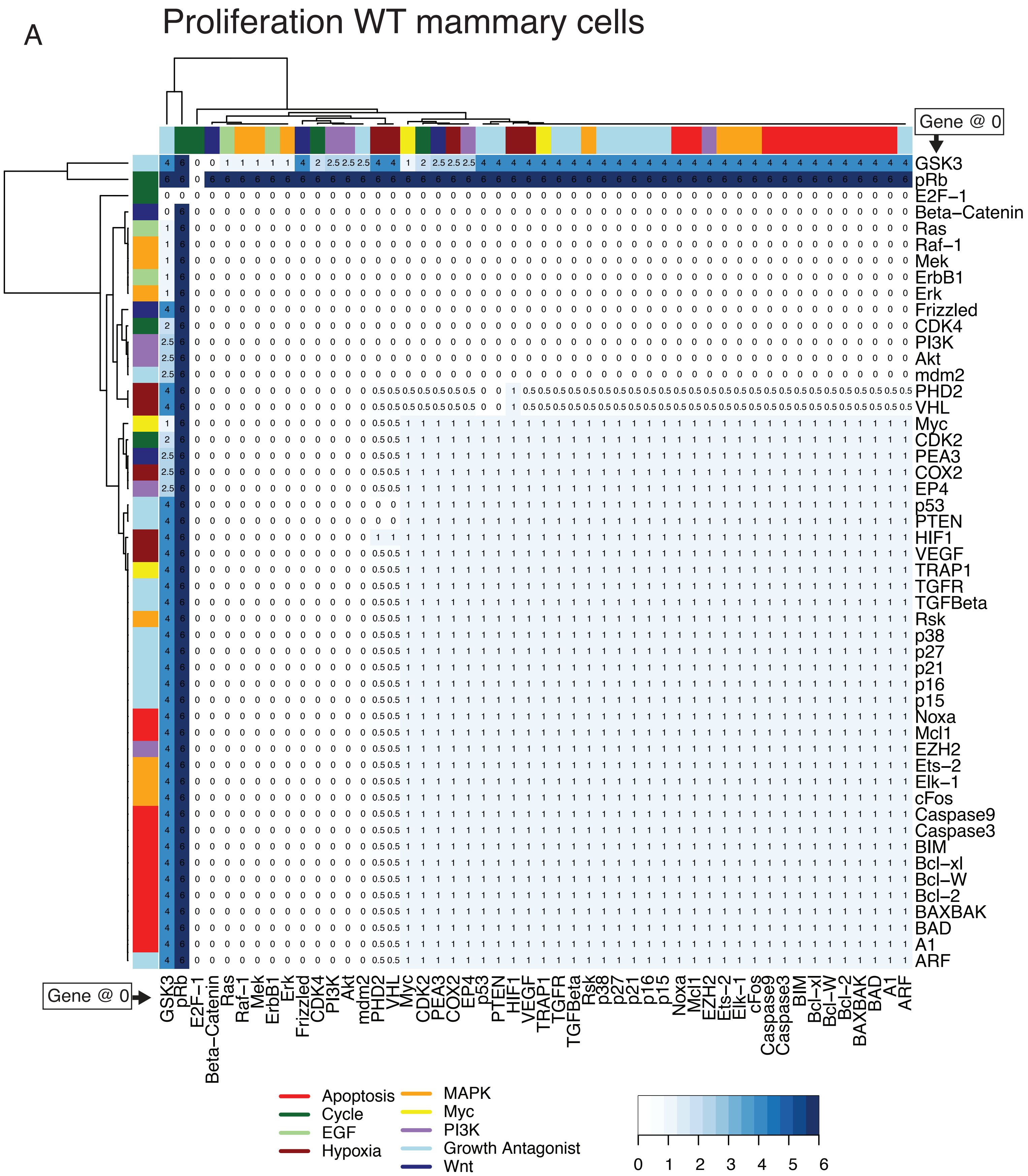
A
Proliferation WT mammary cells
Gene @ 0
GSK3
pRb
E2F−1
Beta−Catenin
Ras
Raf−1
Mek
ErbB1
Erk
Frizzled
CDK4
PI3K
Akt
mdm2
PHD2
VHL
Myc
CDK2
PEA3
COX2
EP4
p53
PTEN
HIF1
VEGF
TRAP1
TGFR
TGFBeta
Rsk
p38
p27
p21
p16
p15
Noxa
Mcl1
EZH2
Ets−2
Elk−1
cFos
Caspase9
Caspase3
BIM
Bcl−xl
Bcl−W
Bcl−2
BAXBAK
BAD
A1
ARF
Apoptosis
Cycle
EGF
Hypoxia
MAPK
Myc
PI3K
Growth Antagonist
Wnt
0
1
2
3
4
5
6

B Proliferation WM-T Tumours

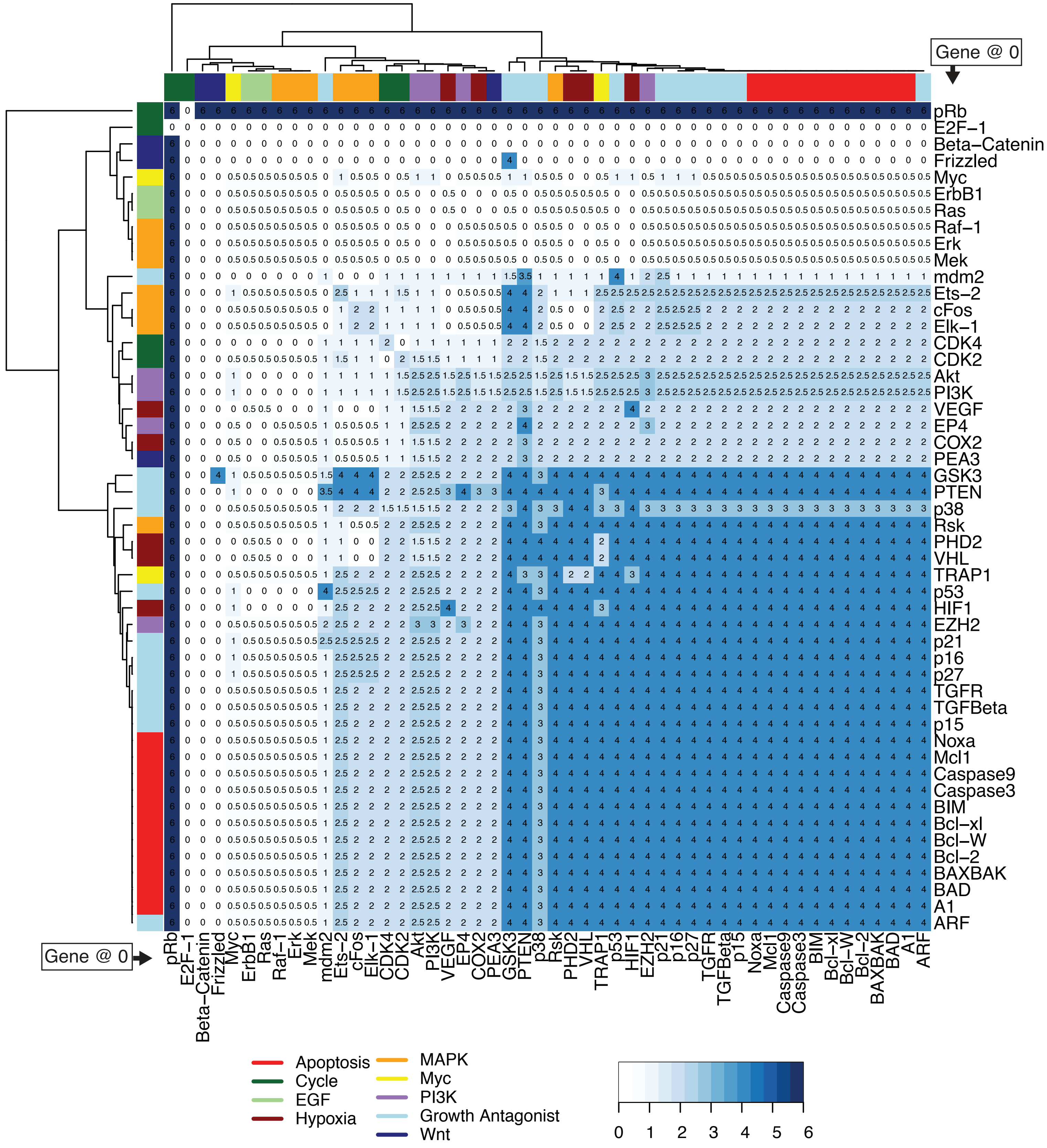

C

Proliferation $WM^{+}T$ Tumours

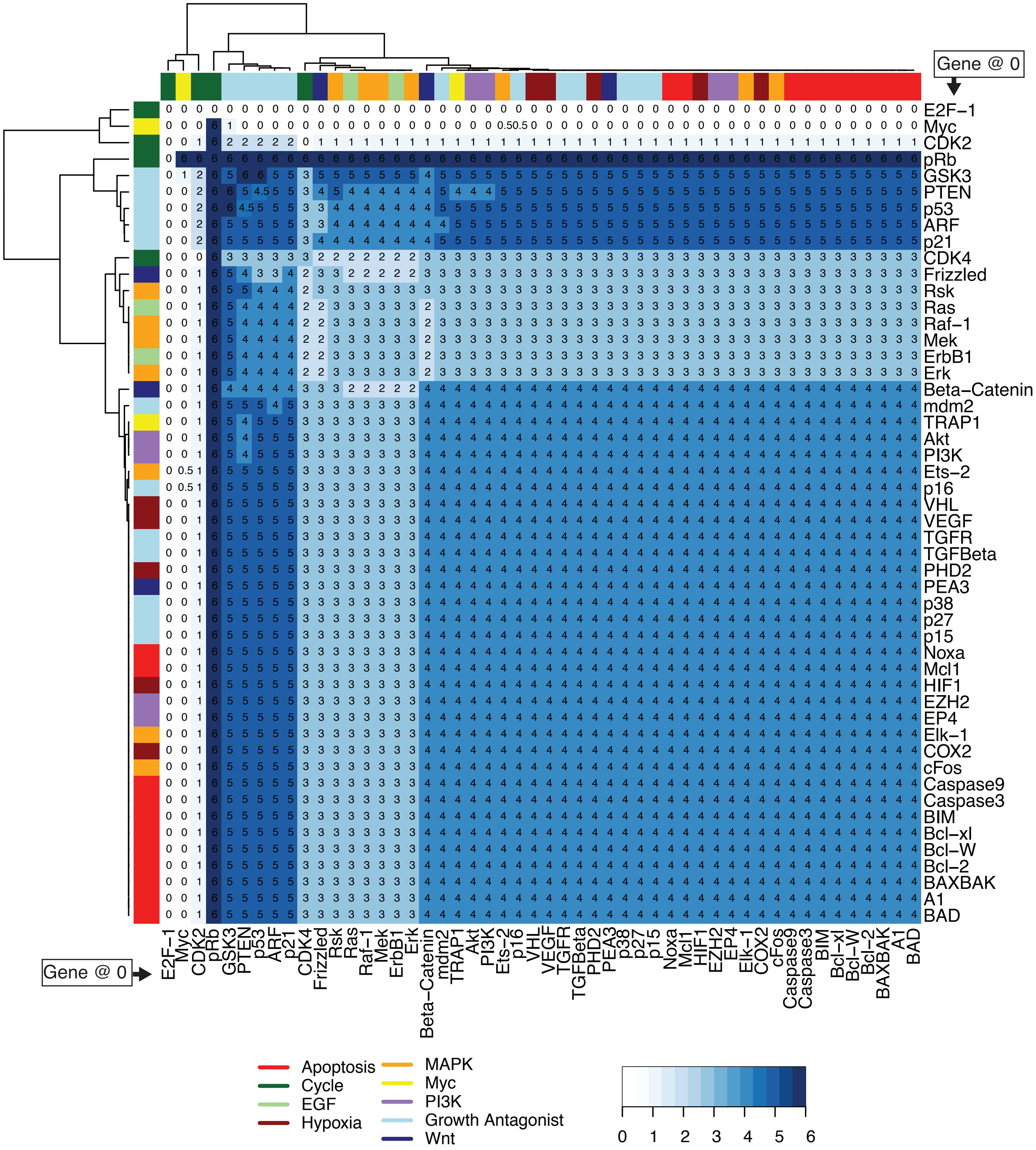

D Proliferation Myc^{Low} clone in mixed clonal tumours

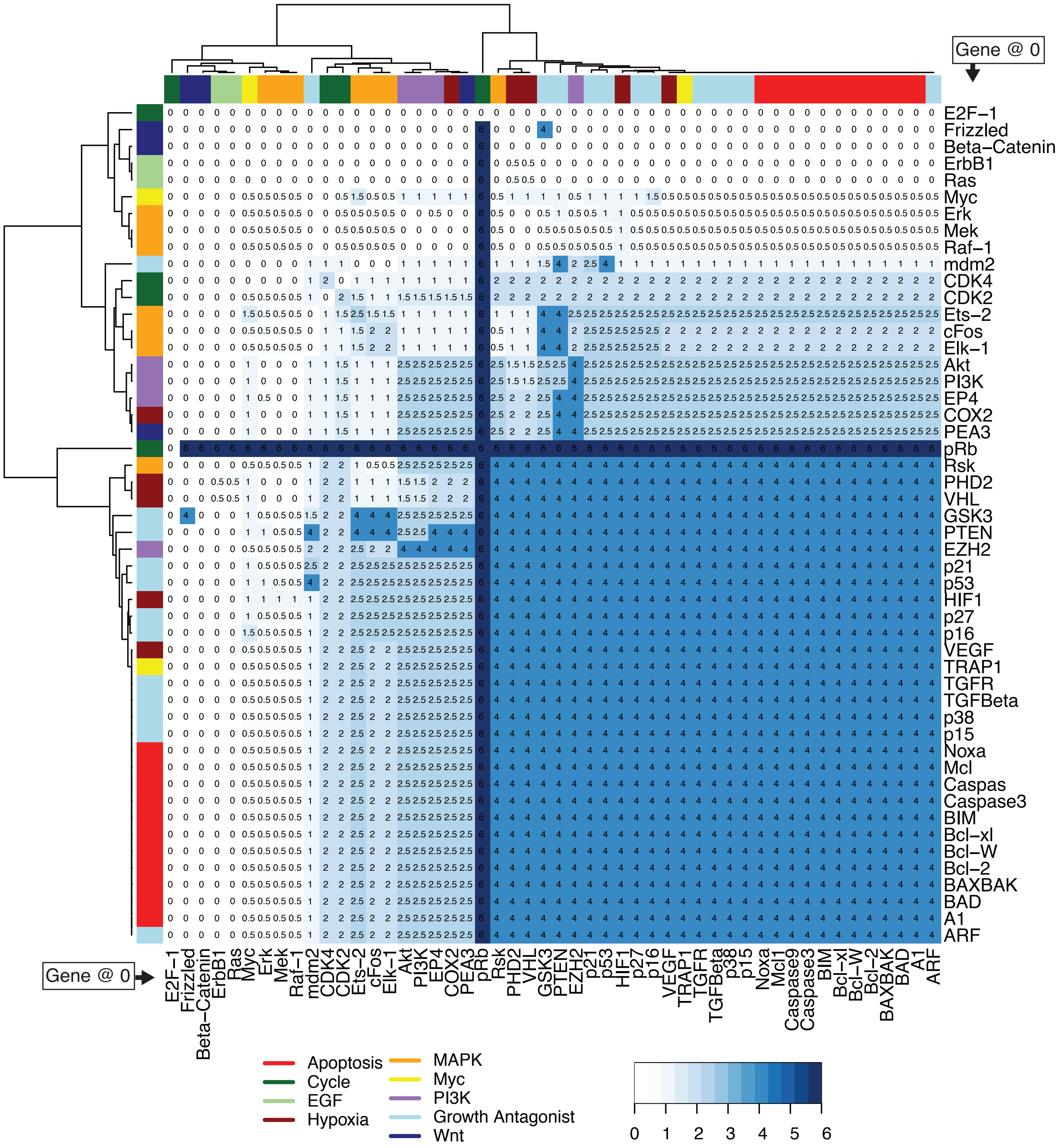

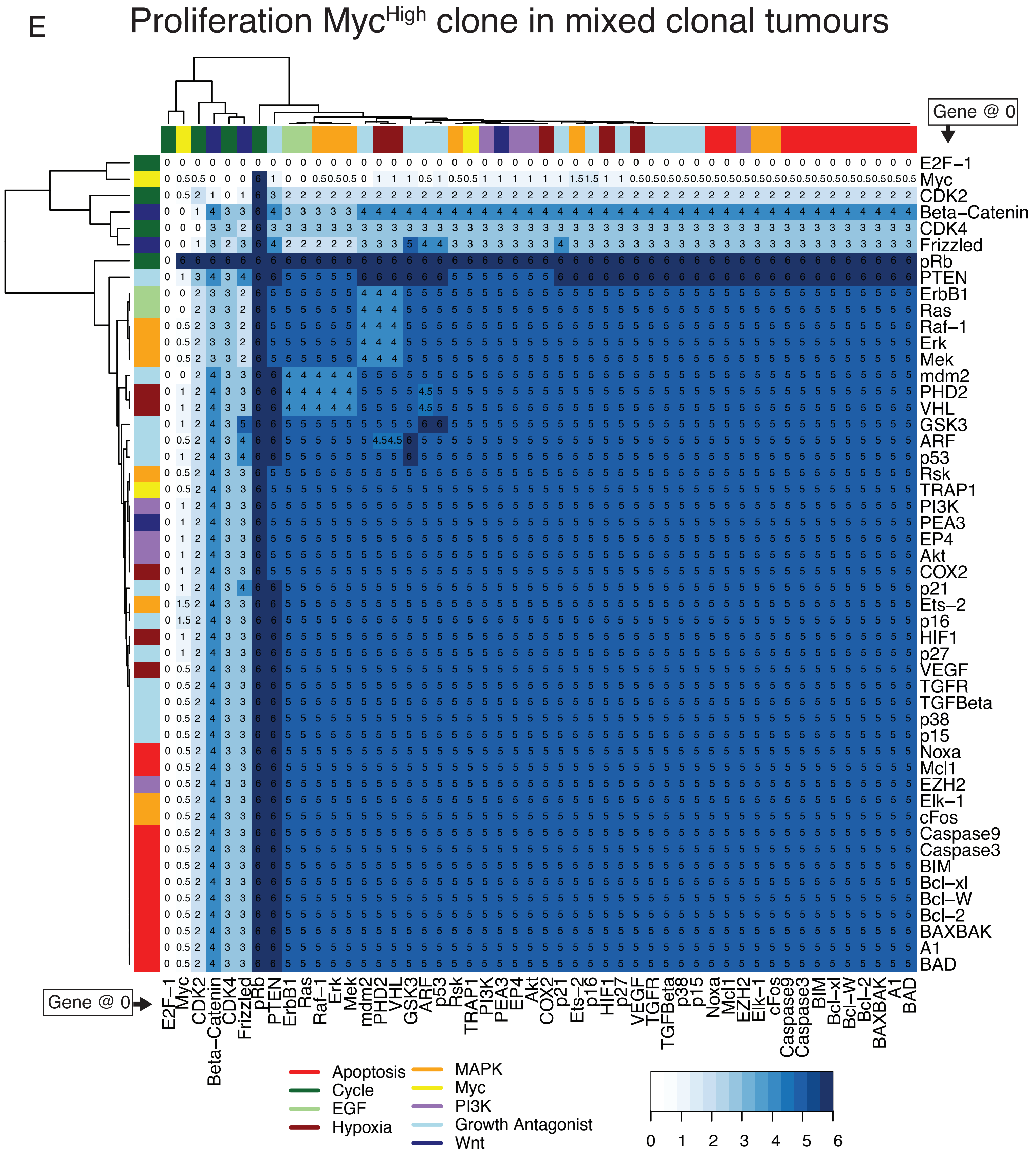

E
Proliferation Myc^High clone in mixed clonal tumours
Gene @ 0
Gene @ 0
E2F−1
Myc
CDK2
Beta−Catenin
CDK4
Frizzled
pRb
PTEN
ErbB1
Ras
Raf−1
Erk
Mek
mdm2
PHD2
VHL
GSK3
ARF
p53
Rsk
TRAP1
PI3K
PEA3
EP4
Akt
COX2
p21
Ets−2
p16
HIF1
p27
VEGF
TGFR
TGFBeta
p38
p15
Noxa
Mcl1
EZH2
Elk−1
cFos
Caspase9
Caspase3
BIM
Bcl−xl
Bcl−W
Bcl−2
BAXBAK
A1
BAD
Apoptosis
Cycle
EGF
Hypoxia
MAPK
Myc
PI3K
Growth Antagonist
Wnt
0
1
2
3
4
5
6

Figure S6. Predicted proliferation for different tumours and healthy cells under simulated combination therapy.

Predicted proliferation for: a) healthy cells, b) $WM^{-}T$ tumours, c) $WM^{+}T$ tumours and mixed tumours (d) Myc^{Low} clone and e) Myc^{High} clone when simulating the effect of two drugs in combination.

Note that the cells of the heatmaps are coloured based on predicted activity (the mean of the upper and lower bounds of proliferation across all reachable attractors), while each axis is also coloured on the left based on pathways to which treated nodes belong. Changes to the network target functions to mimic the different clones and healthy cells in panels a-e are shown in Table S5. The pathway categories are laid out in Table S7.

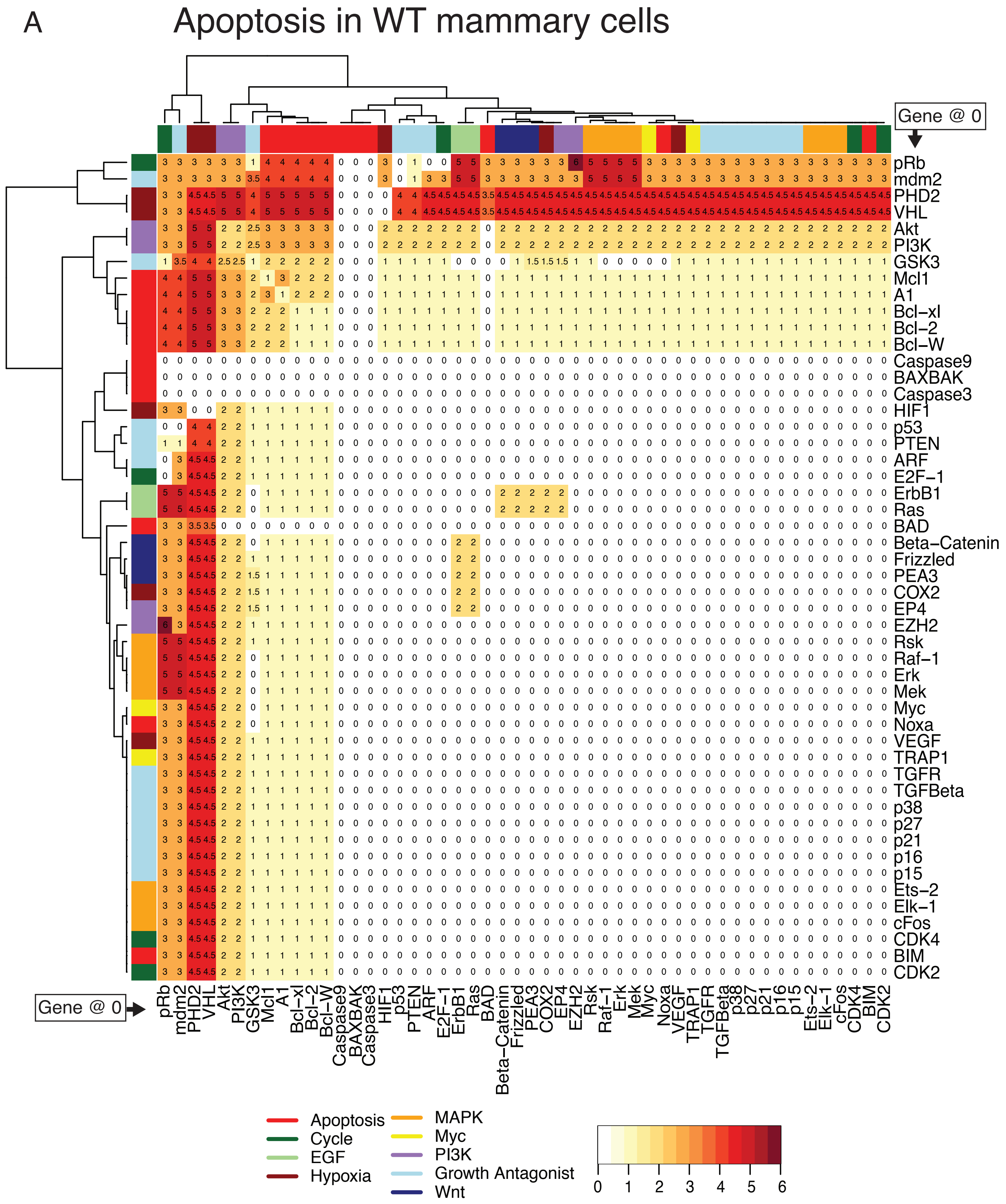
A
Apoptosis in WT mammary cells
Gene @ 0
pRb
mdm2
PHD2
VHL
Akt
PI3K
GSK3
Mcl1
A1
Bcl−xl
Bcl−2
Bcl−W
Caspase9
BAXBAK
Caspase3
HIF1
p53
PTEN
ARF
E2F−1
ErbB1
Ras
BAD
Beta−Catenin
Frizzled
PEA3
COX2
EP4
EZH2
Rsk
Raf−1
Erk
Mek
Myc
Noxa
VEGF
TRAP1
TGFR
TGFBeta
p38
p27
p21
p16
p15
Ets−2
Elk−1
cFos
CDK4
BIM
CDK2
Apoptosis
Cycle
EGF
Hypoxia
MAPK
Myc
PI3K
Growth Antagonist
Wnt
0
1
2
3
4
5
6

B

Apoptosis in WM-T Tumours

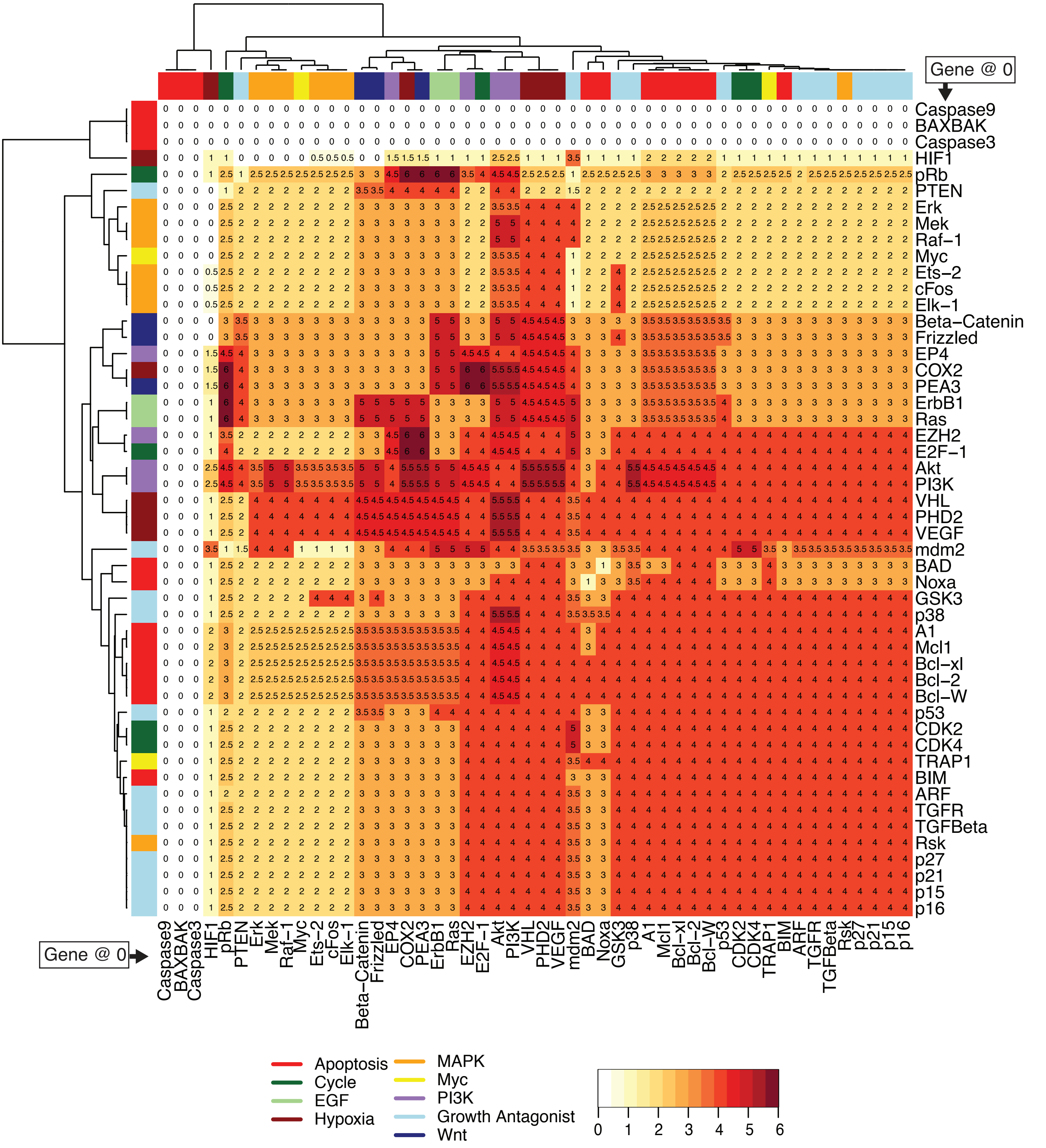

C

Apoptosis in $WM^{+}T$ Tumours

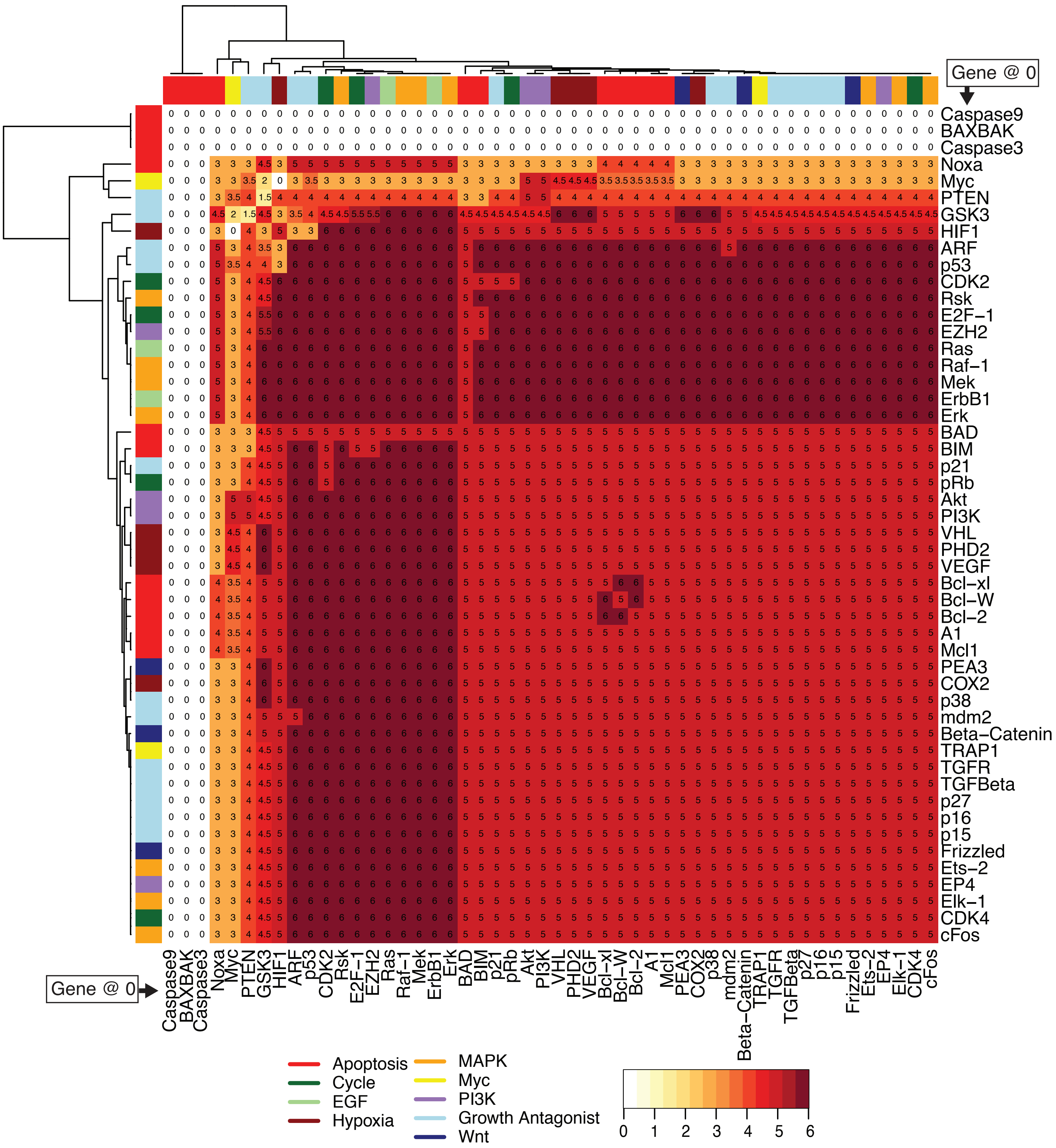

D Apoptosis Myc^{Low} clone in mixed clonal tumours

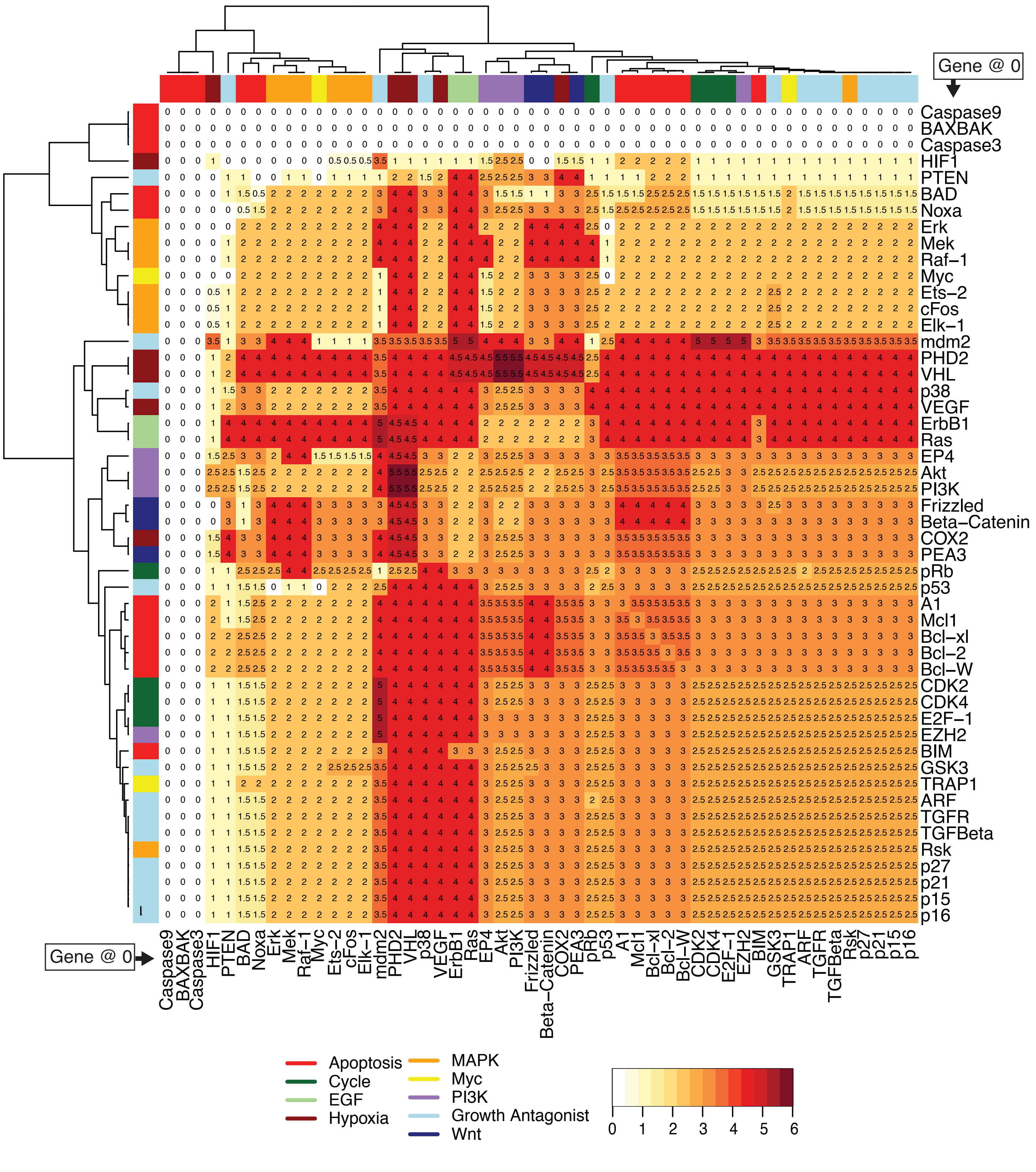

E Apoptosis Myc^High clone in mixed clonal tumours

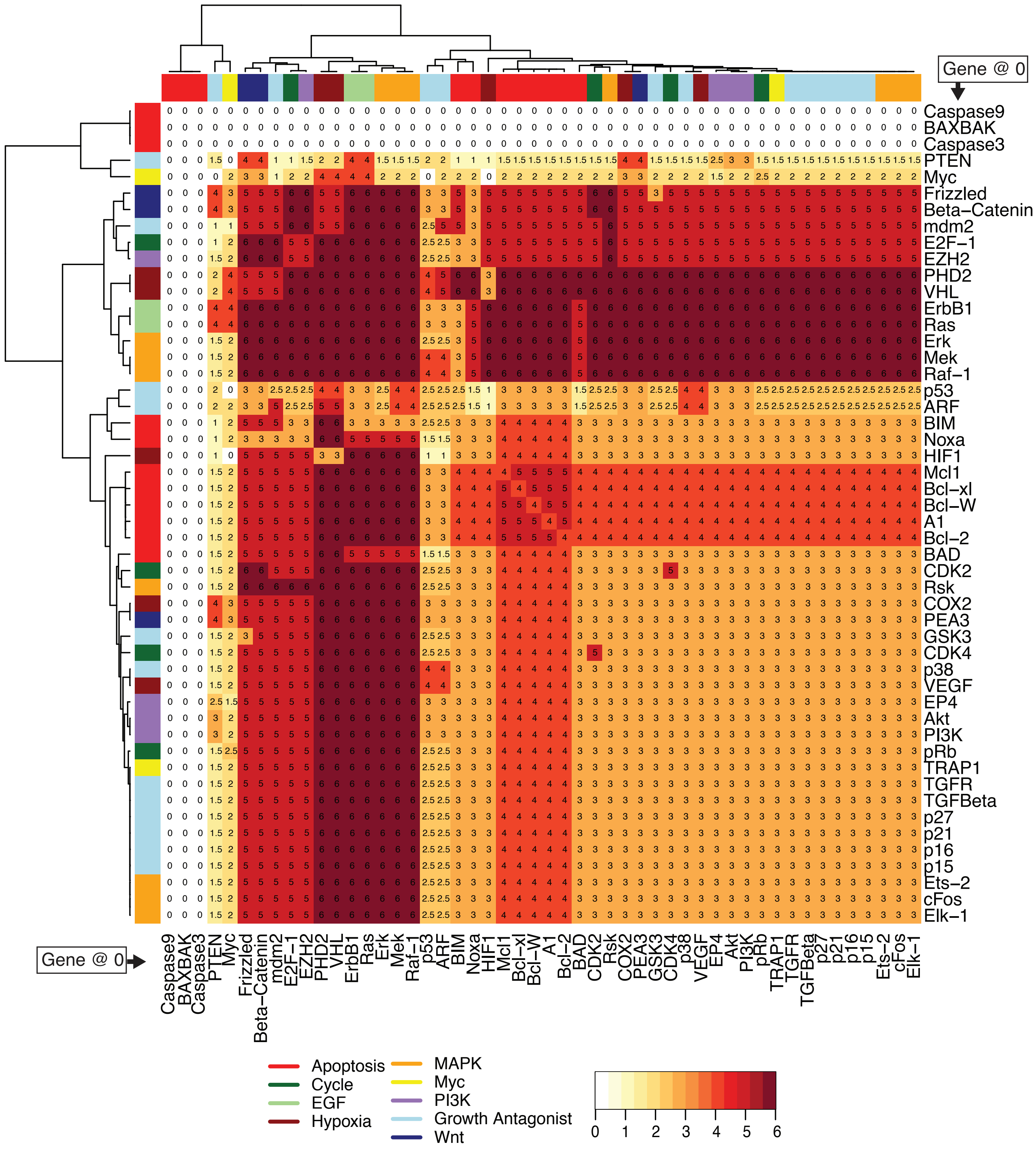

Figure S7. Predicted apoptosis for different tumours and healthy cells under simulated combination therapy.

Predicted apoptosis for a) healthy cells, b) $WM^{-}T$ tumours, c) $WM^{+}T$ tumours and mixed tumours (d) Myc^{Low} clone and e) Myc^{High} clone when simulating the effect of two drugs in combination. Note that the cells of the heatmaps are coloured based on predicted activity (the mean of the upper and lower bounds of activity across all reachable attractors), while each axis is also coloured on the left based on pathways to which treated nodes belong. Changes to the network target functions to mimic the different clones and healthy cells in panels a-e are shown in Dataset S5. The pathway categories are laid out in Dataset S7.

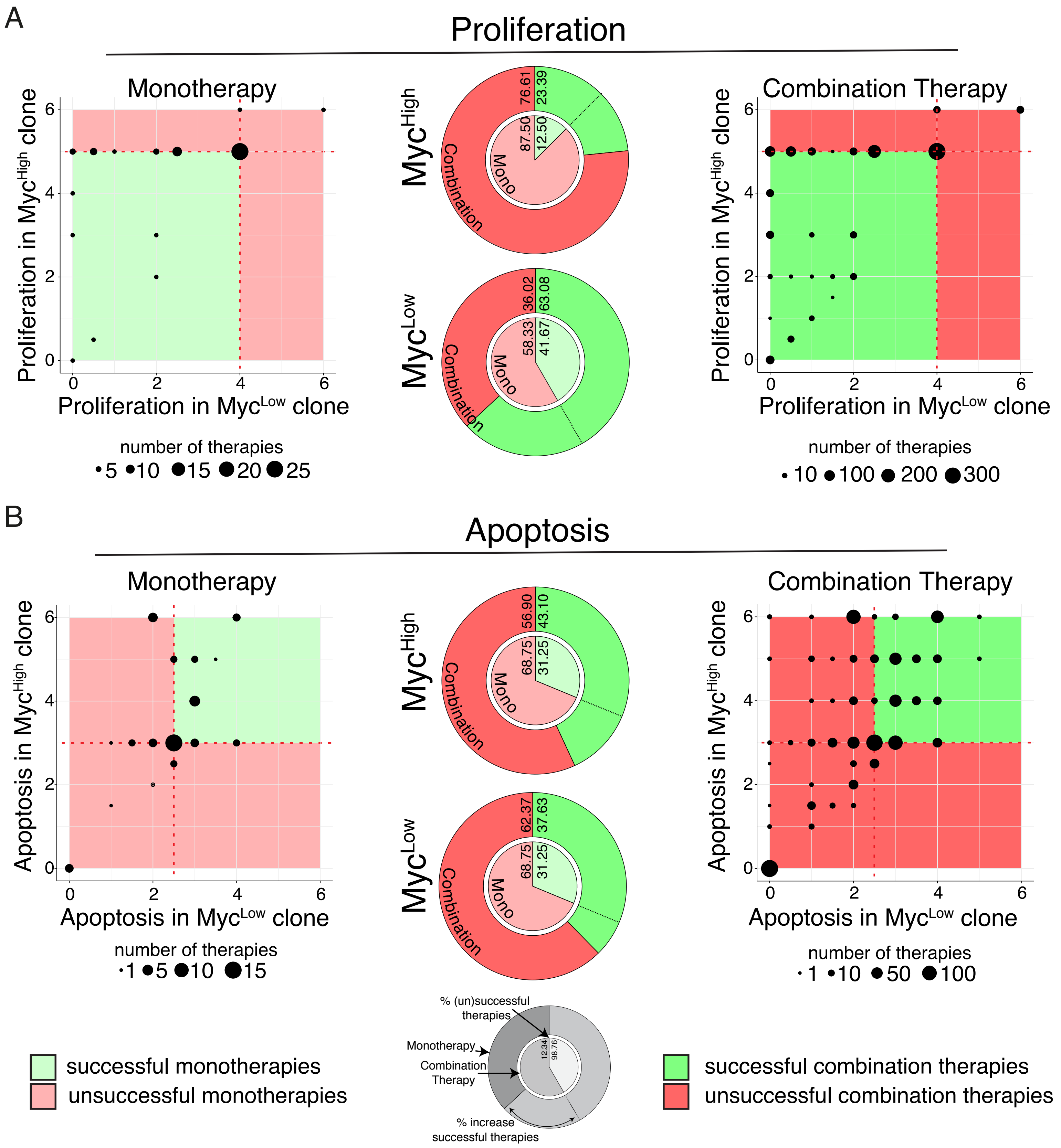
A
Proliferation
Monotherapy
Proliferation in MycHigh clone
Proliferation in MycLow clone
number of therapies
5 10 15 20 25
MycHigh
76.61
23.39
87.50
12.50
Mono
Combination
MycLow
36.02
63.08
58.33
41.67
Mono
Combination
Combination Therapy
Proliferation in MycHigh clone
Proliferation in MycLow clone
number of therapies
10 100 200 300
B
Apoptosis
Monotherapy
Apoptosis in MycHigh clone
Apoptosis in MycLow clone
number of therapies
1 5 10 15
MycHigh
56.90
43.10
68.75
31.25
Mono
Combination
MycLow
62.37
37.63
68.75
31.25
Mono
Combination
Combination Therapy
Apoptosis in MycHigh clone
Apoptosis in MycLow clone
number of therapies
1 10 50 100
% (un)successful therapies
Monotherapy
Combination Therapy
12.34
98.76
% increase successful therapies
successful monotherapies
unsuccessful monotherapies
successful combination therapies
unsuccessful combination therapies

Figure S8. Effects of monotherapy and combination therapy in Myc^{low} *vs* Myc^{high} clones in mixed tumours, and breakdown of proportion of effective treatments by single or combination therapy. See also Figures S5 and S6.

(Scatter plots: left and right) Scatter plot of the different levels of proliferation (a) or apoptosis (b) predicted by the network model under single (left) or combination (right) treatment. X-axis indicates the resulting level of proliferation or apoptosis in the Myc^{low} clone in mixed tumours and the y-axis the level in the Myc^{high} clone in mixed tumours. The size of the points indicates the number of different targets (or pairs of targets for combination therapy) whose inhibition produces this level of proliferation or apoptosis. The green shaded region of the plot indicates cases where proliferation was predicted to be lower, or apoptosis higher, than without treatment for both the Myc^{low} and Myc^{high} clones in mixed tumours and the red shaded region shows where proliferation was predicted to be higher, or apoptosis lower, than without treatment for either of the Myc^{low} or Myc^{high} clones in mixed tumours. This shows that there are many targets whose inhibition affects apoptosis in both clones, but proliferation only in the Myc^{low} clone.

(Pie charts: centre) Proportion of modelled therapies which lower proliferation (a) or raise apoptosis (b) for monotherapy (inner) and combination therapy (outer), in the Myc^{low} and Myc^{high} clones in mixed tumours. The overall pattern relating to which inhibitors are effective in combinations largely follows that established in monotherapy. Nonetheless, the proportion of successful treatments, where proliferation is lower than that without treatment for that clone, or apoptosis higher than without treatment, is greater for combination therapy. As the model is entirely deterministic, and the initial conditions differ only in respect of the inhibitors being simulated, this implies that there exist combinations that improve over the therapeutic effect of a single therapy as well as some that antagonize one another.

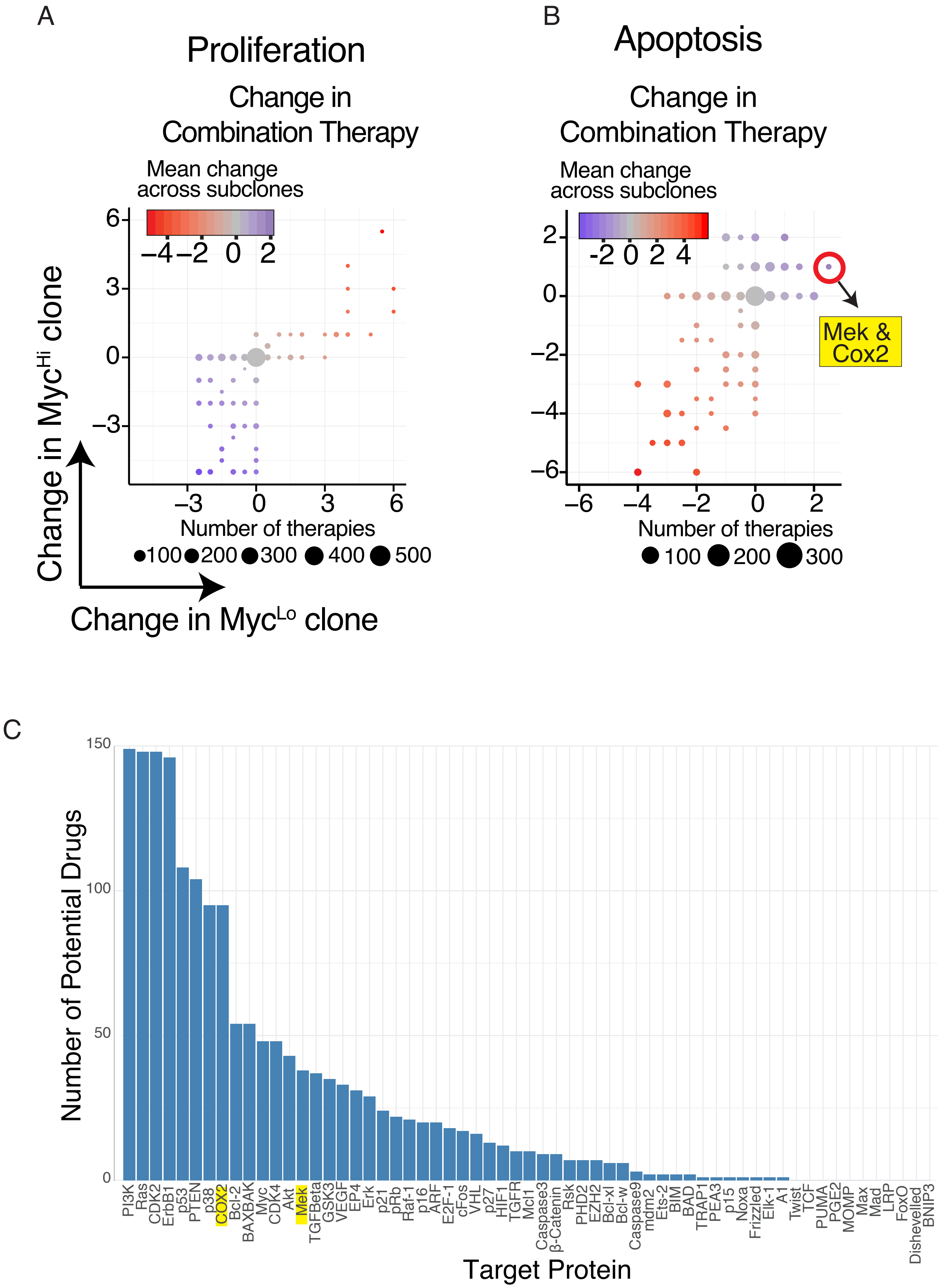
A
Proliferation
Change in
Combination Therapy
Mean change
across subclones
−4 −2 0 2
6
3
0
−3
−3 0 3 6
Change in Myc^{Hi} clone
Number of therapies
100 200 300 400 500
Change in Myc^{Lo} clone
B
Apoptosis
Change in
Combination Therapy
Mean change
across subclones
-2 0 2 4
2
0
−2
−4
−6
−6 −4 −2 0 2
Mek &
Cox2
Number of therapies
100 200 300
C
150
100
50
0
Number of Potential Drugs
PI3K
Ras
CDK2
ErbB1
p53
PTEN
p38
COX2
Bcl-2
BAXBAK
Myc
CDK4
Akt
Mek
TGFBeta
GSK3
VEGF
EP4
Erk
p21
pRb
Raf-1
p16
ARF
E2F-1
cFos
VHL
p27
HIF1
TGFR
Mcl1
Caspase3
β-Catenin
Rsk
PHD2
EZH2
Bcl-xl
Bcl-w
Caspase9
mdm2
Ets-2
BIM
BAD
TRAP1
PEA3
p15
Noxa
Frizzled
Elk-1
A1
Twist
TCF
PUMA
PGE2
MOMP
Max
Mad
LRP
FoxO
Dishevelled
BNIP3
Target Protein

Figure S9. Change in effect of therapy when adding a second inhibitor predicted by the computational model, and successful MEK and COX2 inhibition combination therapy *in vivo*. See also Figures S5, S6 and S7.

a-b) Scatter plot showing the number of combinations that produce each possible change in the value of proliferation in each clone, and the mean in both, when going from a single inhibitor to a combination.

c) Number of distinct drugs which interact with genes according to the Drug-Gene Interaction Database (Wagner et al., 2016) accessed using the package rDGIdb (Thurnherr *et al.*, 2016). This was to exclude those genes which had not known drug interactions whatsoever, while preserving those for which there were known interactions for further analysis, without consideration of the nature of the interaction at this stage.

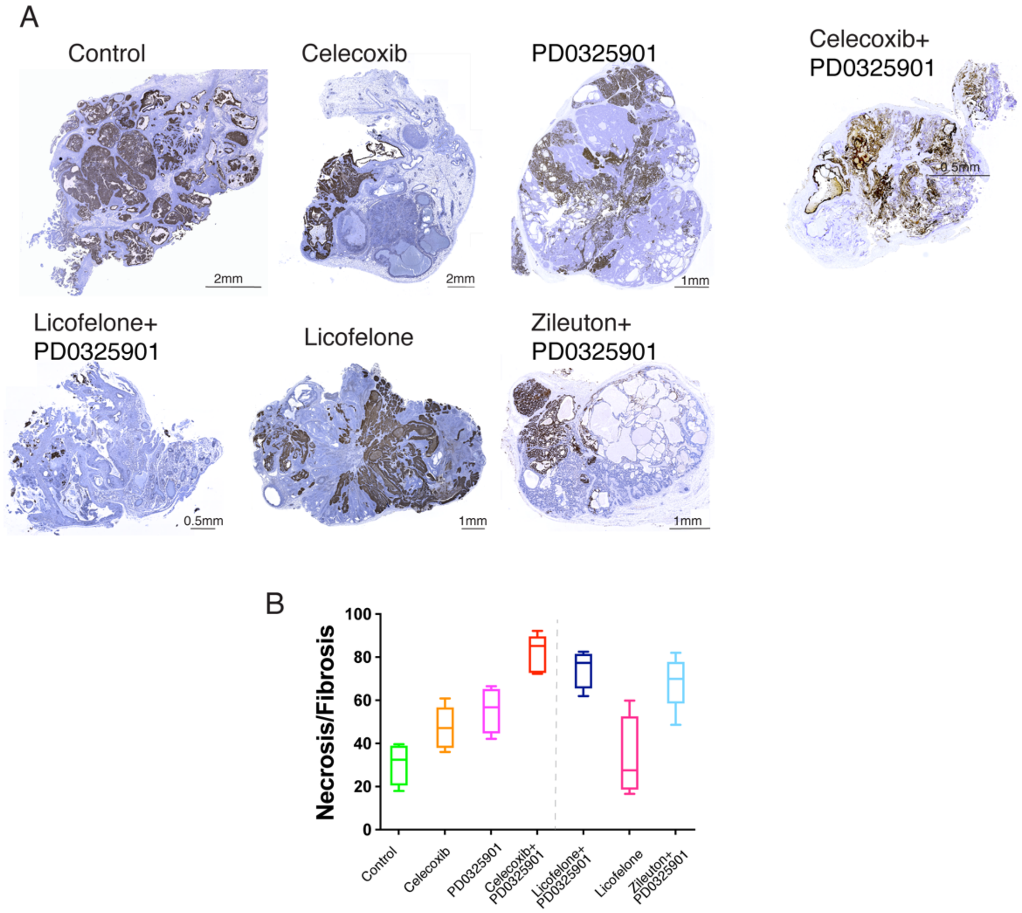
A
Control
Celecoxib
PD0325901
Celecoxib+
PD0325901
2mm
2mm
1mm
0.5mm
Licofelone+
PD0325901
Licofelone
Zileuton+
PD0325901
0.5mm
1mm
1mm
B
Necrosis/Fibrosis
100
80
60
40
20
0
Control
Celecoxib
PD0325901
Celecoxib+
PD0325901
Licofelone+
PD0325901
Licofelone
Zileuton+
PD0325901

C

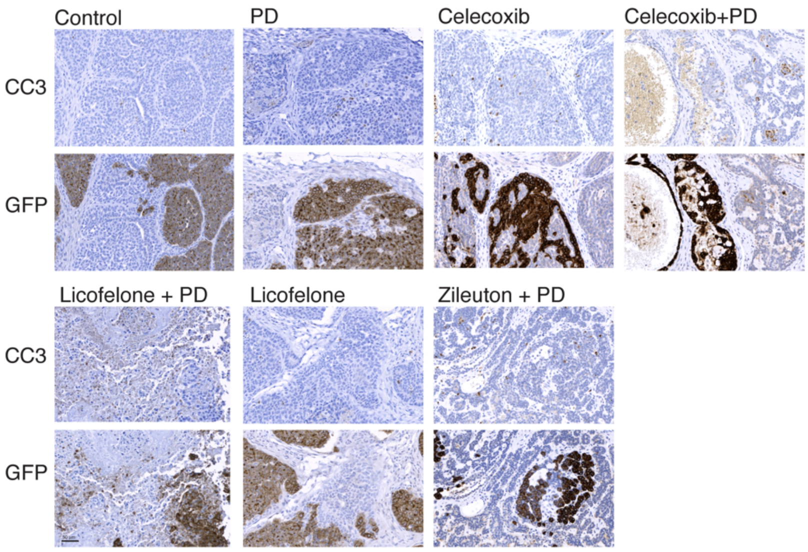
Control
PD
Celecoxib
Celecoxib+PD
CC3
GFP
Licofelone + PD
Licofelone
Zileuton + PD
CC3
GFP

D

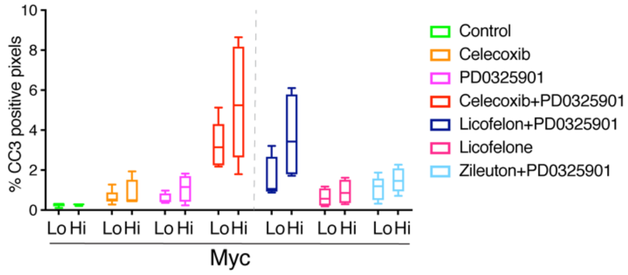
% CC3 positive pixels
0
2
4
6
8
10
Lo Hi Lo Hi Lo Hi Lo Hi Lo Hi Lo Hi Lo Hi
Myc
Control
Celecoxib
PD0325901
Celecoxib+PD0325901
Licofelon+PD0325901
Licofelone
Zileuton+PD0325901

E

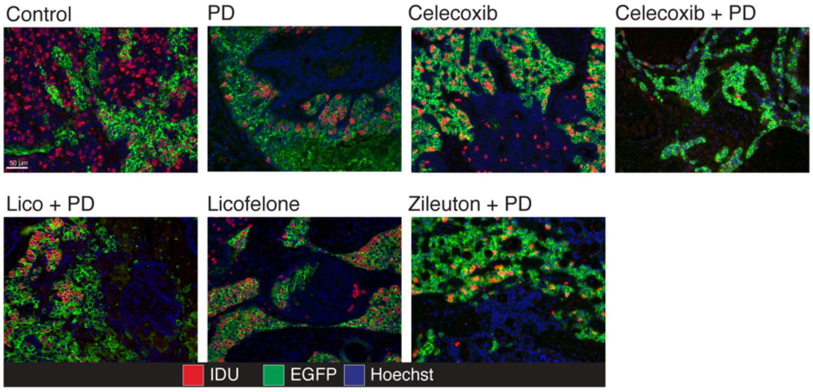
Control
PD
Celecoxib
Celecoxib + PD
Lico + PD
Licofelone
Zileuton + PD
IDU
EGFP
Hoechst

F

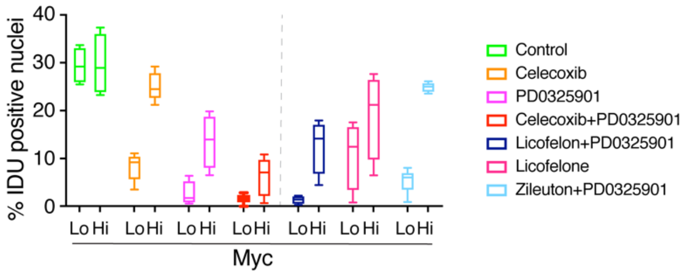
% IDU positive nuclei
0
10
20
30
40
Lo Hi Lo Hi Lo Hi Lo Hi Lo Hi Lo Hi Lo Hi
Myc
Control
Celecoxib
PD0325901
Celecoxib+PD0325901
Licofelon+PD0325901
Licofelone
Zileuton+PD0325901

Figure S10. Overviews and Quantifications showing the synergistic effect of MEK and COX inhibition in mammary tumours.

a) Representative full tumour scans and representative images of immunohistochemical DAB staining of mixed *WM⁺T/WM⁻T* tumours for cleaved Caspase 3 and representative images of immunofluorescent staining for 5-Iodo-2′-deoxyuridine (IDU). Related to Figure 7.
b) Representative full tumour scans of immunohistochemical DAB staining of mixed *WM⁺T/WM⁻ T* tumours for GFP to reveal *WM⁺T* clones, after three days of treatment with Tamoxifen (100 µg/mouse twice daily) followed by four days treatment with Tamoxifen and drug combinations (Celecoxib, 20 mg/kg/day, PD0325901 10 mg/kg/day, Licofelone 100 mg/kg/day, Zileuton 20 mg/kg/day), revealing the stark reduction in tumour cells by combination treatment.
c) Quantification of necrosis/fibrosis of mixed *WM⁺T/WM⁻T* tumours after three days of treatment with Tamoxifen (100 µg/mouse twice daily) followed by four days treatment with Tamoxifen and drug combinations (Celecoxib, 20 mg/kg/day, PD0325901 10 mg/kg/day, Licofelone 100 mg/kg/day, Zileuton 20 mg/kg/day; n: Control, PD0325901, Licofelone+ PD0325901, Licofelone = 4, Celecoxib, Celecoxib+ PD0325901, Zileuton = 5)
d) Representative images of immunohistochemical DAB staining of mixed *WM⁺T/WM⁻ T* tumours for cleaved Caspase 3, after three days of treatment with Tamoxifen (100 µg/mouse twice daily) followed by four days treatment with Tamoxifen and drug combinations (Celecoxib, 20 mg/kg/day, PD0325901 10 mg/kg/day, Licofelone 100 mg/kg/day, Zileuton 20 mg/kg/day; n: Control, PD0325901, Licofelone+ PD0325901, Licofelone = 4, Celecoxib, Celecoxib + PD0325901, Zileuton = 5)
e) Quantification of cell death in mixed *WM⁺T/ WM⁻T* tumours as percentage of CC3 positive pixels in the individual clones on 3 representative visual fields per mouse after three days of treatment with Tamoxifen followed by four days treatment with Tamoxifen and drug combinations (n: Control, PD0325901, Licofelone+ PD0325901, Licofelone = 4, Celecoxib, Celecoxib + PD0325901, Zileuton = 5)
f) Representative images of immunofluorescent staining for 5-Iodo-2′-deoxyuridine (IDU, red) injected two hours prior to sacrifice, EGFP as a marker for Cre-recombination (green) and DNA (blue), of mixed *WM⁺T/ WM⁻T* tumours after three days of treatment with Tamoxifen (100 µg/mouse twice daily) followed by four days treatment with Tamoxifen and drug combinations.
g) Quantification of proliferation in mixed *WM⁺T/ WM⁻T* tumours by automated counting of IDU positive nuclei over total nuclei in the individual clones on 3 representative visual fields per mouse after three days of treatment with Tamoxifen followed by four days treatment with Tamoxifen and drug combinations (n: Control, PD0325901, Licofelone+ PD0325901, Licofelone = 4, Celecoxib, Celecoxib+ PD0325901, Zileuton = 5)

Extended Methods

Tumour generation, propagation, and cell preparation

Rosa26-CAG-lox-STOP-lox-MycERT2/ Rosa26-mTmG/ MMTV-Wnt1 triple transgenic mice were allowed to develop spontaneous tumours (latency 6-10 months). Once tumours were palpable, mice were injected i.p. with Tamoxifen once daily to switch the resulting tumour to an ER negative state. Once tumours reached 1.5 mm^3, they were excised and cut into small fragments for cryopreservation in FBS with 10% DMSO. Fragments were subsequently orthotopically implanted into the uncleared #4 fat pad of SCID mice. Secondary tumours were treated with Tamoxifen once palpable and no growth attenuation was observed at this stage. Once transplanted tumours reached 1.5 mm^3, they were excised, finely minced, and digested for two hours in 5 ml additive-free DMEM with 1 mg/ml Collagen/Dispase (Roche). Red blood cells were lysed using standard protocols and cells cultured in DMEM with 10% FBS. To induce Cre recombination, Cre-expressing attenuated adenovirus (Ad5CMV-Cre, University of Iowa, VVC-U of Iowa-5) was added to the culture and the cells incubated overnight. Primary tumour cells were then flow sorted (SONY SH800S) based on GFP expression as an indicator for successful recombination. Cells were resuspended in additive free DMEM with 10% Matrigel, either as pure populations or as mixtures of recombined (green) and non-recombined (red) cells (mixtures were between 20%/80%-30%/70% of green/red cells). Between 90,000 and 250,000 cells were injected into the uncleared #4 fat pad of SCID mice to generate the experimental tumours.

Mouse husbandry and procedures

Mice were maintained on regular diet in a pathogen-free facility on a 12-hr light/dark cycle with continuous access to food and water. *R26CAG*$^{LSL\text{-}c\text{-}MycERT2}$ mice were created on the backbone of the previously described *R26*$^{LSL\text{-}c\text{-}MycERT2}$ backbone, adding a CAG enhancer sequence. Mice carrying *Rosa26-mTmG* (1), and the *MMTV-Wnt1* (2) were described previously. For activation of MycER^{T2}, Tamoxifen (Sigma; TS648) was dissolved in ethanol at 100 mg/ml and then further diluted in vegetable oil at a final concentration of 10 mg/ml. Mice were injected with 1 mg (100 µl)/20 g *i.p.* twice daily for three days. For long term studies, mice were injected with Tamoxifen as described above for 2 days, while also being transitioned onto Tamoxifen diet (manufacturer), to assure prompt activation of MycER^{T2}. Subsequently, they were kept on Tamoxifen diet until culling. Mice were checked daily for tumours and tumours were measured using callipers. Mice received 1 mg/20 g of IDU (stock 10 mg/ml, manufacturer) *i.p.* 2 hours prior to culling via cervical dislocation.

Analysis of normal mammary gland

Mice carrying the embryonically expressed *Zp3-Cre* construct were crossed to mice carrying the *Rosa26-CAG-lox-STOP-lox-MycER*^{T2} allele. Myc was activated systemically for 8 hours in 8-10 week old mice by a single *i.p* injection of 1 mg of Tamoxifen, as described above. Mice were culled and the lymph node-divested number 4 mammary glands were collected and snap frozen. RNA was extracted using Trizol reagent following the manufacturer's instructions.

Immunohistochemistry and Immunofluorescence

Mice were culled via cervical dislocation, mammary tumours were removed and, in the case of mixed Myc^{high}/Myc^{low} tumours, tumours were cross-sectioned through areas of clonal intermingling using goggles fitted with a UV source and fluorescent filters (BLS). Tumours were fixed overnight in neutral-buffered formalin (Sigma-Aldrich, 501320) and processed for paraffin embedding. Tissue sections were stained with haematoxylin and eosin (H&E) using standard reagents and protocols. For frozen sections, tumours were briefly fixed in fresh 10% paraformaldehyde in PBS, transferred to a 30% sucrose solution and subsequently embedded in OCT (VWR Chemicals, 361603E) and stored at -80 °C.

For analysis, sections were de-paraffinized, rehydrated, and boiled in a microwave for 10 minutes in 10 mM citrate buffer (pH 6.0) to retrieve antigens. Tissue sections were blocked in

PBS containing 1.5% normal goat serum (NGS) and 2% bovine serum albumin (BSA). Primary antibodies were added overnight at 4 °C. For immunohistochemistry, HRP-linked secondary antibodies (Vectastain Elite ABC Kits: PK-6101; Rabbit, PK-6104; Rat) were added for 60 minutes at room temperature, slides were washed and visualized binding visualized with DAB (Vector Laboratories; SK-4100). Slides were then dehydrated, counterstained and mounted in DPX (Fisher Scientific, 12658646) following standard protocols. For immunofluorescence, Alexa Fluor 488, -555, or 648 dye-conjugated antibodies (Life Technologies), were added to sections for 1 hours at room temperature. Nuclei were visualised with Hoechst 33258 (Sigma, 861405, 0.5 μg/ml) and mounted in fluorescent mounting medium (Prolong Molecular Probes; P36934). Primary antibodies used were as follows: HIF1α - SC10790, 1:50; CD31 - ab28364, 1:100; Cleaved Caspase 3 - cs9664, 1:1000; oestrogen receptor alpha - SC-542, 1:50; IDU – BD347580, 1:100; $p19^{ARF}$ - sc-32748, 1:100, β-catenin - BD610153, 1:250, Myc - ab32072, 1:1000; GFP - ab6556, 1:500; p53 – Leica CM5p, 1:500. Pictures were acquired on a Zeiss Axio Imager M2 microscope and Axiovision Rel 4.8 software. For DAB staining, white balance was adjusted using Adobe Photoshop.

Quantification of Cellularity and Necrosis
Tumour cellularity was quantified by counting Hoechst counter-stained nuclei in three representative fields of vision per analysed tumour. Necrosis was assessed by histological analysis of whole slide scans of H&E stained tumour sections. The quantification was performed by manually measuring necrotic areas using the image analysis software ImageJ.

Quantification of blood vessel size
The average area of blood vessels was assessed by tracing all blood vessels, visualised via CD31 immunohistochemical DAB staining, in a minimum of three representative sections per mouse, using the ImageJ image analysis software, and calculating the average area of the vessels' cross section per visual field.

Quantification of Hypoxia
The extent of hypoxia was quantified by immunohistochemically staining tumour sections for HIF1α. Using the image analysis software ImageJ, the DAB staining and the nuclear Haematoxylin staining was deconvoluted using the colour deconvolution algorithm. DAB (HIFα) positive nuclei and total nuclei were subject to automated counting and the ratio of the two was taken as a measure of hypoxia.

Tissue culture
67NR cell lines were cultures in DMEM (Gibco) with 10% FBS. The pTREIPZ-TRE-Myc-SV40-Turbo-RFP construct was stably introduced using Lipofectamine 3000 (Invitrogen) and selected for with Puromycin at a final concentration of 7.5 μg/ml. To activate Myc cells were stimulated for three days with Doxycycline (final concentration 1 μg/ml), which was replaced every 2 days. For RNA extraction cells were washed and collected in Trizol reagent following the manufacturer's instructions. For flow cytometry cells were detached using trypsin, washed and resuspended in PBS, 1% BSA. Cells were stained with 7-AAD as a viability stain and analysed on a BD Accuri C6 analyser. By growing L-cells stably infected with a construct expressing Wnt3a (ATCC® CRL-2647™) and harvesting the supernatant we generated Wnt3a conditioned media (L3-CM). Control media (L-CM) was generated by growing the parental cell line (L-Cells, ATCC® CRL-2648™) and harvesting the supernatant.

qRT-Pcr and ddPCR primers
qPCR primers:
GAPDH: TGTAGACCATGTAGTTGAGGTCA - AGGTCGGTGTGAACGGATTTG
Puma: AGCAGCACTTAGAGTCGCC - CCTGGGTAAGGGGAGGAGT
Noxa: GCAGAGCTACCACCTGAGT – CTTTTGCGACTTCCCAGGCA
INK4a-ARF: GGGTTTTCTTGGTGAAGTTCG – TTGCCCATCATCATCACCT

p21^{cip1}*/CDKN1A*: AACATCTCAGGGCCGAA - TGCGCTTGGAGTGATAGAAA
Bim: GTGGGTATTTCTCTTTTGACACAGAC – GTTCAGCCTGCCTCATGGAAG
Wnt1: GGTTTCTACTACGTTGCTACTGG – GGAATCCGTCAACAGGTTCGT
Axin2: AAGAGAAGCGACCCAGTCA – CTGCGATGCATCTCTCTCTG

Digital Droplet PCR primers and probes for genomic analysis:
Primer forward: CGACGGTATCGTAGAGTCGAG
Primer reverse unrecombined: CAAGCTTATCGATACCGTCGAGG
Primer reverse recombined: GAAGCTAACGTTGAGGGGC
Probe unrecombined: [6FAM]TCCCCTACCCGGTAGAATTCCTGCA[BHQ1]
Probe recombined: [HEX]TATCGAATTCGAGCTCGCCCC[BHQ1]

Tumour therapeutic studies drug regime
Licofelone, potassium salt (Calbiochem #435801) was freshly suspended in sunflower oil at a concentration of 20 mg/ml and administered by oral gavage at a dose of 100 mg/kg/day. PD0325901 (Selleckchem #S1036) Celecoxib (Generon Ltd) and Zileuton (Generon Ltd) was stored in DMSO at 20 mg/ml as a stock solution. This working solution was diluted to 2 mg/ml in water together with 0.5% final concentration Hydroxyethyl-cellulose as thickener, and 0.2% Tween and administered at 10 mg/kg/day via oral gavage. 2 hours prior to culling, mice received 1 mg/20 g of IDU (stock 10 mg/ml) i.p. Mice were culled via cervical dislocation.

Using Qualitative Networks to model genetic and molecular networks
In a Qualitative Network, nodes can take many values rather than 0 or 1. These values must be natural numbers, hence Qualitative, and within a fixed range. Nodes can be of different ranges, henceforth referred to as the node granularity. At any time point every node will have a level of activity within this range.
Most nodes in the model vary from 0-2, representing low, normal and high activity. Phenotypes, and key nodes feeding into them, have a higher range up to 0-6.
Nodes are connected by edges. These can either be activating or inhibitory. This only affects default target functions, for any manual target function edges only pass the state of a node.
When passing values between nodes of unequal granularity, the value is converted to scale to the new granularity.
If node X has a granularity a-b, and it appears in the target function of Y whose granularity is *c-d,* the value of X is converted to:

$$\frac{(X-a)(d-c)}{(b-a)+c}$$

The network updates synchronously according to the Target Functions of the nodes, in increments of one unit of activity. This means that each node will examine the activity of nodes connected to it with incoming edges and evaluate them according to its target function. It will then adjust its activity by one in the direction of the Target Function output. This is described formally in (3).

Finding and classifying network attractors
The network is modelled as a discrete system, thus there is a finite number of states it can take, and from every initial state it must enter into a loop of some finite length n, **an attractor**. BMA can be used to characterise such behaviour. If the system tends towards a loop n = 1, i.e. no change between each time step, we say it stabilises. If it reaches a loop of length n > 1 we call this an oscillation. We assess stabilisation for all initial states, which introduces a third case that there are different possible loops for different initial states, which we refer to as bifurcation. BMA provides two methods for such assessment. First a fast algorithm that either finds a stable state, or, failing to do so finds, a range for each of the nodes that contain all loops. Second, a thorough but potentially slower algorithm that uses an SMT solver to discriminate between the three options above. However, the SMT solver may take an unknown and large

amount of time to find a solution. Thus, this second algorithm is unsuitable to testing large numbers of combinations of perturbation. Both methods are described further in (4).

Building and testing the computational model

The network model was built and tested using a wide range of existing experimental evidence, drawn from the literature. Literature supporting node interactions is collated in Dataset S1. A second set of experiments were used to test overall cellular behaviour, such as the response of cell lines to drugs under different conditions (see Dataset S3). The results of these experiments were dependent upon the crosstalk between pathways, so could not be directly encoded into the model. As such these were used to test model behaviour and refine the target functions (Dataset S2) and how we map a continuous system onto a discrete model. We also compared the activity of a panel of nodes in the model, under conditions mimicking the tumours composed of Myc^{low} or Myc^{high} cells alone, to the measured the activity in the mouse model of the genes and proteins these nodes represented (see Dataset S4). In this way we validated the network model before using it to predict the effects of perturbations on the bi-clonal tumours. We made our *in silico* model specific to our mouse model of breast cancer through the choice of which pathways to include, namely Wnt and EGF signalling feeding into a response by the Ras and Myc pathways, regulated by the p53 tumour suppressor. We also obtained the data to support the edges and target functions and the tests of correct model behaviour from experiments done on breast cancer cell lines, where possible. The cell type used in each supporting experiment is noted in Dataset S1.

Reproducing experimental conditions in the network model

To simulate an experimental perturbation of the cells in the model, the values of nodes representing mutations, as well as external factors such as EGF, were first fixed to a constant value by changing the Target Function, e.g. to be *const(2)*. These are shown in Dataset S3 for the cell lines against which we compared the model. Similarly, adjustments to the target functions in order to simulate the monoclonal and bi-clonal Myc^{low} and Myc^{high} tumours in the mouse model are shown in Dataset S5. We simulate the bi-clonal case for each clone separately, with changes to the target functions to represent the microenvironmental conditions resulting from mixing of the clones. For example, when simulating the Myc^{high} clone in the mixed tumour, the availability of Wnt1 provided by Myc^{low} cells is modelled, in contrast to the monoclonal case. Adjustments made to simulate any additional experimental perturbations, such as a drug treatment, are further superimposed over this. The stable state of the model was then compared to experiments.
This panel of experiments thus forms a specification for what behaviour we are able to model, defining its scope. The output of the model is compared to the experimental data, and in case of a mismatch iterated by changing the Target Functions, or searching for missing edges, until it was able to match all experiments drawn from the literature. In order to simulate an experiment using our model, we need to set the initial conditions of the model to match the mutational oncogene and tumour suppressor profile of the tumour cells. We therefore require an exact mutational profile of the cells. As such, for the validation of the model we focussed on cell lines that had been mutationally profiled. While these are not completely stable, they have been studied to find instances of mutations, amplifications and copy number variations that translate into differences of node activity in the model, which would not be feasible with primary tissue data.

Dataset Captions

All references in these datasets refer to those below in this Supplementary Information Appendix.

Dataset S1 Experimental evidence for executable model edges, see also Figure 4, Materials and Methods.

Dataset S2 Target functions in the executable model, see also Figure 4, Materials and Methods.

Dataset S3 In vitro experiments used to verify the executable model, see also Materials and Methods.

Dataset S4 In vivo experiments used to verify the executable model, see also Materials and Methods.

Dataset S5 Background conditions used to reproduce different tumour types in the executable model, see also Figure 5, SI Appendix Figure S5-9 and Materials and Methods.

Dataset S6 Genes represented by nodes in the executable model, see also Figure 4 and Materials and Methods.

Dataset S7 Pathways of nodes in the executable model, see also Figure 5, SI Appendix Figures S5-9 and Materials and Methods.

References

1. Muzumdar MD, Tasic B, Miyamichi K, Li L, Luo L (2007) A global double-fluorescent Cre reporter mouse. *genesis* 45(9):593–605.
2. Tsukamoto AS, Grosschedl R, Guzman RC, Parslow T, Varmus HE (1988) Expression of the int-1 gene in transgenic mice is associated with mammary gland hyperplasia and adenocarcinomas in male and female mice. *Cell* 55(4):619–625.
3. Schaub MA, Henzinger TA, Fisher J (2007) Qualitative networks: a symbolic approach to analyze biological signaling networks. *BMC Syst Biol* 1:4.
4. Cook B, Fisher J, Krepska E, Piterman N (2011) Proving Stabilization of Biological Systems. *VMCAI* (Springer), pp 134–149.
5. Berns K, et al. (2007) A Functional Genetic Approach Identifies the PI3K Pathway as a Major Determinant of Trastuzumab Resistance in Breast Cancer. *Cancer Cell* 12(4):395–402.
6. Blagosklonny M V, El-Deiry WS (1996) In vitro evaluation of a p53-expressing adenovirus as an anti-cancer drug. *Int J cancer* 67(3):386–92.
7. Hegde PS, et al. (2007) Delineation of molecular mechanisms of sensitivity to lapatinib in breast cancer cell lines using global gene expression profiles. *Mol Cancer Ther* 6(5):1629–40.
8. Kao J, et al. (2009) Molecular profiling of breast cancer cell lines defines relevant tumor models and provides a resource for cancer gene discovery. *PLoS One* 4(7). doi:10.1371/journal.pone.0006146.
9. Hollestelle A, et al. (2010) Distinct gene mutation profiles among luminal-type and basal-type breast cancer cell lines. *Breast Cancer Res Treat* 121(1):53–64.
10. Matsuda Y, Schlange T, Oakeley EJ, Boulay A, Hynes NE (2009) WNT signaling enhances breast cancer cell motility and blockade of the WNT pathway by sFRP1 suppresses MDA-MB-231 xenograft growth. *Breast Cancer Res* 11(3):R32.
11. Michalak EM, Villunger A, Adams JM, Strasser A (2008) In several cell types tumour suppressor p53 induces apoptosis largely via Puma but Noxa can contribute. *Cell Death Differ* 15(6):1019–1029.
12. Petit AM, et al. (1997) Neutralizing antibodies against epidermal growth factor and ErbB-2/neu receptor tyrosine kinases down-regulate vascular endothelial growth factor production by tumor cells in vitro and in vivo: angiogenic implications for signal transduction therapy of so. *Am J Pathol* 151(6):1523–30.
13. Scott PA, Gleadle JM, Bicknell R, Harris AL (1998) Role of the hypoxia sensing system, acidity and reproductive hormones in the variability of vascular endothelial growth factor induction in human breast carcinoma cell lines. *Int J cancer* 75(5):706–12.
14. Sinn E, et al. (1987) Coexpression of MMTV/v-Ha-ras and MMTV/c-myc genes in transgenic mice: Synergistic action of oncogenes in vivo. *Cell* 49(4):465–475.
15. She Q-B, et al. (2008) Breast tumor cells with PI3K mutation or HER2 amplification are selectively addicted to Akt signaling. *PLoS One* 3(8):e3065.
16. Vranic S, Gatalica Z, Wang ZY (2011) Update on the molecular profile of the MDA-MB-453 cell line as a model for apocrine breast carcinoma studies. *Oncol Lett* 2(6):1131–1137.
17. Watson PH, Pon RT, Shiu RP (1991) Inhibition of c-myc expression by

phosphorothioate antisense oligonucleotide identifies a critical role for c-myc in the growth of human breast cancer. *Cancer Res* 51(15):3996–4000.

18. Al-azawi D, et al. (2008) Ets-2 and p160 proteins collaborate to regulate c-Myc in endocrine resistant breast cancer. *Oncogene* 27(21):3021–31.

19. Amati B, Alevizopoulos K, Vlach J (1998) Myc and the cell cycle. *Front Biosci* 3:d250-68.

20. An WG, et al. (1998) Stabilization of wild-type p53 by hypoxia-inducible factor 1alpha. *Nature* 392(6674):405–408.

21. Aplin AE, Stewart SA, Assoian RK, Juliano RL (2001) Integrin-mediated adhesion regulates ERK nuclear translocation and phosphorylation of Elk-1. *J Cell Biol* 153(2):273–281.

22. Bates S, et al. (1998) p14ARF links the tumour suppressors RB and p53. *Nature* 395(September):124–125.

23. Behrens J, et al. (1996) Functional Interaction of β-Catenin with the Transcription Factor LEF-1. *Nature* 382(6592):638–642.

24. Berra E, et al. (2003) HIF prolyl-hydroxylase 2 is the key oxygen sensor setting low steady-state levels of HIF-1α in normoxia. *EMBO J* 22(16):4082–4090.

25. Beverly LJ, Varmus HE (2009) MYC-induced myeloid leukemogenesis is accelerated by all six members of the antiapoptotic BCL family. *Oncogene* 28(9):1274–9.

26. Bhanot P, et al. (1996) A new member of the frizzled family from Drosophila functions as a Wingless receptor. *Nature* 382(6588):225–230.

27. Bilic J, et al. (2007) Wnt induces LRP6 signalosomes and promotes dishevelled-dependent LRP6 phosphorylation. *Science* 316(5831):1619–1622.

28. Billen LP, Kokoski CL, Lovell JF, Leber B, Andrews DW (2008) Bcl-XL Inhibits Membrane Permeabilization by Competing with Bax. *PLoS Biol* 6(6):e147.

29. Blagosklonny M V, et al. (1998) p53 inhibits hypoxia-inducible factor-stimulated transcription. *J Biol Chem* 273(20):11995–8.

30. Blancher C, Moore JW, Robertson N, Harris AL (2001) Effects of ras and von Hippel-Lindau (VHL) gene mutations on hypoxia-inducible factor (HIF)-1alpha, HIF-2alpha, and vascular endothelial growth factor expression and their regulation by the phosphatidylinositol 3'-kinase/Akt signaling pathway. *Cancer Res* 61(19):7349–7355.

31. Blaydes JP, Wynford-Thomas D (1998) The proliferation of normal human fibroblasts is dependent upon negative regulation of p53 function by mdm2. *Oncogene* 16(January):3317–3322.

32. Boras-Granic K, Wysolmerski JJ (2008) Wnt signaling in breast organogenesis. *Organogenesis* 4(2):116–122.

33. Bunz F, et al. (1998) Requirement for p53 and p21 to sustain G2 arrest after DNA damage. *Science* 282(April):1497–1501.

34. Campone M, et al. (2008) Prediction of metastatic relapse in node-positive breast cancer: Establishment of a clinicogenomic model after FEC100 adjuvant regimen. *Breast Cancer Res Treat* 109(3):491–501.

35. Campone M, et al. (2011) c-Myc dependent expression of pro-apoptotic Bim renders HER2-overexpressing breast cancer cells dependent on anti-apoptotic Mcl-1. *Mol Cancer* 10(1):110.

36. Capuco A V, et al. (2002) Concurrent pregnancy retards mammary involution: effects on apoptosis and proliferation of the mammary epithelium after forced weaning of

mice. *Biol Reprod* 66(5):1471–6.

37. Cardone MH (1998) Regulation of Cell Death Protease Caspase-9 by Phosphorylation. *Science (80-)* 282(5392):1318–1321.

38. Castelo-Branco G, et al. (2003) Differential regulation of midbrain dopaminergic neuron development by Wnt-1, Wnt-3a, and Wnt-5a. *Proc Natl Acad Sci U S A* 100(22):12747–12752.

39. Chen L, et al. (2005) Differential Targeting of Prosurvival Bcl-2 Proteins by Their BH3-Only Ligands Allows Complementary Apoptotic Function. *Mol Cell* 17(3):393–403.

40. Cheng M, Sexl V, Sherr CJ, Roussel MF (1998) Assembly of cyclin D-dependent kinase and titration of p27Kip1 regulated by mitogen-activated protein kinase kinase (MEK1). *Proc Natl Acad Sci U S A* 95(3):1091–6.

41. Cobb MH (1999) MAP kinase pathways. *Prog Biophys Mol Biol* 71(3–4):479–500.

42. Coller HA, et al. (2000) Expression analysis with oligonucleotide microarrays reveals that MYC regulates genes involved in growth, cell cycle, signaling, and adhesion. *Proc Natl Acad Sci U S A* 97(7):3260–5.

43. Collins NL, et al. (2005) G1/S cell cycle arrest provides anoikis resistance through Erk-mediated Bim suppression. *Mol Cell Biol* 25(12):5282–91.

44. Chen D, Li M, Luo J, Gu W (2003) Direct interactions between HIF-1 alpha and Mdm2 modulate p53 function. *J Biol Chem* 278(16):13595–8.

45. Dewson G, et al. (2008) To trigger apoptosis, Bak exposes its BH3 domain and homodimerizes via BH3:groove interactions. *Mol Cell* 30(3):369–80.

46. Diehl JA, Cheng M, Roussel MF, Sherr CJ (1998) Glycogen synthase kinase-3beta regulates cyclin D1 proteolysis and subcellular localization. *Genes Dev* 12(22):3499–3511.

47. Dijkers PF, et al. (2000) Forkhead transcription factor FKHR-L1 modulates cytokine-dependent transcriptional regulation of p27(KIP1). *Mol Cell Biol* 20(24):9138–9148.

48. Ding Q, et al. (2005) Erk associates with and primes GSK-3β for its inactivation resulting in upregulation of β-catenin. *Mol Cell* 19(2):159–170.

49. Doehn U, et al. (2009) RSK is a principal effector of the RAS-ERK pathway for eliciting a coordinate promotile/invasive gene program and phenotype in epithelial cells. *Mol Cell* 35(4):511–22.

50. Dong A, Wodziak D, Lowe AW (2015) Epidermal growth factor receptor (EGFR) signaling requires a specific endoplasmic reticulum thioredoxin for the post-translational control of receptor presentation to the cell surface. *J Biol Chem* 290(13):8016–8027.

51. Draetta GF (1994) Mammalian G1 cyclins. *Curr Opin Cell Biol* 6(6):842–846.

52. Eldar-Finkelman H, Seger R, Vandenheede JR, Krebs EG (1995) Inactivation of glycogen synthase kinase-3 by epidermal growth factor is mediated by mitogen-activated protein kinase/p90 ribosomal protein S6 kinase signaling pathway in NIH/3T3 cells. *J Biol Chem* 270(3):987–990.

53. Esteva FJ, et al. (2001) Expression of erbB/HER receptors, heregulin and P38 in primary breast cancer using quantitative immunohistochemistry. *Pathol Oncol Res* 7(3):171–7.

54. Fanale D, et al. (2013) HIF-1 is involved in the negative regulation of AURKA expression in breast cancer cell lines under hypoxic conditions. *Breast Cancer Res*

Treat:1–13.

55. Farago M, et al. (2005) Kinase-inactive glycogen synthase kinase 3beta promotes Wnt signaling and mammary tumorigenesis. *Cancer Res* 65(13):5792–801.

56. Feng XH, Liang YY, Liang M, Zhai W, Lin X (2002) Direct interaction of c-Myc with Smad2 and Smad3 to inhibit TGF-β-mediated induction of the CDK inhibitor p15Ink4B. *Mol Cell* 9(1):133–143.

57. Fernandez-Pol JA, Talkad VD, Klos DJ, Hamilton PD (1987) Suppression of the EGF-dependent induction of c-myc proto-oncogene expression by transforming growth factor beta in a human breast carcinoma cell line. *Biochem Biophys Res Commun* 144(3):1197–205.

58. Franke TF, Kaplan DR, Cantley LC (1997) PI3K: downstream AKTion blocks apoptosis. *Cell* 88(4):435–7.

59. Grandori C, Cowley SM, James LP, Eisenman RN (2000) The Myc/Max/Mad network and the transcriptional control of cell behavior. *Annu Rev Cell Dev Biol* 16:653–99.

60. Germain M, Milburn J, Duronio V (2008) MCL-1 inhibits BAX in the absence of MCL-1/BAX Interaction. *J Biol Chem* 283(10):6384–92.

61. Gibson L, et al. (1996) bcl-w, a novel member of the bcl-2 family, promotes cell survival. *Oncogene* 13(4):665–75.

62. Gilmore AP, et al. (2002) Activation of BAD by therapeutic inhibition of epidermal growth factor receptor and transactivation by insulin-like growth factor receptor. *J Biol Chem* 277(31):27643–50.

63. Goel S, et al. (2012) Both LRP5 and LRP6 receptors are required to respond to physiological Wnt ligands in mammary epithelial cells and fibroblasts. *J Biol Chem* 287(20):16454–16466.

64. Greijer AE, van der Wall E (2004) The role of hypoxia inducible factor 1 (HIF-1) in hypoxia induced apoptosis. *J Clin Pathol* 57(10):1009–14.

65. He TC, et al. (1998) Identification of c-MYC as a target of the APC pathway. *Science (80-)* 281(5382):1509–1512.

66. He G, et al. (2005) Induction of p21 by p53 following DNA damage inhibits both Cdk4 and Cdk2 activities. *Oncogene* 24(18):2929–43.

67. Hermeking H, et al. (2000) Identification of CDK4 as a target of c-MYC. *Proc Natl Acad Sci U S A* 97(5):2229–34.

68. Hill CS, Treisman R (1995) Differential activation of c-fos promoter elements by serum, lysophosphatidic acid, G proteins and polypeptide growth factors. *EMBO J* 14(20):5037–5047.

69. Hinck AP (2012) Structural studies of the TGF-βs and their receptors - insights into evolution of the TGF-β superfamily. *FEBS Lett* 586(14):1860–70.

70. Hoeben ANN, et al. (2004) Vascular endothelial growth factor and angiogenesis. *Pharmacol Rev* 56(4):549–580.

71. Holbro T, et al. (2003) The ErbB2/ErbB3 heterodimer functions as an oncogenic unit: ErbB2 requires ErbB3 to drive breast tumor cell proliferation. *Proc Natl Acad Sci U S A* 100(15):8933–8.

72. Howe LR, et al. (2001) PEA3 is up-regulated in response to Wnt1 and activates the expression of cyclooxygenase-2. *J Biol Chem* 276(23):20108–15.

73. Howe LR, Watanabe O, Leonard J, Brown AMC (2003) Twist is up-regulated in

response to Wnt1 and inhibits mouse mammary cell differentiation. *Cancer Res* 63(8):1906–1913.

74. Jelinek T, et al. (1994) RAS and RAF-1 form a signalling complex with MEK-1 but not MEK-2. *Mol Cell Biol* 14(12):8212–8.

75. Lane HA, Motoyama AB, Beuvink I, Hynes NE (2001) Modulation of p27/Cdk2 complex formation through 4D5-mediated inhibition of HER2 receptor signaling. *Ann Oncol Off J Eur Soc Med Oncol* 12 Suppl 1(Supplement 8):S21-2.

76. Kaur M, Cole MD (2013) MYC acts via the PTEN tumor suppressor to elicit autoregulation and genome-wide gene repression by activation of the Ezh2 methyltransferase. *Cancer Res* 73(2):695–705.

77. Kim MS, Lee EJ, Kim HRC, Moon A (2003) p38 kinase is a key signaling molecule for H-ras-induced cell motility and invasive phenotype in human breast epithelial cells. *Cancer Res* 63(17):5454–5461.

78. Kim H, et al. (2016) Tumor necrosis factor receptor-associated protein 1 (TRAP1) mutation and TRAP1 inhibitor gamitrinibtriphenylphosphonium (G-TPP) induce a forkhead box O (FOXO)-dependent cell protective signal from mitochondria. *J Biol Chem* 291(4):1841–1853.

79. Klapper LN, et al. (1999) The ErbB-2/HER2 oncoprotein of human carcinomas may function solely as a shared coreceptor for multiple stroma-derived growth factors. *Proc Natl Acad Sci U S A* 96(9):4995–5000.

80. Knuefermann C, et al. (2003) HER2/PI-3K/Akt activation leads to a multidrug resistance in human breast adenocarcinoma cells. *Oncogene* 22:3205–3212.

81. Kong W, et al. (2014) Upregulation of miRNA-155 promotes tumour angiogenesis by targeting VHL and is associated with poor prognosis and triple-negative breast cancer. *Oncogene* 33(November 2012):679–89.

82. Korinek V, et al. (1997) Constitutive transcriptional activation by a b-catenin-Tcf complex in APC-/- colon carcinoma. *Science (80-)* 275(March):1784–1787.

83. Koshiji M, et al. (2004) HIF-1alpha induces cell cycle arrest by functionally counteracting Myc. *EMBO J* 23(9):1949–56.

84. Kundu N, Yang Q, Dorsey R, Fulton AM (2001) Increased cyclooxygenase-2 (cox-2) expression and activity in a murine model of metastatic breast cancer. *Int J Cancer* 93(5):681–6.

85. Kwon YS, Chun SY, Nam KS, Kim S (2015) Lapatinib sensitizes quiescent MDA-MB-231 breast cancer cells to doxorubicin by inhibiting the expression of multidrug resistance-associated protein-1. *Oncol Rep* 34(2):884–890.

86. Kyriakis JM, et al. (1992) Raf-1 activates MAP kinase-kinase. *Nature* 358(6385):417–21.

87. Laughner E, Taghavi P, Chiles K, Mahon PC, Semenza GL (2001) HER2 (neu) signaling increases the rate of hypoxia-inducible factor 1alpha (HIF-1alpha) synthesis: novel mechanism for HIF-1-mediated vascular endothelial growth factor expression. *Mol Cell Biol* 21(12):3995–4004.

88. Li Y, et al. (2006) Resveratrol-induced cell inhibition of growth and apoptosis in MCF7 human breast cancer cells are associated with modulation of phosphorylated Akt and caspase-9. *Appl Biochem Biotechnol* 135(3):181–192.

89. Lin SY, et al. (2000) Beta-catenin, a novel prognostic marker for breast cancer: its roles in cyclin D1 expression and cancer progression. *Proc Natl Acad Sci U S A* 97(8):4262–4266.

90. Low-Nam ST, et al. (2011) ErbB1 dimerization is promoted by domain co-confinement and stabilized by ligand binding. *Nat Struct Mol Biol* 18(11):1244–1249.

91. Lu C, et al. (2005) cFos is critical for MCF-7 breast cancer cell growth. *Oncogene* 24(43):6516–24.

92. Lukas J, et al. (1995) Retinoblastoma-protein-dependent cell-cycle inhibition by the tumour suppressor p16. *Nature* 375(6531):503–506.

93. MacDonald BT, He X (2012) Frizzled and LRP5/6 receptors for Wnt/β-catenin signaling. *Cold Spring Harb Perspect Biol* 4(12):a007880–a007880.

94. Mack FA, Patel JH, Biju MP, Haase VH, Simon MC (2005) Decreased growth of Vhl-/- fibrosarcomas is associated with elevated levels of cyclin kinase inhibitors p21 and p27. *Mol Cell Biol* 25(11):4565–78.

95. Maestro R, et al. (1999) Twist Is a Potential Oncogene That Inhibits Apoptosis. *Genes Dev* 13(17):2207–2217.

96. Majumder M, et al. (2016) COX-2 Induces Breast Cancer Stem Cells via EP4/PI3K/AKT/NOTCH/WNT Axis. *Stem Cells*. doi:10.1002/stem.2426.

97. McGlynn LM, et al. (2009) Ras/Raf-1/MAPK pathway mediates response to tamoxifen but not chemotherapy in breast cancer patients. *Clin Cancer Res* 15(4):1487–1495.

98. Mellor HR, Harris AL (2007) The role of the hypoxia-inducible BH3-only proteins BNIP3 and BNIP3L in cancer. *Cancer Metastasis Rev* 26(3–4):553–566.

99. Mitchell KO, El-Deiry WS (1999) Overexpression of c-Myc inhibits p21WAF1/CIP1 expression and induces S-phase entry in 12-O-tetradecanoylphorbol-13-acetate (TPA)-sensitive human cancer cells. *Cell Growth Differ* 10(4):223–30.

100. Morris M, Hepburn P, Wynford-Thomas D (2002) Sequential extension of proliferative lifespan in human fibroblasts induced by over-expression of CDK4 or 6 and loss of p53 function. *Oncogene* 21(27):4277–88.

101. Muthalagu N, et al. (2014) BIM Is the Primary Mediator of MYC-Induced Apoptosis in Multiple Solid Tissues. *Cell Rep* 8(5):1347–53.

102. Nakano K, Vousden KH (2001) PUMA, a Novel Proapoptotic Gene, Is Induced by p53. *Mol Cell* 7(3):683–694.

103. Nass SJ, Dickson RB (1998) Epidermal growth factor-dependent cell cycle progression is altered in mammary epithelial cells that overexpress c-myc. *Clin Cancer Res* 4(7):1813 LP – 1822.

104. Nikiforov MA, et al. (2007) Tumor cell-selective regulation of NOXA by c-MYC in response to proteasome inhibition. *Proc Natl Acad Sci U S A* 104(49):19488–93.

105. Ohtani N, et al. (2001) Opposing effects of Ets and Id proteins on p16INK4a expression during cellular senescence. *Nature* 409(6823):1067–1070.

106. Oltvai ZN, Milliman CL, Korsmeyer SJ (1993) Bcl-2 heterodimerizes in vivo with a conserved homolog, Bax, that accelerates programed cell death. *Cell* 74(4):609–619.

107. Pacold ME, et al. (2000) Crystal Structure and Functional Analysis of Ras Binding to Its Effector Phosphoinositide 3-Kinase γ. *Cell* 103(6):931–944.

108. Puisieux A, Valsesia-Wittmann S, Ansieau S (2006) A twist for survival and cancer progression. *Br J Cancer* 94:13–17.

109. Ray A, James MK, Larochelle S, Fisher RP, Blain SW (2009) p27Kip1 inhibits cyclin D-cyclin-dependent kinase 4 by two independent modes. *Mol Cell Biol* 29(4):986–999.

110. Reader J, Holt D, Fulton A (2011) Prostaglandin E2 EP receptors as therapeutic targets in breast cancer. *Cancer Metastasis Rev* 30(3–4):449–63.

111. Rimerman RA, Gellert-Randleman A, Diehl JA (2000) Wnt1 and MEK1 cooperate to promote cyclin D1 accumulation and cellular transformation. *J Biol Chem* 275(19):14736–14742.

112. Rodrik V, Gomes E, Hui L, Rockwell P, Foster DA (2006) Myc stabilization in response to estrogen and phospholipase D in MCF-7 breast cancer cells. *FEBS Lett* 580(24):5647–5652.

113. Yeh E, et al. (2004) A signalling pathway controlling c-Myc degradation that impacts oncogenic transformation of human cells. *Nat Cell Biol* 6(4):308–18.

114. Ross AJ, et al. (2001) BCLW mediates survival of postmitotic Sertoli cells by regulating BAX activity. *Dev Biol* 239(2):295–308.

115. Sandhu C, et al. (1997) Transforming growth factor beta stabilizes p15INK4B protein, increases p15INK4B-cdk4 complexes, and inhibits cyclin D1-cdk4 association in human mammary epithelial cells. *Mol Cell Biol* 17(5):2458–2467.

116. Schmidt M, et al. (2002) Cell Cycle Inhibition by FoxO Forkhead Transcription Factors Involves Downregulation of Cyclin D. *Mol Cell Biol* 22(22):7842–7852.

117. Schulze WX, Deng L, Mann M (2005) Phosphotyrosine interactome of the ErbB-receptor kinase family. *Mol Syst Biol* 1(1):E1–E13.

118. Sears R, et al. (2000) Multiple Ras-dependent phosphorylation pathways regulate Myc protein stability. *Genes Dev* 14(19):2501–2514.

119. Seoane J, et al. (2001) TGFbeta influences Myc, Miz-1 and Smad to control the CDK inhibitor p15INK4b. *Nat Cell Biol* 3(4):400–8.

120. Shen F, et al. (2008) Downregulation of cyclin D1-CDK4 protein in human embryonic lung fibroblasts (HELF) induced by silica is mediated through the ERK and JNK pathway. *Cell Biol Int* 32(10):1284–1292.

121. Siegel PM, Shu W, Massagué J (2003) Mad upregulation and Id2 repression accompany transforming growth factor (TGF)-β-mediated epithelial cell growth suppression. *J Biol Chem* 278(37):35444–35450.

122. Smalley MJ, Dale TC (2001) Wnt signaling and mammary tumorigenesis. *J Mammary Gland Biol Neoplasia* 6(1):37–52.

123. Sowter HM, Ratcliffe PJ, Watson P, Greenberg AH, Harris AL (2001) HIF-1-dependent regulation of hypoxic induction of the cell death factors BNIP3 and NIX in human tumors. *Cancer Res* 61(18):6669–6673.

124. Stambolic V, et al. (2001) Regulation of PTEN transcription by p53. *Mol Cell* 8(2):317–25.

125. Stasinopoulos IA, et al. (2005) HOXA5-twist interaction alters p53 homeostasis in breast cancer cells. *J Biol Chem* 280(3):2294–2299.

126. Sunters A, et al. (2003) FoxO3a transcriptional regulation of Bim controls apoptosis in paclitaxel-treated breast cancer cell lines. *J Biol Chem* 278(50):49795–805.

127. Sunters A, et al. (2006) Paclitaxel-induced nuclear translocation of FOXO3a in breast cancer cells is mediated by c-Jun NH2-terminal kinase and Akt. *Cancer Res* 66(1):212–220.

128. Taelman VF, et al. (2010) Wnt signaling requires sequestration of glycogen synthase kinase 3 inside multivesicular endosomes. *Cell* 143(7):1136–48.

129. Tait SW, Green DR (2010) Mitochondria and cell death: outer membrane

permeabilization and beyond. *Nat Rev cell Biol* 11(9):621–632.

130. Tamai K, et al. (2004) A Mechanism for Wnt Coreceptor Activation. *Mol Cell* 13(1):149–156.

131. Teuliere J, et al. (2005) Targeted activation of beta-catenin signaling in basal mammary epithelial cells affects mammary development and leads to hyperplasia. *Development* 132(2):267–277.

132. Timoshenko A V., Lala PK, Chakraborty C (2004) PGE2-mediated upregulation of iNOS in murine breast cancer cells through the activation of EP4 receptors. *Int J Cancer* 108(3):384–389.

133. Villunger A, et al. (2003) p53- and drug-induced apoptotic responses mediated by BH3-only proteins puma and noxa. *Science* 302:1036–1038.

134. Vogler M (2012) BCL2A1: the underdog in the BCL2 family. *Cell Death Differ* 19(1):67–74.

135. Wang X, et al. (2004) Identification of a novel function of TWIST, a bHLH protein, in the development of acquired taxol resistance in human cancer cells. *Oncogene* 23(2):474–82.

136. Watanabe O, et al. (2004) Expression of twist and Wnt in human breast cancer. *Anticancer Res* 24(6):3851–3856.

137. Weber JD, Taylor LJ, Roussel MF, Sherr CJ, Bar-Sagi D (1999) Nucleolar Arf sequesters Mdm2 and activates p53. *Nat Cell Biol* 1(May):20–26.

138. Wei MC, et al. (2001) Proapoptotic BAX and BAK: A Requisite Gateway to Mitochondrial Dysfunction and Death. *Science* 292(April):727–730.

139. Weinberg RA (1995) The retinoblastoma protein and cell cycle control. *Cell* 81(3):323–330.

140. Welcker M, et al. (2003) Multisite phosphorylation by Cdk2 and GSK3 controls cyclin E degradation. *Mol Cell* 12(2):381–392.

141. Whitmarsh AJ, Davis RJ (2000) A central control for cell growth. *Nature* 403(6767):255–256.

142. Johnson DG, Schwarz JK, Cress WD, Nevins JR (1993) Expression of transcription factor E2F1 induces quiescent cells to enter S phase. *Nature* 365(6444):349–352.

143. Wu ZL, et al. (2010) Polycomb protein EZH2 regulates E2F1-dependent apoptosis through epigenetically modulating Bim expression. *Cell Death Differ* 17(5):801–810.

144. Xiong S, et al. (2001) Up-regulation of vascular endothelial growth factor in breast cancer cells by the heregulin-beta1-activated p38 signaling pathway enhances endothelial cell migration. *Cancer Res* 61(4):1727–32.

145. Yang W, et al. (2001) Repression of transcription of the p27(Kip1) cyclin-dependent kinase inhibitor gene by c-Myc. *Oncogene* 20(14):1688–1702.

146. Yang J-Y, et al. (2008) ERK promotes tumorigenesis by inhibiting FOXO3a via MDM2-mediated degradation. *Nat Cell Biol* 10(2):138–48.

147. Yang S, Han H (2014) Effect of cycloxygenase-2 silencing on the malignant biological behavior of MCF-7 breast cancer cells. *Oncol Lett* 8(4):1628–1634.

148. Yarden Y, Sliwkowski MX (2001) Untangling the ErbB signalling network. *Nat Rev Mol Cell Biol* 2(2):127–137.

149. Yu J, Zhang L, Hwang PM, Kinzler KW, Vogelstein B (2001) PUMA Induces the Rapid Apoptosis of Colorectal Cancer Cells. *Mol Cell* 7(3):673–682.

150. Zhai D, Jin C, Huang Z, Satterthwait AC, Reed JC (2008) Differential regulation of Bax and Bak by anti-apoptotic Bcl-2 family proteins Bcl-B and Mcl-1. *J Biol Chem* 283(15):9580–9586.

151. Zhou BP, et al. (2001) Cytoplasmic localization of p21Cip1/WAF1 by Akt-induced phosphorylation in HER-2/neu-overexpressing cells. *Nat Cell Biol* 3(3):245–252.

152. Zindy F, et al. (1998) Myc signaling via the ARF tumor suppressor regulates p53-dependent apoptosis and immortalization. *Genes Dev* 12:2424–2433.

153. Zou Y, et al. (2008) Forkhead box transcription factor FOXO3a suppresses estrogen-dependent breast cancer cell proliferation and tumorigenesis. *Breast Cancer Res* 10(1):R21.

154. Zundel W, Giaccia A (1998) Inhibition of the anti-apoptotic PI(3)K/Akt/Bad pathway by stress. *Genes Dev* 12(13):1941–1946.

155. Trimarchi JM, Lees JA (2002) Sibling rivalry in the E2F family. *Nat Rev Mol Cell Biol* 3(1):11–20.

156. Ziello JE, Jovin IS, Huang Y (2007) Hypoxia-Inducible Factor (HIF)-1 regulatory pathway and its potential for therapeutic intervention in malignancy and ischemia. *Yale J Biol Med* 80(2):51–60.

157. Dang C V, Kim J, Gao P, Yustein J (2008) The interplay between MYC and HIF in cancer. *Nat Rev Cancer* 8(1):51–6.

158. Huang LE (2008) Carrot and stick: HIF-alpha engages c-Myc in hypoxic adaptation. *Cell Death Differ* 15(4):672–677.

159. He X, Semenov M, Tamai K, Zeng X (2004) LDL receptor-related proteins 5 and 6 in Wnt/beta-catenin signaling: arrows point the way. *Development* 131(8):1663–77.

160. Brunet A, et al. (2004) Stress-dependent regulation of FOXO transcription factors by the SIRT1 deacetylase. *Science* 303(5666):2011–5.

161. Lehtinen MK, et al. (2006) A conserved MST-FOXO signaling pathway mediates oxidative-stress responses and extends life span. *Cell* 125(5):987–1001.

162. Semenza GL (2003) Targeting HIF-1 for cancer therapy. *Nat Rev Cancer* 3(10):721–32.

163. Won KA, Reed SI (1996) Activation of cyclin E/CDK2 is coupled to site-specific autophosphorylation and ubiquitin-dependent degradation of cyclin E. *EMBO J* 15(16):4182–4193.
